# Supplementary material for: Differential methanogenic archaea-induced TLR8-dependent signaling engages gene classes regulated by NF-κB p65 and STAT1/2
Source: Nat Commun. 2026 Nov 2;17:11116. doi: 10.1038/s41467-026-78885-z (PMC13728207; doi:10.1038/s41467-026-78885-z)
Supplement: Supplementary file 1 [file 41467_2026_78885_MOESM1_ESM.pdf]

Differential methanogenic archaea-induced TLR8-dependent signaling is governed by NF-κB p65- and STAT1/2-controlled gene classes

Fan Xu[1, 2], Johann Sieverding[1], Peiling Zhou[1, 2, 3], Shoshant Sah[1], Viktoria Weinberger[4], Christine Moissl-Eichinger[4], Inken Wohlers[2, 5, 6] and Holger Heine[1, 2*]

[1]Division of Innate Immunity, Research Center Borstel-Leibniz Lung Center, Borstel, Germany.
[2]Airway Research Center North (ARCN), German Center for Lung Research (DZL), Borstel, Germany.

[3]The Second Affiliated Hospital of Zunyi Medical University, Department of Respiratory Medicine, Zunyi, Guizhou, China.

[4]Diagnostic and Research Institute of Hygiene, Microbiology and Environmental Medicine, Medical University of Graz, Graz, Austria.

[5]Research Center Borstel-Leibniz Lung Center, Biomolecular Data Science in Pneumology, Borstel, Germany.

[6]College of Medicine & Health Sciences, Khalifa University for Science and Technology, Abu Dhabi, United Arab Emirates

*Correspondence: hheine@fz-borstel.de (Holger Heine)

Supplementary Figures

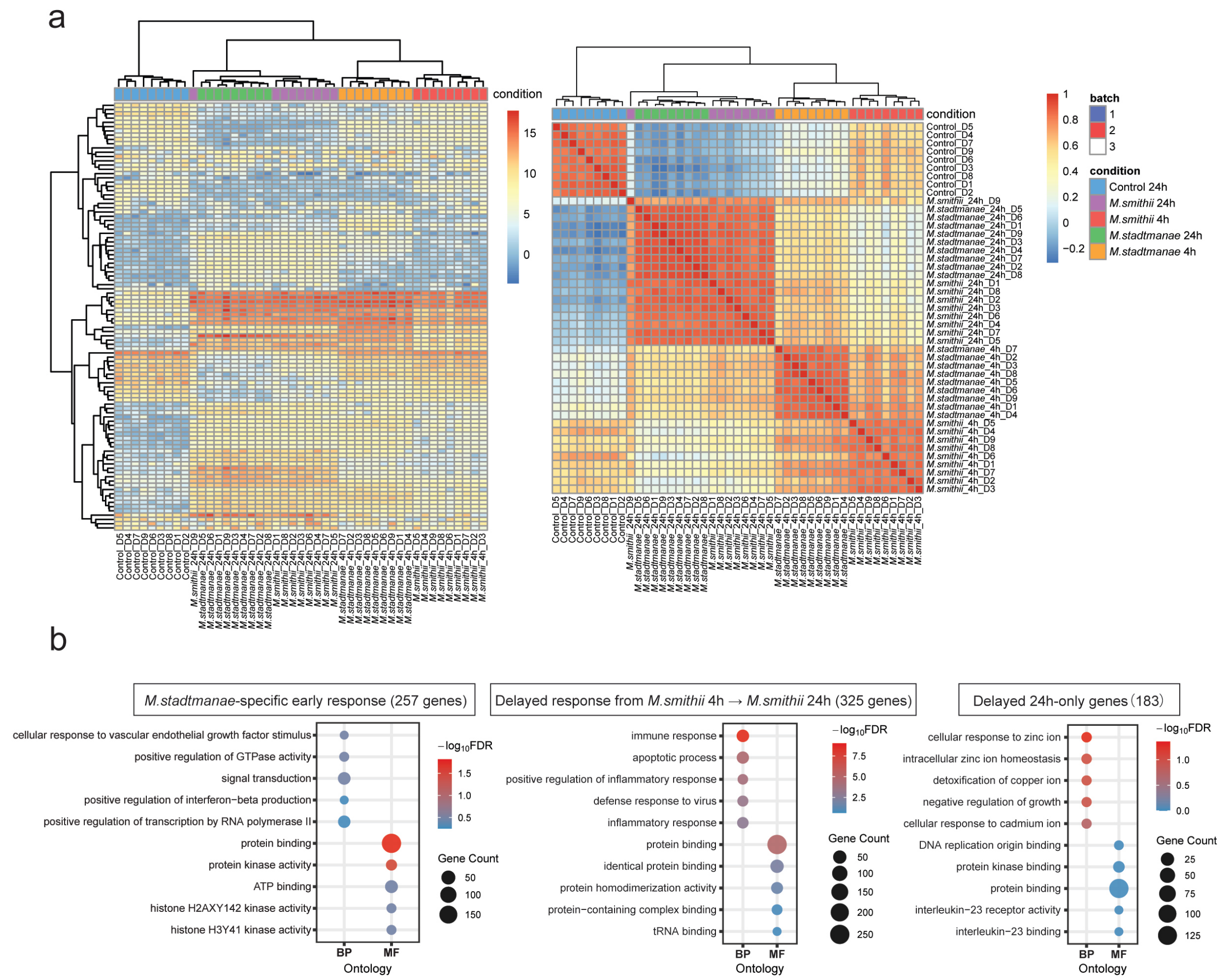

Supplementary Fig. 1. Temporal and species-specific transcriptomic responses to gut methanogenic archaea in PBMCs

a The heatmap on the left shows normalized, $\log_2$-transformed gene expression of the top 100 most variable genes across all PBMCs samples ($n = 9$ donors) stimulated with *M. stadtmanae* or *M. smithii* for 4 h or 24 h, as well as unstimulated controls. The right panel displays a sample-to-sample correlation heatmap based on Pearson correlation coefficients calculated from the top 100 most variable genes displayed on the left. Distances were computed as 1 – correlation. Samples cluster primarily by stimulation condition and time point, reflecting both archaeal species-specific and temporal transcriptional divergence. **b** Bubble plots show the top five enriched GO terms in the BP and MF categories for three gene subsets identified by Venn diagram analysis of differentially expressed genes. The left panel corresponds to genes specifically upregulated during the early response to *M. stadtmanae*; the middle panel to genes enriched in the delayed response to *M. smithii*; and the right panel to genes upregulated only at 24 h in both *M. stadtmanae* and *M. smithii* conditions. Bubble size indicates the number of genes associated with each term, and bubble color reflects the Benjamini–Hochberg FDR-adjusted *P* value for enrichment. GO enrichment was assessed using DAVID's modified Fisher's exact test (EASE score), followed by Benjamini–Hochberg correction. BP denotes gene ontology biological process and MF denotes gene ontology molecular function. *n* denotes the number of independent human donors and therefore represents biological replicates.

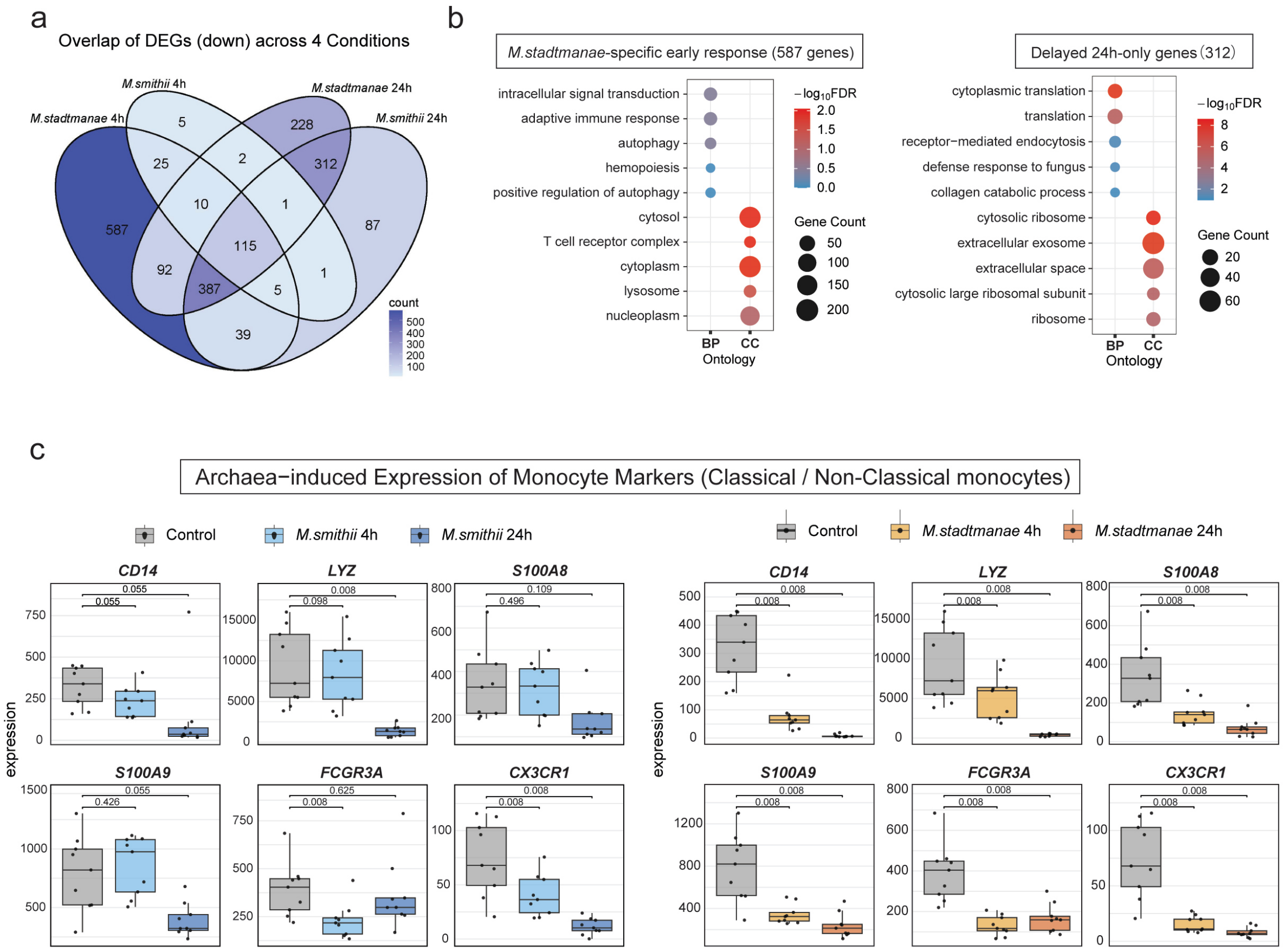

Supplementary Fig. 2. Downregulated gene programs and monocyte marker expression in response to archaea

a A Venn diagram illustrates the overlap of down-regulated DEGs in response to *M. smithii* and *M. stadtmanae* at 4 h and 24 h. **b** Bubble plots show the top five enriched GO terms in the BP and MF categories for two gene subsets identified by Venn diagram analysis of differentially expressed genes. The left panel corresponds to genes specifically down-regulated during the early response to *M. stadtmanae*; and the right panel to genes down-regulated only at 24 h in both *M. stadtmanae* and *M. smithii* conditions. Bubble size indicates the number of genes associated with each term, and bubble color reflects the Benjamini–Hochberg FDR-adjusted *P* value for enrichment. GO enrichment was assessed using DAVID's modified Fisher's exact test (EASE score), followed by Benjamini–Hochberg correction. BP denotes Gene Ontology Biological Process and MF denotes Gene Ontology Molecular Function. **c** Monocyte marker expression in PBMCs (normalized expression). (left panel) *M. smithii* vs control and (right panel) *M. stadtmanae* vs control. Box–jitter plots show normalized expression for CD14, LYZ, S100A8, S100A9, FCGR3A (CD16) and CX3CR1 across the indicated groups (order as plotted: Control, 4 h, 24 h). Each point represents one donor (n = 9 donors). Boxes denote the interquartile range (IQR), center lines the median, and whiskers 1.5× IQR; individual points are overlaid with limited horizontal jitter. Statistical significance was assessed using two-sided paired Wilcoxon signed-rank tests, followed by Holm adjustment of *P* values for multiple comparisons. Holm-adjusted *P* values are shown in the figure.

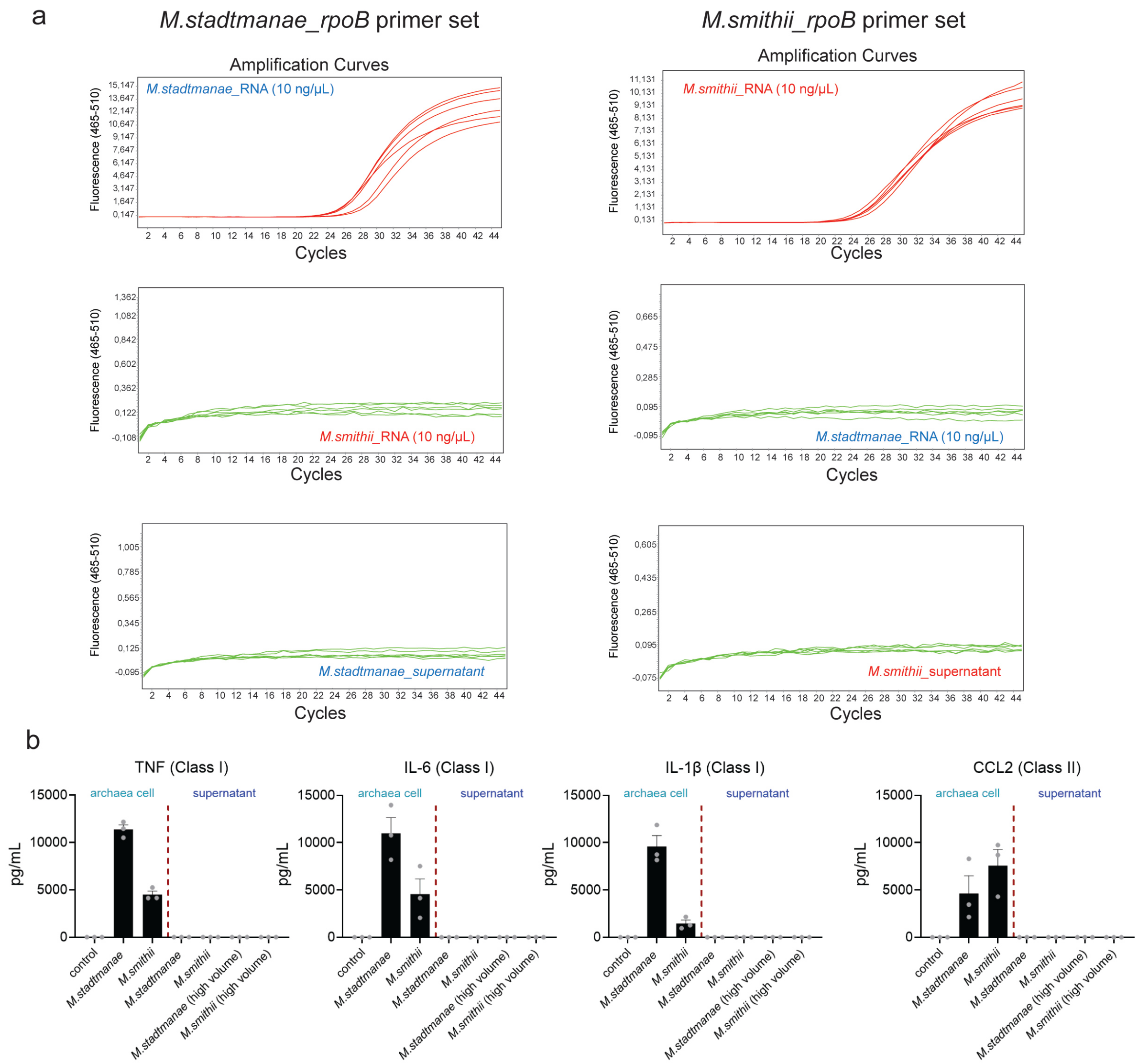

Supplementary Fig. 3. Cell-free archaeal culture supernatants do not account for PBMC activation

a Representative RT-qPCR amplification curves (x-axis: cycle number; y-axis: fluorescence intensity) obtained using two species-specific primer sets targeting *rpoB*. Left: *M. stadtmanae* *rpoB* primers; right: *M. smithii rpoB* primers. Amplification is shown for purified archaeal standard RNA (10 ng/µL) and for rigorously processed archaeal culture supernatants. The experiment was performed using samples from three independent biological replicates (n = 3 biological replicates), with two technical replicates per condition; each amplification curve represents one technical replicate. **b** ELISA quantification of cytokines/chemokines in PBMCs culture supernatants following stimulation with intact archaeal cells or corresponding cell-free supernatants. Supernatants were tested at the standard working volume and at high volume (5× the working volume). Data are shown as mean ± SEM from three independent donors (n = 3 donors), each performed with cells from a different donor. No statistical testing was performed.

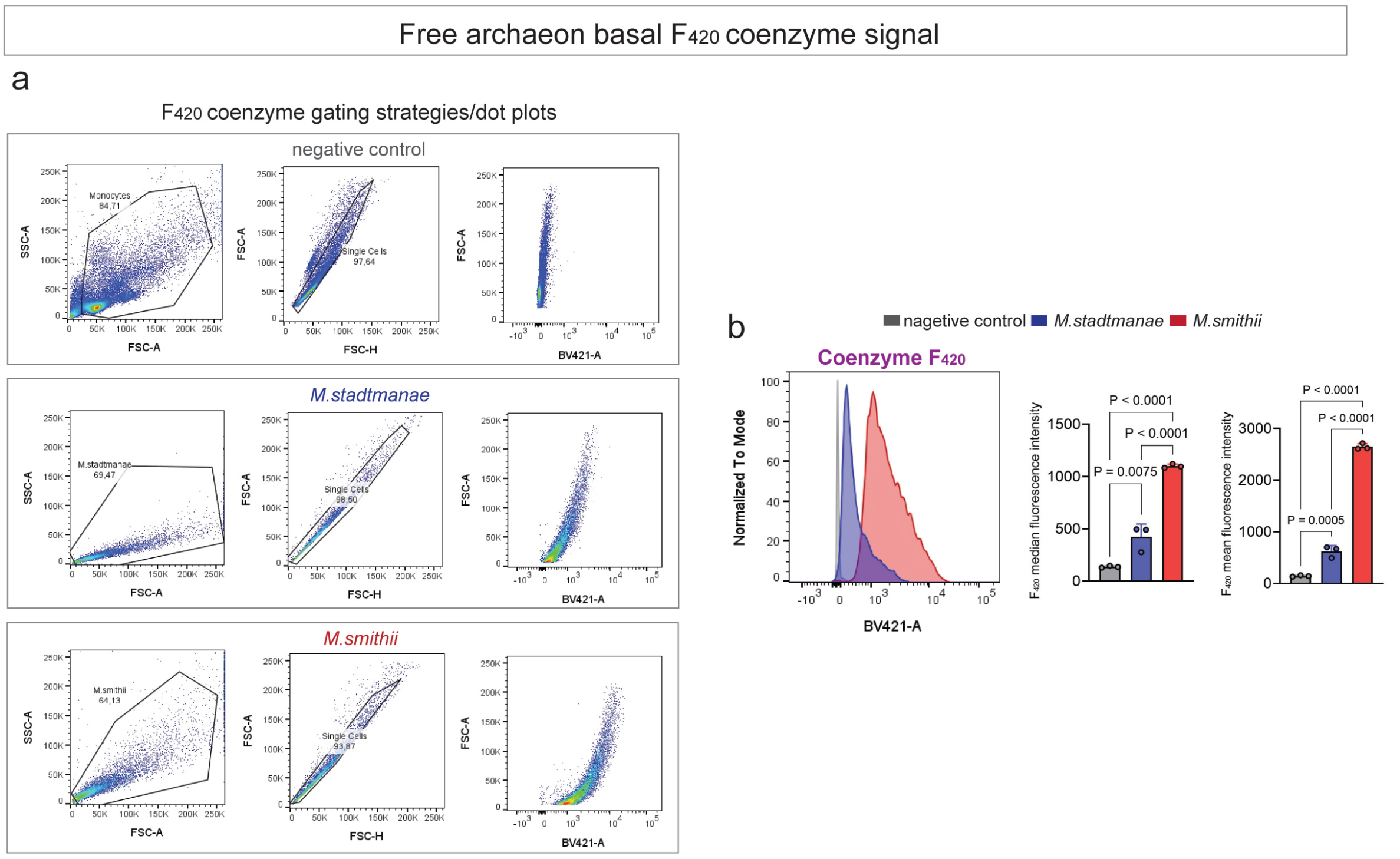

Supplementary Fig. 4. Comparison of intrinsic F_{420} fluorescence in *M. stadtmanae* and *M. smithii*

a Representative flow-cytometry plots showing the gating strategy for detecting the archaeal F_{420} signal. Similar results were obtained from three independent biological replicates (*n* = 3 biological replicates). Cells were first gated on the main cell population based on forward and side scatter, followed by singlets, and then analyzed in the BV421 channel to detect F_{420} fluorescence. Three conditions are shown: negative control (PBMCs without archaea), *M. stadtmanae*, and *M. smithii*. **b** Left panel: Representative overlay histogram showing F_{420} fluorescence in gated cells under the indicated conditions. Equal numbers (20000 cells) of *M. stadtmanae* and *M. smithii* cells were used for F_{420} measurement. Right panel: Quantification of F_{420} mean fluorescence intensity and median fluorescence intensity across three independent experiments (*n* = 3 donors). Each point represents one donor. Data are shown as mean ± SD. Statistical significance was determined by one-way ANOVA followed by Tukey's multiple comparisons test. Exact *P* values are shown in the figure.

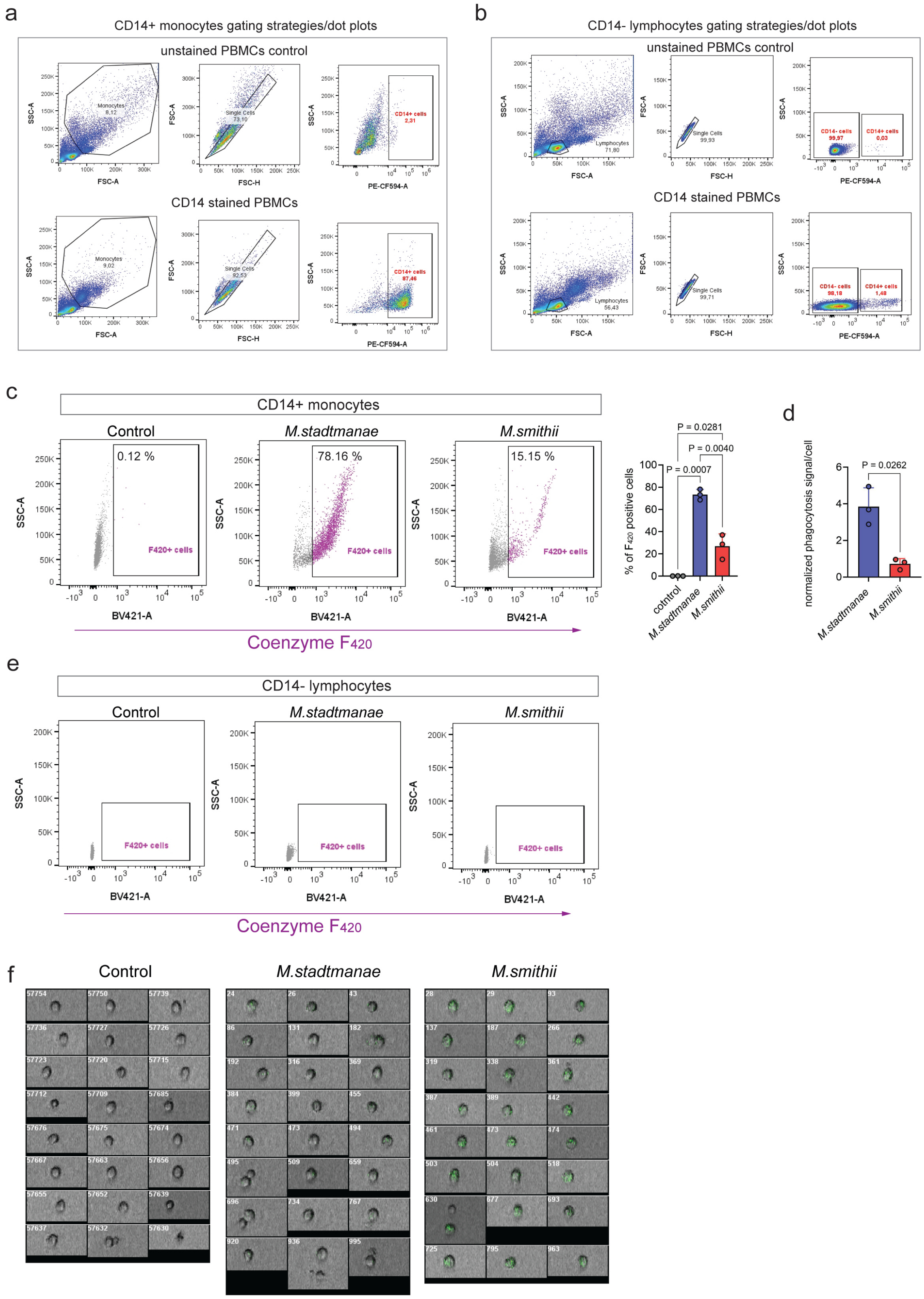

Supplementary Fig. 5. Uptake and intracellular localization of gut archaea in PBMC subsets

a,b Representative flow-cytometry gating strategies for identifying monocytes (**a**) and lymphocytes (**b**). PBMCs were gated by forward scatter (FSC) and side scatter (SSC), followed by singlet selection. Monocytes were identified as CD14^{+} cells and lymphocytes as CD14^{-} cells using gates defined with unstained controls. **c** Left, representative flow-cytometry plots of

coenzyme F_{420} fluorescence in $CD14^+$ monocytes after 4 h stimulation with *M. stadtmanae* or *M. smithii*. Percentages of F_{420}^+ cells are indicated. Right, quantification of F_{420}^+ monocytes. Each dot represents one independent donor ($n = 3$); data are mean ± SD. Statistical significance was assessed using repeated-measures one-way ANOVA followed by Tukey's multiple-comparison test. **d** Normalized phagocytosis signal, calculated for each donor as the mean F_{420} fluorescence associated with $CD14^+$ monocytes divided by the corresponding basal fluorescence of free archaea. Each dot represents one independent donor ($n = 3$); data are mean ± SD. Statistical significance was assessed using a two-sided paired *t*-test. **e** Representative flow-cytometry plots of F_{420} fluorescence in $CD14^-$ lymphocytes after 4 h archaeal stimulation. **f** Representative single-plane images showing F_{420} fluorescence (green) associated with monocytes under unstimulated, *M. smithii*-stimulated and *M. stadtmanae*-stimulated conditions. Punctate F_{420} indicates cell-associated archaeal material. Representative plots and images were obtained from three independent donors ($n = 3$), with comparable results. Exact *P* values are shown in the respective panels.

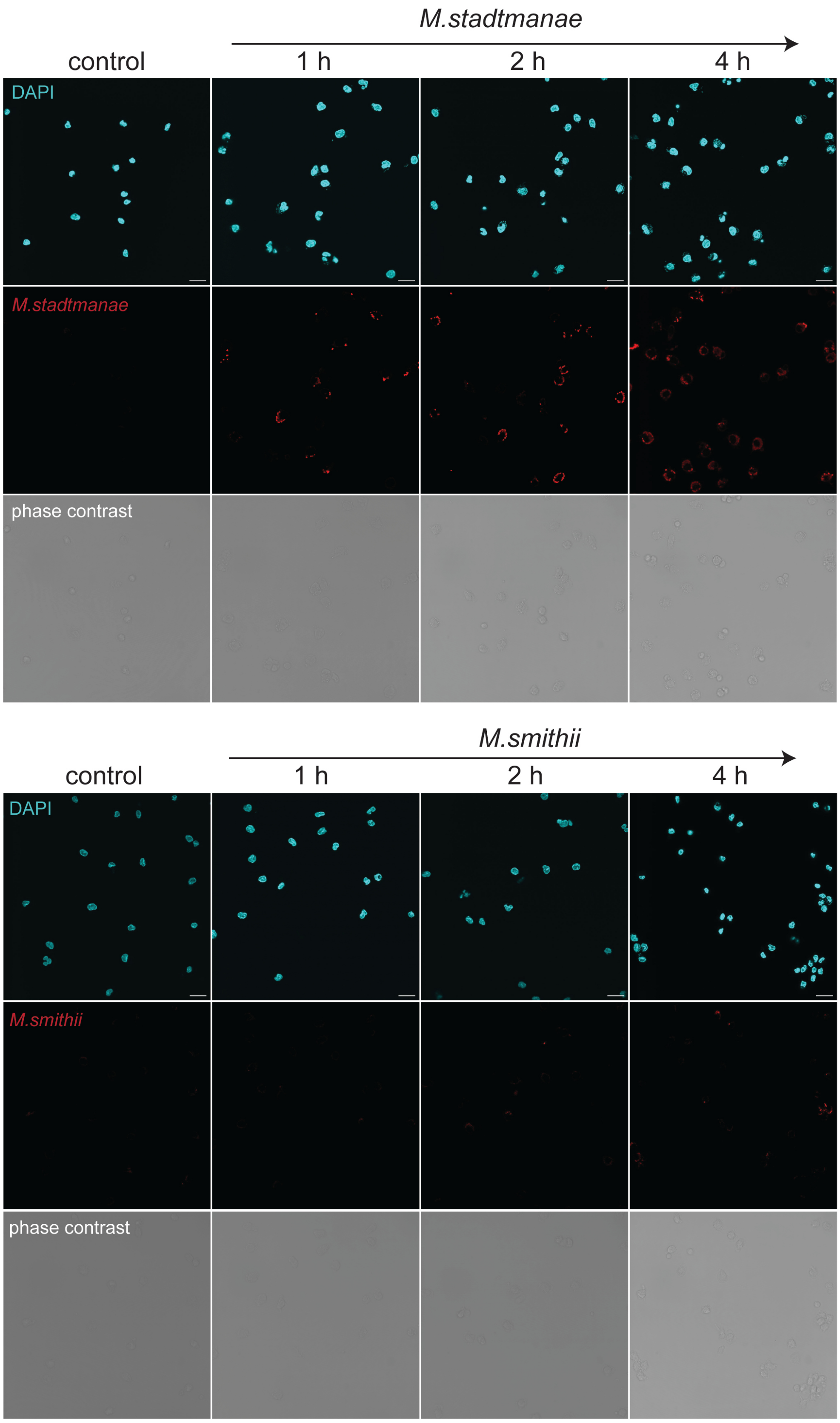

Supplementary Fig. 6. Immunofluorescence analysis of time-dependent archaeal phagocytosis by human PBMC-derived monocytes

Representative immunofluorescence images of human PBMC-derived monocytes left unstimulated or stimulated with archaea for 1, 2, or 4 h. Archaea were detected by

immunostaining and are shown in red, and nuclei were counterstained with DAPI (cyan). Corresponding phase-contrast images are shown. Images were acquired at 63× magnification. Scale bar = 20 μm. The images shown are representative of data obtained from three independent donors (*n* = 3 donors).

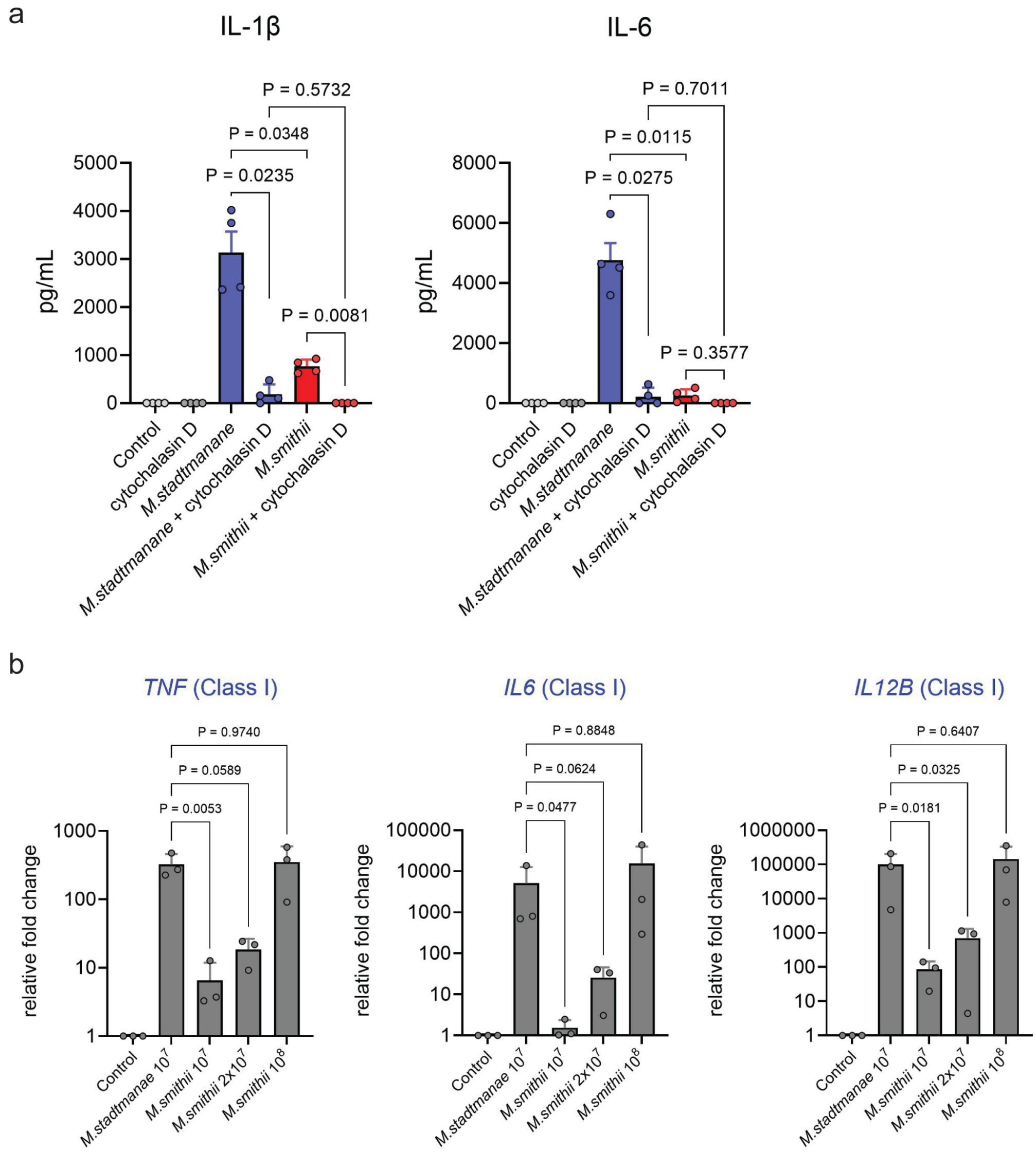

Supplementary Fig. 7. Phagocytosis-dependent cytokine release and dose-dependent class I gene induction in archaeal-stimulated human monocytes

a ELISA was used to quantify IL-1β and IL-6 in culture supernatants from monocytes stimulated with *M. stadtmanae* or *M. smithii* in the presence or absence of the phagocytosis inhibitor cytochalasin D. Each data point represents one independent donor ($n = 4$ donors). Data are shown as mean ± SEM. Statistical significance was assessed using repeated-measures one-way ANOVA followed by Tukey's multiple comparisons test. Exact P values are shown in the figure. **b** RT-qPCR analysis of the expression of selected class I genes in PBMC-derived monocytes stimulated for 4 h with *M. stadtmanae* at 10^7 cells/mL or with *M. smithii* at 10^7, 2 × 10^7, and 10^8 cells/mL. Gene expression levels are presented as fold induction relative to control, normalized to *HPRT*, with the y-axis shown on a $\log_{10}$ scale to reflect the magnitude of induction. Each dot represents one donor, corresponding to one independent donor ($n = 3$ donors). Data are shown as mean ± SD. Statistical significance was assessed using repeated-measures one-way ANOVA followed by Dunnett's multiple comparisons test versus the control condition. Exact P values are indicated above the bars.

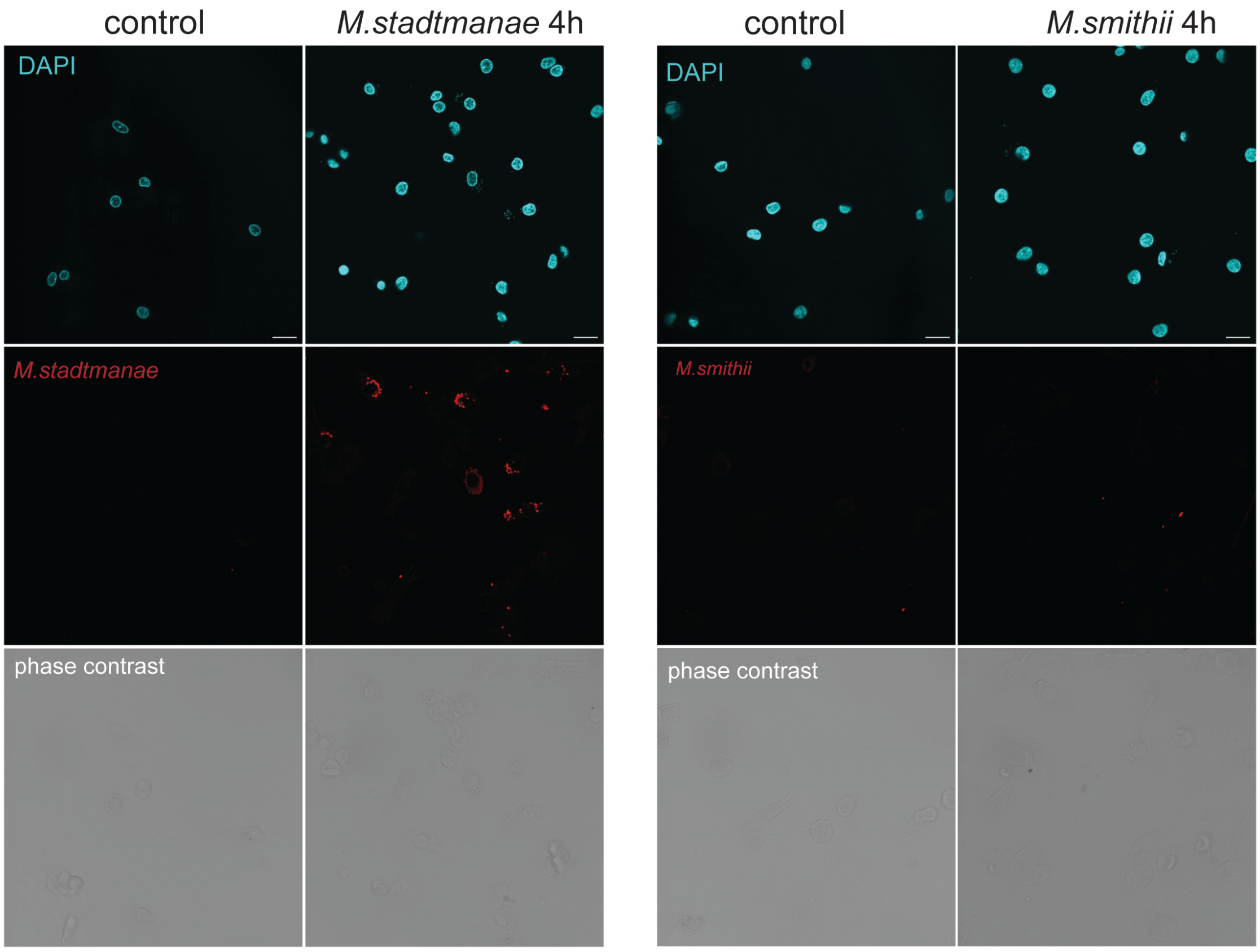

Supplementary Fig. 8. Immunofluorescence analysis of archaeal phagocytosis by human monocyte-derived dendritic cells

Representative immunofluorescence images of human monocyte-derived dendritic cells (moDCs) left unstimulated or stimulated with archaea for 4 h. Archaea were detected by immunostaining and are shown in red, and nuclei were counterstained with DAPI. Corresponding phase-contrast images are shown. Images were acquired at 63× magnification. Scale bar = 20 µm. The images shown are representative of data obtained from three independent donors (*n* = 3 donors).

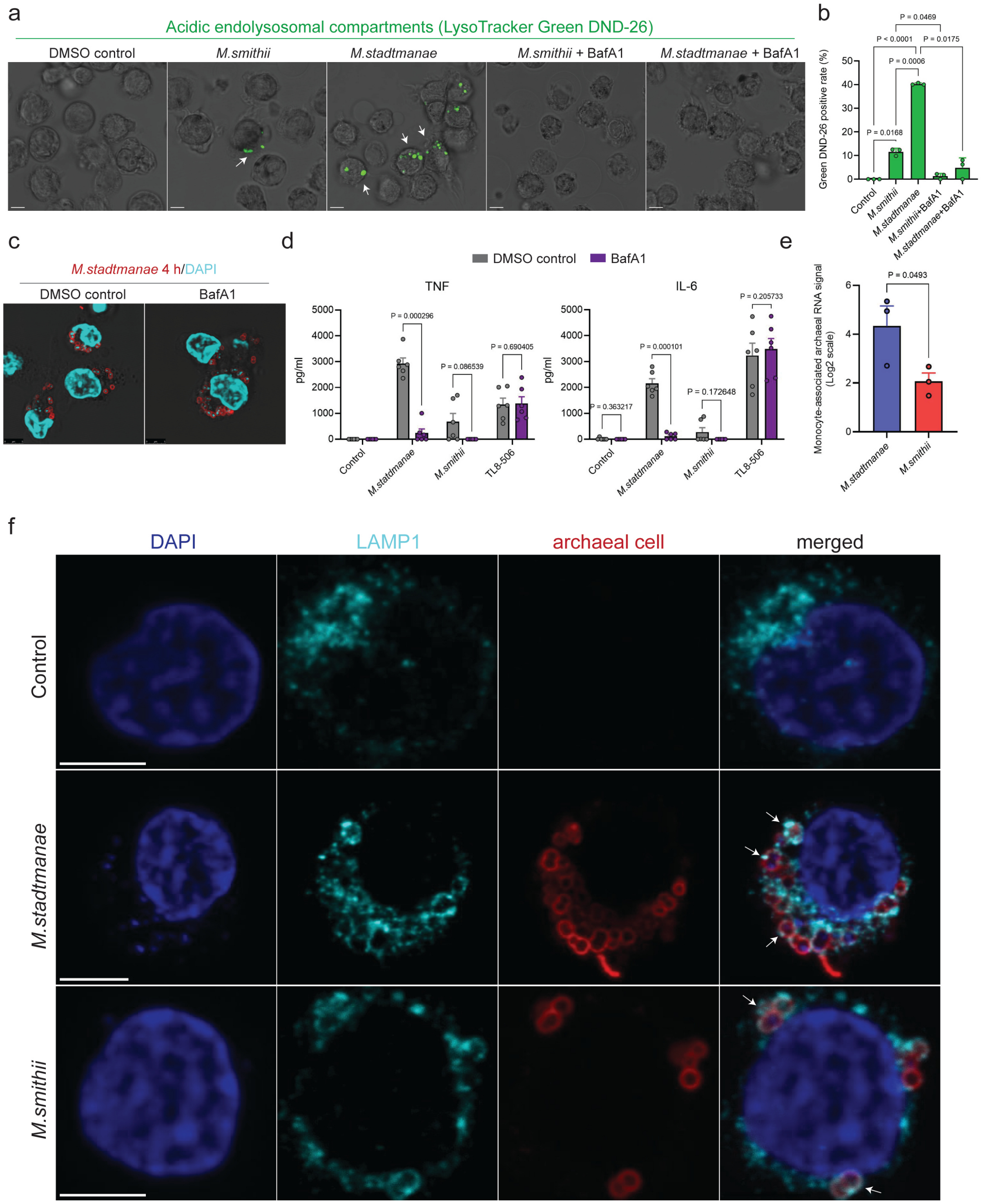

Supplementary Fig. 9. Endolysosomal pathway of internalized archaea in human monocytes

a Representative LysoTracker Green DND-26 images of PBMC-derived monocytes left unstimulated or stimulated with *M. smithii* or *M. stadtmanae* for 4 h in the presence of vehicle (DMSO) or bafilomycin A1 (BafA1). Green puncta indicate acidic endolysosomal compartments. **b** Quantification of LysoTracker-positive monocytes. Each dot represents one independent donor ($n = 3$); data are mean ± SD. Statistical significance was assessed using repeated-measures one-way ANOVA followed by Tukey's multiple-comparison test. **c** Representative immunofluorescence images of *M. stadtmanae* uptake in monocytes treated with DMSO or BafA1. Archaea are shown in red and nuclei in cyan. **d** ELISA quantification of

TNF and IL-6 in supernatants from monocytes stimulated with *M. stadtmanae*, *M. smithii* or TL8-506 in the presence of DMSO or BafA1. Each dot represents one independent donor ($n = 6$); data are mean ± SEM. **e** Species-specific RT–qPCR detection of monocyte-associated archaeal *rpoB* RNA after extensive washing. Signals were normalized to *HPRT* and are presented on a $\log_2$ scale. Each dot represents one independent donor ($n = 3$); data are mean ± SD. For **d** and **e**, statistical significance was assessed using two-sided paired *t*-tests. **f** Representative confocal images showing LAMP1-positive compartments (cyan), cell-associated archaea (red) and nuclei (blue). Arrows indicate selected regions of partial colocalization. Images in **a**, **c** and **f** are representative of three independent donors ($n = 3$), with comparable results. Scale bars, 5 µm. Exact *P* values are shown in the figure.

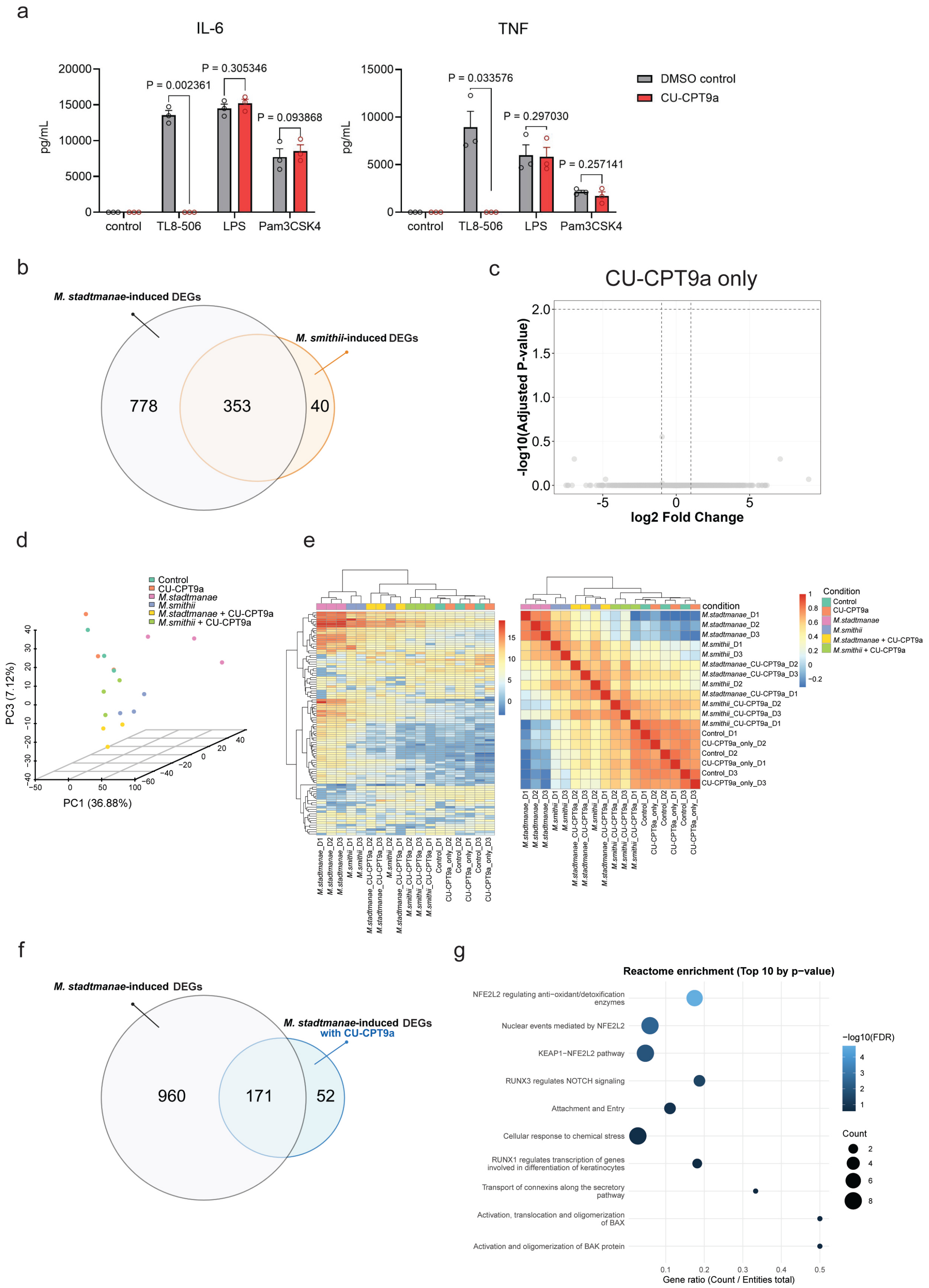

Supplementary Fig. 10. Transcriptomic impact of TLR8 inhibition on archaeal-induced responses in PBMC-derived monocytes

a PBMC-derived monocytes were treated with the TLR8 antagonist CU-CPT9a or vehicle (DMSO) and stimulated as indicated. IL-6 and TNF were quantified by ELISA. Each dot

represents one independent donor ($n = 3$); data are mean ± SEM. Statistical significance was assessed using multiple two-sided paired *t*-tests without correction for multiple comparisons. Exact *P* values are shown. **b** Overlap of genes upregulated after 4 h stimulation with *M. stadtmanae* or *M. smithii*. **c** Differential gene expression following treatment with CU-CPT9a alone. Red, blue and grey indicate upregulated, downregulated and non-significant genes, respectively. Differential expression was assessed using two-sided Wald tests in donor-adjusted Sleuth models, followed by Benjamini–Hochberg correction. DEGs were defined by $|\log_2$ fold change$| \geq 1$ and FDR-adjusted $q < 0.01$. **d** Principal component analysis of samples from the indicated experimental conditions. Colours identify conditions as defined in the figure key. **e** Left, $\log_2$-transformed normalized expression of the top 100 most variable genes. Right, sample-to-sample Pearson correlation based on these genes. Clustering used 1 − Pearson's correlation coefficient as the distance metric; colour scales are defined in the figure keys. **f** Overlap of genes upregulated by *M. stadtmanae* in the absence or presence of CU-CPT9a. **g** Reactome enrichment of genes induced by *M. stadtmanae* that remained unaffected by CU-CPT9a. Enrichment was assessed using a hypergeometric test followed by Benjamini–Hochberg correction. Bubble size indicates gene number and colour represents the FDR-adjusted *q* value. RNA-seq analyses in **b–g** used three independent donors ($n = 3$), representing biological replicates.

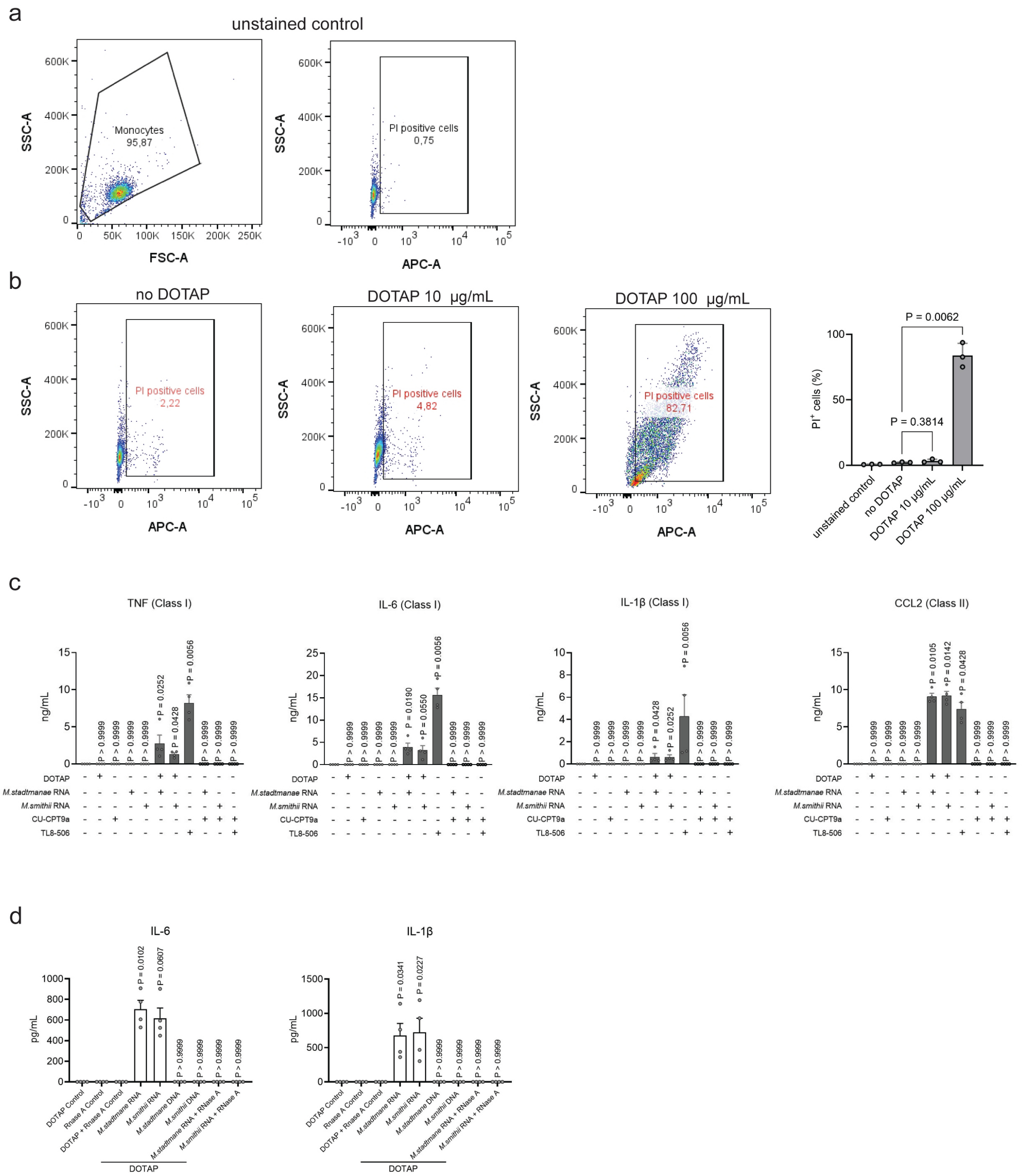

Supplementary Fig. 11. DOTAP enables archaeal RNA sensing at a non-toxic working concentration

a Representative flow-cytometry plots showing the gating strategy for identifying monocytes from three independent donors (*n* = 3 donors). Singlets were first selected, followed by monocyte gating and assessment of PI fluorescence in the APC channel to identify PI-positive (PI^+) cells. **b** Left, representative flow-cytometry plots of PI staining, including an unstained control used to define background fluorescence and establish the PI^+ gate, a no-DOTAP control, DOTAP at the working concentration (10 µg/mL) and DOTAP at 100 µg/mL as a positive toxicity control. The plots shown are representative of three independent donors (*n* = 3 donors). Right, quantification of PI^+ cells from three independent donors (*n* = 3 donors). Data are presented as mean ± SD. Donor-matched samples were analysed using repeated-measures one-way ANOVA followed by two-sided Dunnett's multiple-comparison test. Exact *P* values are

shown in the figure. **c** Primary monocytes were stimulated for 24 h with archaeal total RNA (1 µg/mL), either without a delivery reagent (naked RNA) or complexed with DOTAP, in the presence or absence of the selective TLR8 antagonist CU-CPT9a. Cell-free supernatants were collected and TNF, IL-6, IL-1β, and CCL2 were quantified by ELISA. Data are shown as individual donors with mean ± SEM ($n = 4$ donors). Statistical significance was assessed using the Friedman test followed by Dunn's multiple comparisons test, with each condition compared against the unstimulated control. Exact P values are shown in the figure. **d** ELISA analysis of IL-6 and IL-1β following DOTAP-mediated delivery of archaeal RNA or DNA (1 µg/mL). RNase A treatment was applied only to the indicated RNA conditions. Statistical significance was assessed using the Friedman test followed by Dunn's multiple-comparison test, with the indicated conditions compared with their corresponding controls. Data are presented as individual donors with mean ± SEM ($n = 4$ donors). Exact P values are shown in the figure.

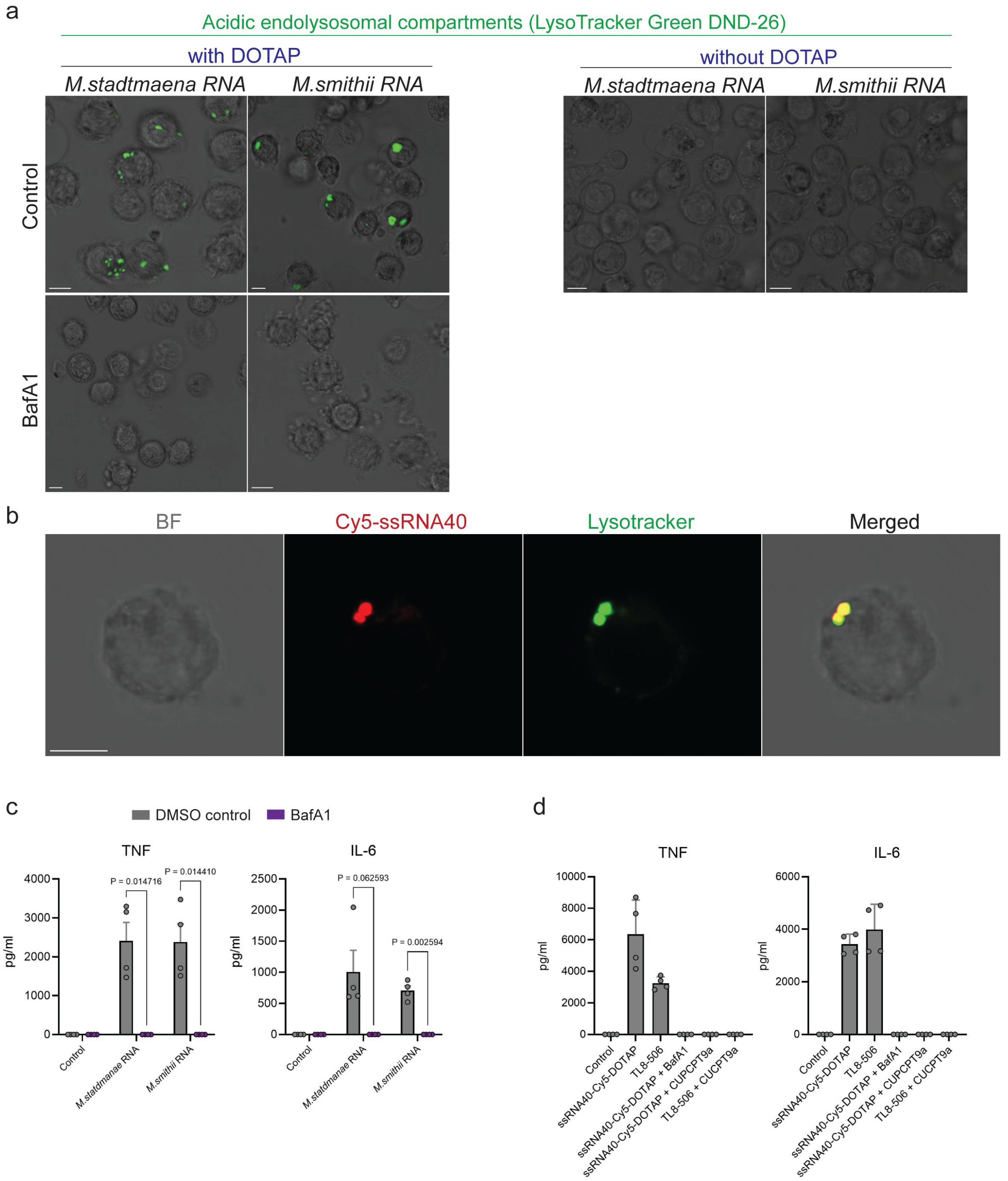

Supplementary Fig. 12. DOTAP-complexed RNA accesses acidified endolysosomal compartments and induces acidification- and TLR8-dependent cytokine responses.

a LysoTracker Green staining of acidic compartments in monocytes treated with DOTAP-complexed archaeal RNA in the presence or absence of bafilomycin A1. The images shown are representative of data obtained from three independent donors ($n = 3$ donors). Scale bars, 5 µm. **b** Representative fluorescence images showing the intracellular localization of DOTAP-complexed Cy5-labelled ssRNA40 and LysoTracker Green-positive acidic compartments. The images shown are representative of data obtained from three independent donors ($n = 3$ donors). Scale bars, 5 µm. **c** Cytokine responses induced by DOTAP-complexed archaeal RNA in the presence or absence of bafilomycin A1. Each dot represents one individual donor, with each donor representing an independent donor ($n = 4$ donors). Data are shown as mean ± SEM. Statistical significance was assessed using a two-sided paired t-test for two-group comparisons.

Exact *P* values are shown in the figure. **d** Cytokine responses induced by DOTAP-complexed Cy5-labelled ssRNA40 in the presence or absence of bafilomycin A1 or the TLR8 inhibitor CU-CPT9a. Data are shown as mean ± SEM. Each data point represents one biologically independent donor (*n* = 4 donors). No statistical testing was performed.

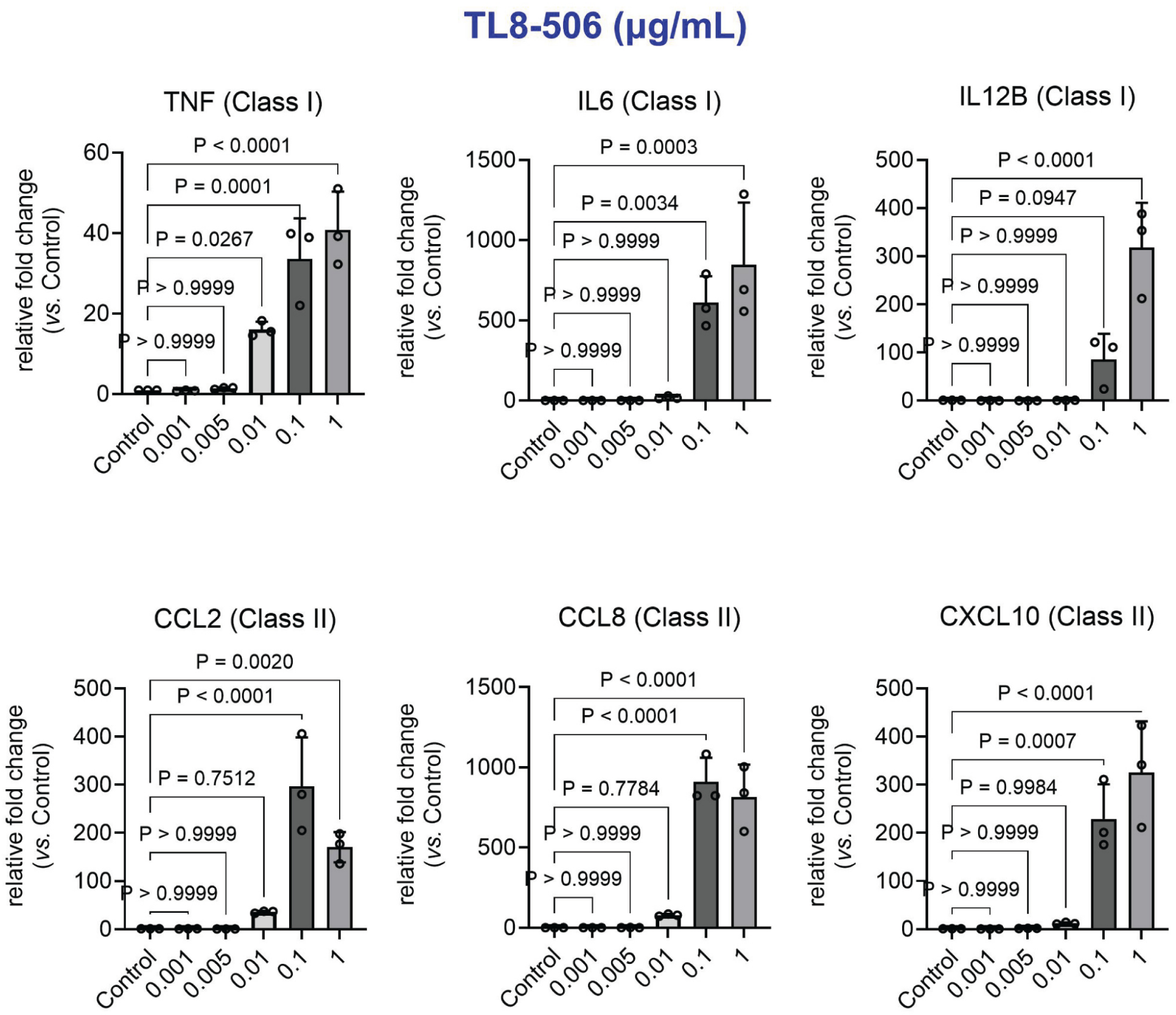

Supplementary Fig. 13. Dose-dependent induction of selected Class I and Class II genes by the synthetic TLR8 agonist TL8-506

RT-qPCR analysis of selected Class I and Class II gene expression in PBMC-derived monocytes stimulated for 4 h with increasing concentrations of the synthetic TLR8 agonist TL8-506 (0.001, 0.005, 0.01, 0.1, and 1 µg/mL). Gene expression levels were normalized to HPRT and are shown as fold change relative to the unstimulated control. Each dot represents one individual donor, with each donor representing an independent donor (n = 3 donors). Data are shown as mean ± SD. Statistical significance was assessed using repeated-measures one-way ANOVA followed by Dunnett's multiple comparisons test, comparing each dose to the unstimulated control. Exact P values are shown in the figure.

a

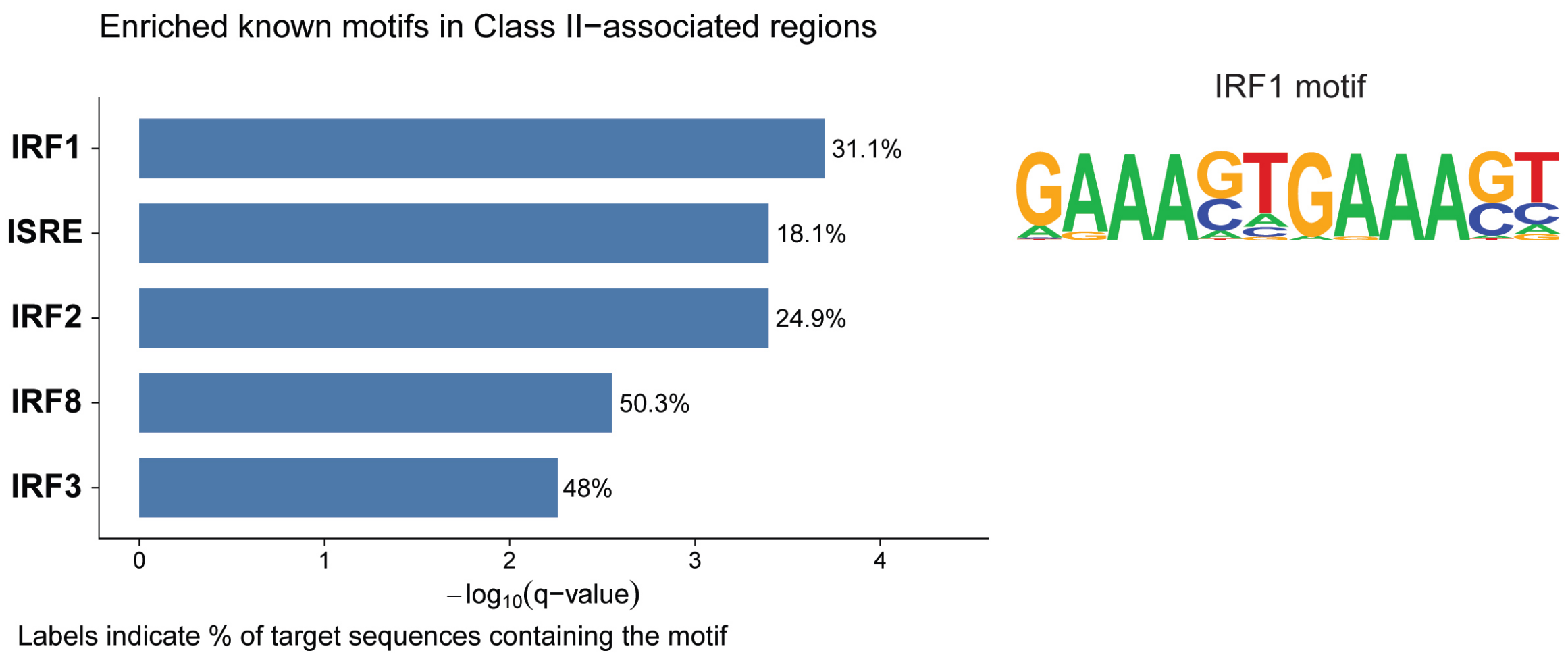

b

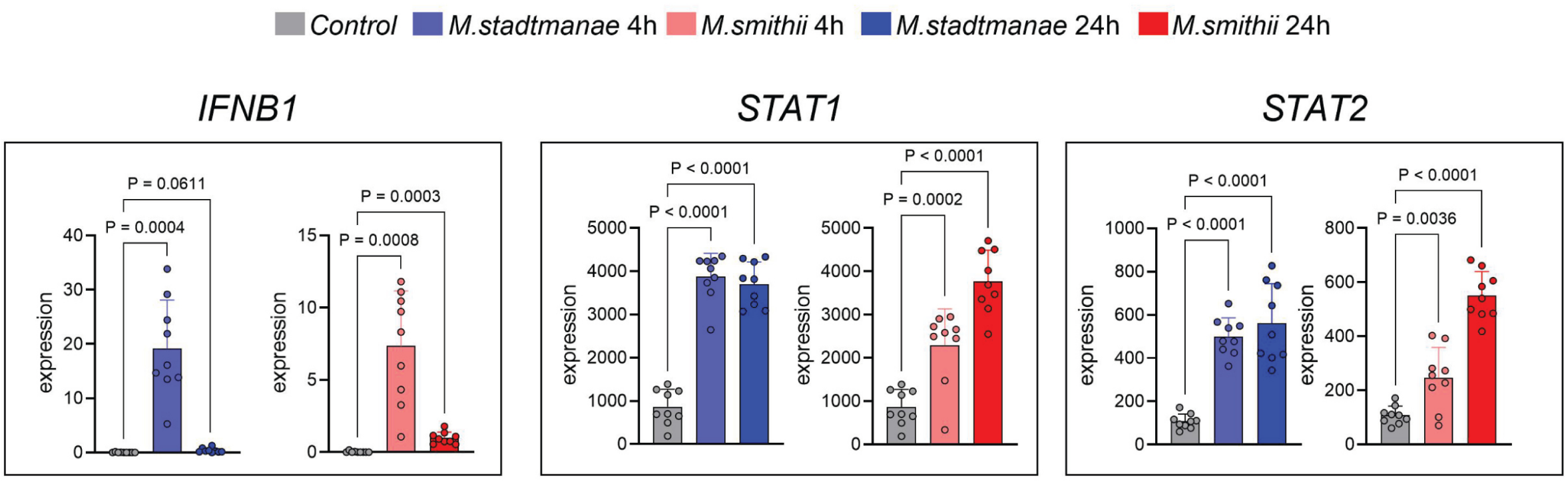

Supplementary Fig. 14. IRF1-associated regulation of Class II genes supported by motif enrichment and IFNB1, STAT1, and STAT2 expression

a Left, HOMER known-motif enrichment analysis of genomic regions spanning −2,000 to +2,000 bp relative to the transcription start sites of Class II genes. Motif enrichment relative to sequence-matched background regions was assessed using the cumulative binomial test implemented in HOMER, followed by Benjamini–Hochberg correction. Only motifs with an adjusted $q < 0.01$ are shown. Bar length represents enrichment significance as $-\log_{10}(q$ value), and percentages indicate the proportion of target sequences containing each motif. Right, sequence logo of the most significantly enriched motif, IRF1. **b** Bar-and-dot plots showing RNA-seq-derived normalized expression of the type I interferon gene IFNB1 together with STAT1 and STAT2 in PBMCs from nine healthy donors following stimulation with *M. stadtmanae* or *M. smithii* for 4 h or 24 h, compared with unstimulated controls. Each dot represents one donor ($n = 9$ donors), and bars indicate mean ± SD. Statistical significance was determined by repeated-measures one-way ANOVA followed by Dunnett’s multiple comparisons test versus the unstimulated control. Exact P values are indicated above the bars.

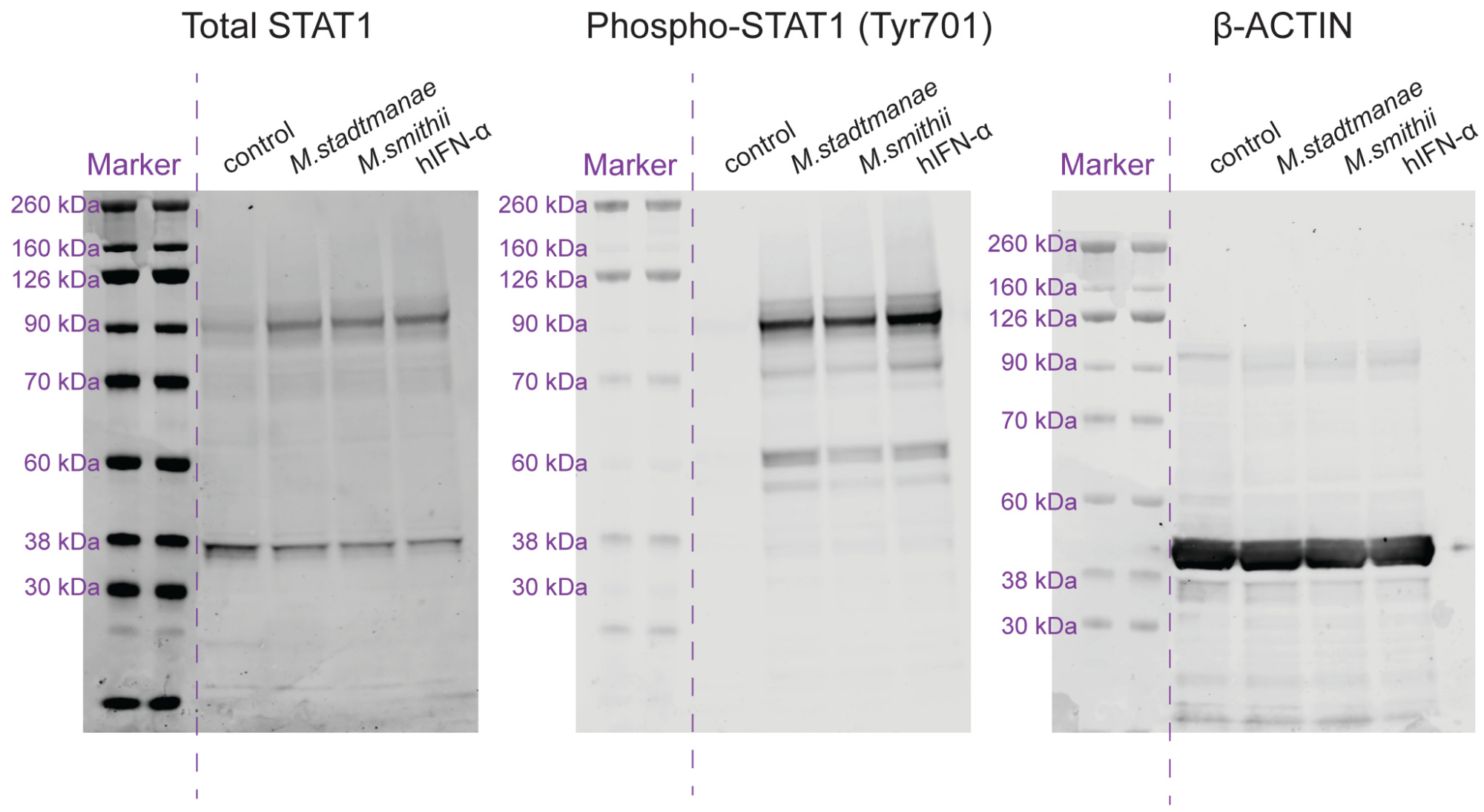

Supplementary Fig. 15. Uncropped western blot images.

Representative uncropped western blot images corresponding to the blots shown in Fig. 7e, including total STAT1, phospho-STAT1 (Tyr701) and β-actin. The experiment was performed using cells from three independent donors ($n = 3$ donors), and the complete uncropped blots from all three donors are provided in the Source Data file.

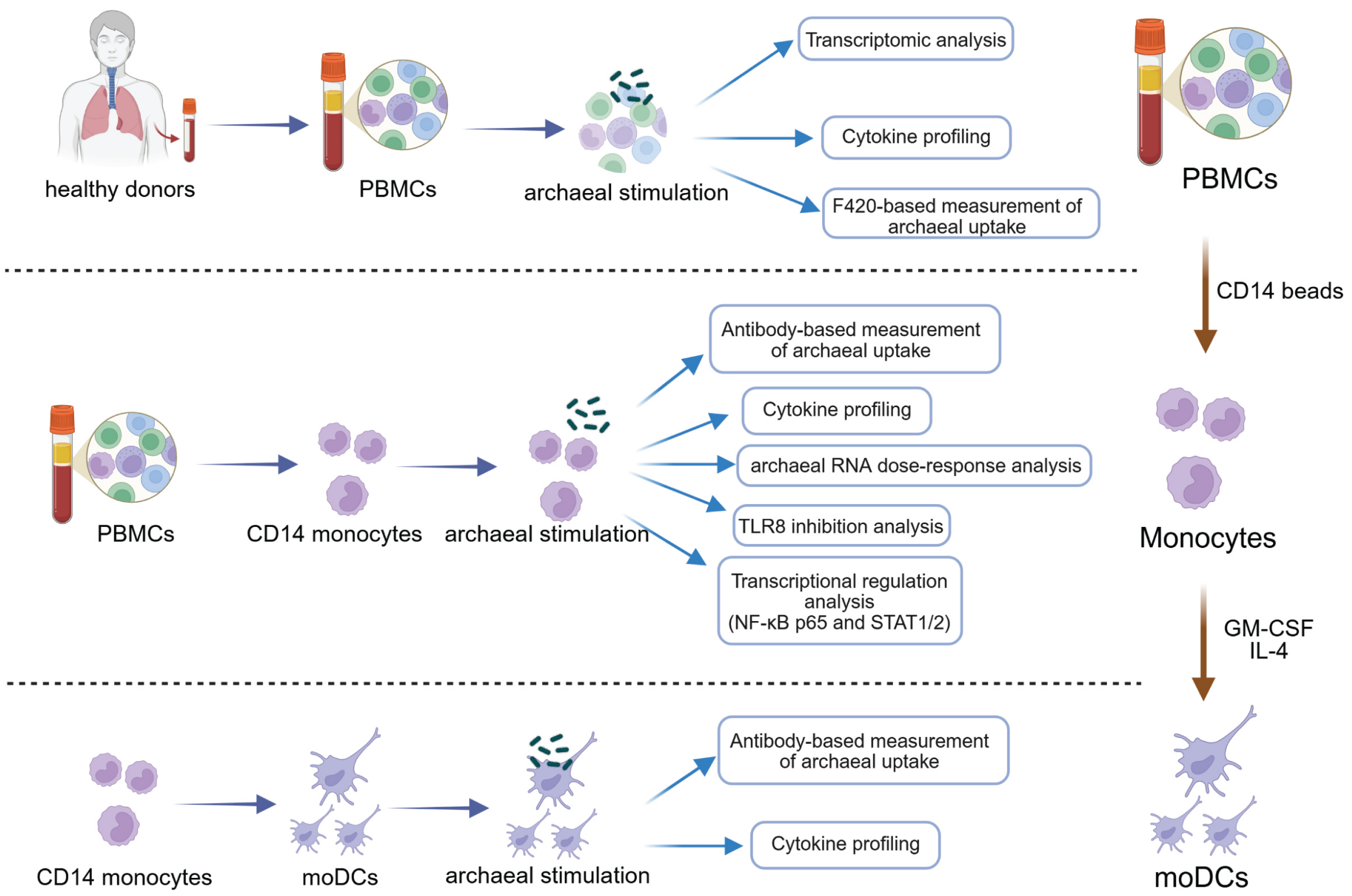

Supplementary Fig. 16. Schematic overview of the study workflow and cellular systems used in this study.

PBMCs isolated from healthy donors were used as the initial discovery system for stimulation with methanogenic archaea, followed by transcriptomic analysis, cytokine profiling, and F_{420}-based measurement of archaeal uptake. Based on these findings, purified $CD14^+$ monocytes were used for mechanistic follow-up experiments, including antibody-based uptake measurements, cytokine profiling, archaeal RNA dose-response analysis, TLR8 inhibition analysis, and transcriptional regulation analysis focused on NF-κB p65 and STAT1/2. In selected experiments, $CD14^+$ monocytes were further differentiated into moDCs using GM-CSF and IL-4 and analyzed for archaeal uptake and cytokine responses. Created in BioRender. Xu, F. (2026) https://BioRender.com/a818mbg.

Supplementary Table 1. List of primers used in this study

Name	Forward	Reverse	Purpose
HPRT	GTCAGGCAGTATAATCCAAAGA	GGGCATATCCTACAACAAACT	RT-qPCR
IL12B	AAAGGAAGATGGAATTTGGTC	TGCTGCTTTTGACACTGAAT	RT-qPCR
IL6	ACTCACCTCTTCAGAACGAATTG	CCATCTTTGGAAGGTTCAGGTTG	RT-qPCR
TNF	CAGCCTCTTCTCCTTCCTGAT	GCCAGAGGGCTGATTAGAGA	RT-qPCR
CCL2	CTGCTCATAGCAGCCACCTT	GCACTGAGATCTTCCTATTGGTG	RT-qPCR
CCL8	CCCTCAGGGACTTGCTCAG	CTCCAGCCTCTGGATAGGAA	RT-qPCR
CXCL10	GAAAGCAGTTAGCAAGGAAAGGT	GACATATACTCCATGTAGGGAAGTGA	RT-qPCR
SLC7A11	TGCTGGGCTGATTTTATCTTCG	GAAAGGGCAACCATGAAGAGG	RT-qPCR
M. stadtmanae rpoB	TGCTTGGTATTTGTGCTGGA	TCCAAGAGCCTGTTTTGTCA	RT-qPCR
M. smithii rpoB	AAGGGATTTGCACCCAACAC	GACCACAGTTAGGACCCTCTGG	RT-qPCR
IL6_promoter	CAACAGGCGGGTCCTGAAAT	AAACAGTGACCTCTGTTGGGC	ChIP-qPCR (p65 and STAT1/2)
TNF_promoter	CGCGATGGAGAAGAAACCGA	TATATGTCCCTGGGGCGAGA	ChIP-qPCR (p65 and STAT1/2)
CCL2_promoter	AAGTGTCTCGTCCTGACCCC	ATTGAAAGCGGGCAGAGGG	ChIP-qPCR (p65 and STAT1/2)
CCL8_promoter	ACCTGGTTCCTTCTTGCCAC	ATTCAGGTGGAATGGGTCCG	ChIP-qPCR (p65 and STAT1/2)
