# Supplementary material for: Differential methanogenic archaea-induced TLR8-dependent signaling engages gene classes regulated by NF-κB p65 and STAT1/2
Source: Nat Commun. 2026 Nov 2;17:11116. doi: 10.1038/s41467-026-78885-z (PMC13728207; doi:10.1038/s41467-026-78885-z)
Supplement: Supplementary file 2 [file 41467_2026_78885_MOESM2_ESM.pdf]

Description of Additional Supplementary Files

Supplementary Data 1. Complete RNA-seq differential-expression results for all genes across the indicated PBMC stimulation conditions.

Supplementary Data 2. Class I and Class II gene assignments and between-species differences in log_2 fold change.

Supplementary Data 3. Complete RNA-seq differential-expression results for monocytes stimulated with archaea in the presence or absence of CU-CPT9a.

Supplementary Data 4. DoRothEA-derived transcription-factor activity scores and associated statistics.
