# Supplementary material for: Differential methanogenic archaea-induced TLR8-dependent signaling engages gene classes regulated by NF-κB p65 and STAT1/2
Source: Nat Commun. 2026 Nov 2;17:11116. doi: 10.1038/s41467-026-78885-z (PMC13728207; doi:10.1038/s41467-026-78885-z)
Supplement: Supplementary file 3 [file 41467_2026_78885_MOESM7_ESM.pdf]

nature portfolio

Corresponding author(s): Holger Heine

Last updated by author(s): Sep 24, 2026

Reporting Summary

Nature Portfolio wishes to improve the reproducibility of the work that we publish. This form provides structure for consistency and transparency in reporting. For further information on Nature Portfolio policies, see our Editorial Policies and the Editorial Policy Checklist.

Statistics

For all statistical analyses, confirm that the following items are present in the figure legend, table legend, main text, or Methods section.

n/a	Confirmed	
☐	☒	The exact sample size (*n*) for each experimental group/condition, given as a discrete number and unit of measurement
☐	☒	A statement on whether measurements were taken from distinct samples or whether the same sample was measured repeatedly
☐	☒	The statistical test(s) used AND whether they are one- or two-sided<br>*Only common tests should be described solely by name; describe more complex techniques in the Methods section.*
☐	☒	A description of all covariates tested
☐	☒	A description of any assumptions or corrections, such as tests of normality and adjustment for multiple comparisons
☐	☒	A full description of the statistical parameters including central tendency (e.g. means) or other basic estimates (e.g. regression coefficient) AND variation (e.g. standard deviation) or associated estimates of uncertainty (e.g. confidence intervals)
☐	☒	For null hypothesis testing, the test statistic (e.g. *F*, *t*, *r*) with confidence intervals, effect sizes, degrees of freedom and *P* value noted<br>*Give P values as exact values whenever suitable.*
☒	☐	For Bayesian analysis, information on the choice of priors and Markov chain Monte Carlo settings
☒	☐	For hierarchical and complex designs, identification of the appropriate level for tests and full reporting of outcomes
☐	☒	Estimates of effect sizes (e.g. Cohen's *d*, Pearson's *r*), indicating how they were calculated

Our web collection on statistics for biologists contains articles on many of the points above.

Software and code

Policy information about availability of computer code

Data collection	Quantitative PCR data were collected and analyzed using LightCycler 480 software (version 1.5.1; Roche). Confocal microscopy images were acquired using LAS AF software (version 2.6; Leica Microsystems). Conventional flow-cytometry data were acquired using BD FACSDiva software (version 9.0; BD Biosciences). Imaging flow-cytometry data were processed and exported using the BD CellView Lens plugin (version 2.0.15; BD Biosciences). Immunoblot signals were acquired using an Odyssey CLx infrared imaging system (LI-COR Biosciences).
Data analysis	Statistical analyses were performed using GraphPad Prism 10.4.1. Flow-cytometry data were analyzed using FlowJo 10.8.1. Microscopy images were quantified using ImageJ 1.54p, and immunoblot band intensities were quantified using Image Studio Lite 5.2. RNA-seq analysis was performed using the Snakemake rna-seq-kallisto-sleuth workflow 2.8.4, including fastp 1.1.0, Kallisto 0.51.1, Sleuth 0.30.2, FastQC 0.12.1 and MultiQC 1.33. Downstream computational analyses were performed in R 4.5.2 using SPIA 2.62.0, decoupleR 2.15.0, DoRothEA 1.22.0, clusterProfiler 4.18.3, pheatmap 1.0.13 and ggplot2 4.0.1. Motif enrichment was performed using HOMER 5. Protein-interaction networks were retrieved using STRING 12.0 and visualized using Cytoscape 3.10.3. RNA-seq reports and corresponding manuscript figures were generated using Quarto 1.7.34. Gene Ontology enrichment was performed using DAVID Bioinformatics Resources 6.8.

For manuscripts utilizing custom algorithms or software that are central to the research but not yet described in published literature, software must be made available to editors and reviewers. We strongly encourage code deposition in a community repository (e.g. GitHub). See the Nature Portfolio guidelines for submitting code & software for further information.

Data

Policy information about availability of data

All manuscripts must include a data availability statement. This statement should provide the following information, where applicable:

- Accession codes, unique identifiers, or web links for publicly available datasets
- A description of any restrictions on data availability
- For clinical datasets or third party data, please ensure that the statement adheres to our policy

The raw RNA sequencing data generated in this study have been deposited in the European Genome Phenome Archive (EGA) under study accession EGAS50000001758. These data are available under controlled access because they are pseudonymised human derived sequencing data, and the raw reads may retain donor specific genetic information that could create a risk of participant reidentification. Researchers may request access through the EGA portal by providing their institutional details and a brief description of the proposed research. Requests are reviewed by the Data Access Committee for project on Archaean cell stimulation. Access is granted following DAC approval of a completed and signed Harmonised Data Access Agreement. Complete applications will normally be reviewed within two weeks. Approved access is granted for the duration of the research project specified in the hDAA. However, the processed RNA sequencing outputs and analysis materials, including workflow reports, MultiQC quality control reports, result tables and plots and analysis input files, result figures and rendered PDFs, are openly available under a CC BY 4.0 licence through FAIRDOMHub project 496 and can be downloaded without an access request. Publicly available ENCODE ChIP-seq datasets used to guide ChIP-qPCR primer design are available under accession codes ENCSR000EBI and ENCSR000EHK. Source data are provided with this paper.

Research involving human participants, their data, or biological material

Policy information about studies with human participants or human data. See also policy information about sex, gender (identity/presentation), and sexual orientation and race, ethnicity and racism.

Reporting on sex and gender	Samples were provided to the investigators in coded form without direct identifiers; exact donor ages and complete information on donor sex were unavailable. Gender identity was not collected. Participants were not selected based on sex. As the study was neither designed nor powered to assess sex-specific effects, no sex-stratified analyses were performed.
Reporting on race, ethnicity, or other socially relevant groupings	Race and ethnicity were not collected
Population characteristics	Primary human PBMCs and monocytes were obtained from healthy adult donors and used as independent biological replicates in ex vivo stimulation experiments. The number of independent donors included in each experiment is specified in the corresponding figure legend. Exact donor ages and complete information on donor sex were unavailable because the samples were provided in an anonymized manner; gender identity was not collected.
Recruitment	Healthy adult blood donors were recruited through the local blood donation service at Forschungszentrum Borstel under approved ethical procedures. All participants provided written informed consent and received compensation for participation. Recruitment was based on donor and sample availability, which may limit demographic representativeness. Matched within-donor comparisons reduced the influence of between-donor variability.
Ethics oversight	Approval for these studies was granted by the Institutional Ethics Committee of the University of Lübeck (Lübeck, Germany; Protocol No. Az. 12-202A) in accordance with the Declaration of Helsinki.

Note that full information on the approval of the study protocol must also be provided in the manuscript.

Field-specific reporting

Please select the one below that is the best fit for your research. If you are not sure, read the appropriate sections before making your selection.

[x] Life sciences [ ] Behavioural & social sciences [ ] Ecological, evolutionary & environmental sciences

For a reference copy of the document with all sections, see nature.com/documents/nr-reporting-summary-flat.pdf

Life sciences study design

All studies must disclose on these points even when the disclosure is negative.

Sample size	No statistical method was used to predetermine sample size. Sample sizes were based on donor availability, established practice for primary human ex vivo immunology experiments, and reproducibility across independent biological replicates. Experiments were performed using 3 to 9 independent donors depending on sample availability and assay type, and exact n values are reported in the corresponding figure legends.
Data exclusions	No data were excluded from the analyses on the basis of the results. Any exclusions due to predefined technical failure or inadequate sample quality, where applicable, are stated in the corresponding Methods or figure legends.
Replication	Findings were verified across independent human donors used as biological replicates and, where applicable, across independent experimental batches conducted on different days. Key observations were reproduced in multiple independent experiments, and representative assays were supported by data from 3 to 9 donors depending on the experiment. All attempts at replication were successful.
Randomization	Randomization was not relevant to this study because ex vivo stimulation experiments were performed on matched aliquots of cells from the

same donor, which were assigned to the different stimulation conditions within each experiment. Donors served as biological replicates across conditions.

Blinding	Blinding was not used during data collection or analysis because samples from the same donor were processed in parallel under predefined experimental conditions and outcomes were measured using standardized assay pipelines. Blinding was not considered relevant for this study.

Reporting for specific materials, systems and methods

We require information from authors about some types of materials, experimental systems and methods used in many studies. Here, indicate whether each material, system or method listed is relevant to your study. If you are not sure if a list item applies to your research, read the appropriate section before selecting a response.

Materials & experimental systems

n/a	Involved in the study
☐	☒ Antibodies
☐	☒ Eukaryotic cell lines
☒	☐ Palaeontology and archaeology
☒	☐ Animals and other organisms
☒	☐ Clinical data
☒	☐ Dual use research of concern
☒	☐ Plants

Methods

n/a	Involved in the study
☒	☐ ChIP-seq
☐	☒ Flow cytometry
☒	☐ MRI-based neuroimaging

Antibodies

Antibodies used	Affinity-purified custom rabbit polyclonal antisera against Methanosphaera stadtmanae and Methanobrevibacter smithii (Davids Biotechnologie GmbH; no catalogue number or clone; 1:100) were used for archaeal staining, followed by Alexa Fluor 594-conjugated goat anti-rabbit IgG (Invitrogen, cat. no. A-11012; 1:100). LAMP1 was detected using mouse monoclonal anti-LAMP1 (clone D4O1S; Cell Signaling Technology, cat. no. 15665; 1:100), followed by Alexa Fluor 488-conjugated goat anti-mouse IgG (Invitrogen, cat. no. A-11001; 1:100). NF-κB p65 was detected using rabbit polyclonal anti-NF-κB p65 (Thermo Fisher Scientific, cat. no. 51-0500; clone not applicable; 1:100 for immunofluorescence and 5 µg per ChIP), followed by Alexa Fluor 546-conjugated goat anti-rabbit IgG (Invitrogen, cat. no. A-11035; 1:200) for immunofluorescence. ChIP was performed using 5 µg per immunoprecipitation of rabbit polyclonal anti-STAT1 (Cell Signaling Technology, cat. no. 9172; clone not applicable), rabbit polyclonal anti-STAT2 (Cell Signaling Technology, cat. no. 4594; clone not applicable), or normal rabbit polyclonal IgG (Cell Signaling Technology, cat. no. 2729; clone not applicable). Immunoblotting was performed using rabbit monoclonal anti-phospho-STAT1 (Tyr701) (clone 58D6; Cell Signaling Technology, cat. no. 9167; 1:1000), mouse recombinant monoclonal anti-STAT1 (clone 1/Stat1; Abcam, cat. no. ab281999; 1:1000), and mouse monoclonal anti-β-actin (clone 8H10D10; Cell Signaling Technology, cat. no. 3700; 1:3000). Secondary antibodies for immunoblotting were IRDye 800CW goat anti-rabbit IgG (LI-COR, cat. no. 926-32211; 1:20,000) and IRDye 680RD goat anti-mouse IgG (LI-COR, cat. no. 926-68020; 1:20,000). Flow-cytometric identification of monocytes used PE-conjugated mouse monoclonal anti-human CD14 (clone M5E2; BioLegend, cat. no. 301805; 1:100).
Validation	The custom affinity-purified rabbit polyclonal antisera against Methanosphaera stadtmanae and Methanobrevibacter smithii were previously validated for species-specific archaeal immunofluorescence staining as described in reference 57 and in the "Generation of archaeal antibodies" and "Immunofluorescence staining and confocal microscopy" sections of the Methods. Anti-LAMP1 (clone D4O1S; Cell Signaling Technology, cat. no. 15665; 1:100), anti-NF-κB p65 (Thermo Fisher Scientific, cat. no. 51-0500), anti-phospho-STAT1 (Tyr701) (clone 58D6; Cell Signaling Technology, cat. no. 9167), anti-STAT1 (clone 1/Stat1; Abcam, cat. no. ab281999), anti-β-actin (clone 8H10D10; Cell Signaling Technology, cat. no. 3700), and anti-CD14 (clone M5E2; BioLegend, cat. no. 301805) were validated by the manufacturers for the respective immunofluorescence, ChIP, immunoblotting or flow-cytometry applications. The corresponding manufacturer product pages and validation data are available using the catalogue numbers provided above. The anti-STAT1 (Cell Signaling Technology, cat. no. 9172), anti-STAT2 (Cell Signaling Technology, cat. no. 4594), anti-NF-κB p65 and normal rabbit IgG antibodies used for ChIP are described in the "Chromatin immunoprecipitation and quantitative PCR" section of the Methods. ChIP specificity was assessed using matched input chromatin and normal rabbit IgG controls, and was further supported by promoter- and stimulus-dependent enrichment patterns. Immunoblot antibody specificity was supported by bands at the expected molecular weights and by stimulus-dependent STAT1 phosphorylation.

Eukaryotic cell lines

Policy information about cell lines and Sex and Gender in Research

Cell line source(s)	Human BLaER1 cells were kindly provided by Thomas Graf (Centre for Genomic Regulation, Barcelona, Spain). BLaER1 is a female-derived human B-cell precursor leukaemia cell line (RRID:CVCL_VQ57). The TLR8-knockout BLaER1 clone was previously generated from the parental BLaER1 line by CRISPR–Cas9-mediated genome editing. Neither line was obtained from a commercial vendor or cell bank; therefore, no catalogue number applies.
Authentication	The BLaER1 cell line was not re-authenticated in this study. A previously generated TLR8 knockout BLaER1 clone established by CRISPR/Cas9-mediated genome editing and validated in earlier work was used as described in the manuscript.
Mycoplasma contamination	BLaER1 cells were not specifically tested for mycoplasma contamination in this study.

Commonly misidentified lines
(See ICLAC register)

No commonly misidentified cell lines listed in the ICLAC register were used in this study.

Plants

Seed stocks

Report on the source of all seed stocks or other plant material used. If applicable, state the seed stock centre and catalogue number. If plant specimens were collected from the field, describe the collection location, date and sampling procedures.

Novel plant genotypes

Describe the methods by which all novel plant genotypes were produced. This includes those generated by transgenic approaches, gene editing, chemical/radiation-based mutagenesis and hybridization. For transgenic lines, describe the transformation method, the number of independent lines analyzed and the generation upon which experiments were performed. For gene-edited lines, describe the editor used, the endogenous sequence targeted for editing, the targeting guide RNA sequence (if applicable) and how the editor was applied.

Authentication

Describe any authentication procedures for each seed stock used or novel genotype generated. Describe any experiments used to assess the effect of a mutation and, where applicable, how potential secondary effects (e.g. second site T-DNA insertions, mosiacism, off-target gene editing) were examined.

Flow Cytometry

Plots

Confirm that:

- [x] The axis labels state the marker and fluorochrome used (e.g. CD4-FITC).
- [x] The axis scales are clearly visible. Include numbers along axes only for bottom left plot of group (a 'group' is an analysis of identical markers).
- [x] All plots are contour plots with outliers or pseudocolor plots.
- [x] A numerical value for number of cells or percentage (with statistics) is provided.

Methodology

Sample preparation

PBMCs were isolated from heparinized peripheral blood of healthy adult donors by density-gradient centrifugation using Biocoll. For archaeal-uptake experiments, PBMCs were stimulated with Methanosphaera stadtmanae or Methanobrevibacter smithii (1×10^7 cells ml^{-1}) for 4 h at 37 °C and 5% CO_2 and stained with PE-conjugated anti-CD14 to identify monocytes. For viability experiments, primary PBMC-derived monocytes were exposed to the indicated DOTAP concentrations and stained with propidium iodide.

Instrument

Flow cytometric acquisition was performed on a BD FACSymphony A3 analyzer. Imaging flow cytometry was performed on a BD FACSDiscover S8 system.

Software

Flow-cytometry data were acquired using BD FACSDiva v9.0 and analysed using FlowJo v10.8.1. Imaging flow-cytometry data were processed and exported using the BD CellView Lens plugin v2.0.15.

Cell population abundance

No cell sorting was performed. In PBMC uptake experiments, $CD14^+$ monocytes and $CD14^-$ cells were quantified within the total PBMC population, and the percentages of $F420^+$ events were determined within the respective gated populations. In viability experiments, the percentage of PI^+ cells was quantified within the gated monocyte population.

Gating strategy

Cells were first gated on the main population using FSC and SSC parameters, followed by singlet gating. $CD14^+$ and $CD14^-$ populations were defined using PE-conjugated anti-CD14, with gate boundaries established using unstained controls. $F420^+$ events were quantified within the corresponding CD14 gates using the BV421 channel; F420 positivity was defined relative to unstimulated controls. For viability experiments, monocytes were gated after singlet selection, and PI^+ cells were identified in the APC channel using unstained controls to define background fluorescence. Identical gates were applied across matched samples.

- [x] Tick this box to confirm that a figure exemplifying the gating strategy is provided in the Supplementary Information.
