# Supplementary material for: Differential methanogenic archaea-induced TLR8-dependent signaling engages gene classes regulated by NF-κB p65 and STAT1/2
Source: Nat Commun. 2026 Nov 2;17:11116. doi: 10.1038/s41467-026-78885-z (PMC13728207; doi:10.1038/s41467-026-78885-z)
Supplement: Supplementary file 4 [file 41467_2026_78885_MOESM8_ESM.pdf]

nature portfolio

Peer Review File

Differential methanogenic archaea-induced TLR8-dependent signaling is governed by NF-κB p65- and STAT1/2-controlled gene classes

Corresponding Author: Professor Holger Heine

Parts of this Peer Review File have been redacted as indicated to remove third-party material.

This file contains all reviewer reports in order by version, followed by all author rebuttals in order by version.

Attachments originally included by the reviewers as part of their assessment can be found at the end of this file.

Version 0:

Reviewer comments:

Reviewer #1

(Remarks to the Author)
In the manuscript entitled, "Differential methanogenic archaea-induced TLR8-dependent signaling is governed by NF-κB p65- and STAT1/2-controlled gene classes," Xu et al are reporting results from their study investigating how human associated archaea are sensed by the mammalian innate immune system. Some archaea species have been linked to human diseases but the molecular basis for host-pathogen interactions between humans and archaea remain poorly understood. As such, this is a well-timed study with a strong biomedical impact addressing a glaring knowledge gap. Authors start with performing a transcriptomic analysis of PBMCs stimulated with the two archaeal species for 4 or 24 hours. They find species and stimulation duration dependent differences in transcriptional changes in PBMCs. They used the STRING database to look for protein-protein interactions-based relationships between a conserved core of 443 differentially expressed genes and found STAT1 as one of the bridge nodes linking larger clusters. Additionally, pathway analysis revealed several pathways involved in antiviral signaling, which is consistent with authors' contention that archaeal RNA is the primary innate immune stimulator. Authors then classified genes into several classes, such as early and late responders. To account for species specific differences authors used flow cytometry and microscopy to measure phagocytosis efficiency of the two archaea which shows that Methanosphaera stadtmanae was more efficiently phagocytosed. It would be interesting to find out the mechanism behind the differential phagocytosis, probably in a separate follow up study. To understand the mechanism behind the differential kinetic regulation of early and late responders to Methanobrevibacter smithii stimulation (ClassI/II), authors tested archaeal RNA transfection followed by qPCR and ChIP-qPCR. They find that late responders were activated at higher concentrations, which is consistent with more efficient phagocytosis of Methanosphaera stadtmanae resulting in achieving higher RNA concentrations early in case of stimulation with Methanosphaera stadtmanae. They also find that NF-κB is differentially bound to the two classes of genes. Overall, this is an interesting study. Novelty is derived from investigations of these processes in the context of innate immune sensing of archaea. However, the study lacks several controls, there are concerns about physiological relevance of experimental setup, and the data can be explained by alternative models as outlined below.
Major points:
1. Species specific differences in pathogenicity of the two methanogens (Methanosphaera stadtmanae and Methanobrevibacter smithii) is interesting. Authors propose that this could be due to differential engagement of NF-κB signaling by the two. Is there any difference in methane production or tolerance for oxygen between the two species? The reason why I am asking this question is that the NLRP1 inflammasome can be activated by reductive stress. If there is a difference in methane production or tolerance for oxygen, it might be associated with extracellular environments more or less conducive to NLRP1 inflammasome activation. This in turn would result in differences in levels of secreted IL-1β and IL-18, which would have differential effect on NF-κB signaling. Essentially, differential engagement of the NLRP1 inflammasome can also explain the observed differences between the two species.
2. Do the two archaea remain intact in the culture conditions used for stimulations? The reason why I am asking is that if they do not remain intact, and spill their intracellular contents, insights generated from the experiments are not going to have much relevance with respect to mechanisms of innate immune sensing. Another reason is that for archaeal RNA to become

an innate immune ligand it has to come out of the cell. This will only happen if cells do not remain intact. Chemically, I am hard pressed to think of a difference in archaeal, bacterial and animal RNA other than 5`cap and modifications in tRNA. So basically, if author's model is correct, innate immune sensing of archaea only happens when archaeal cells are ruptured.
3. The difference between the kinetics of transcriptional suppression and activation (Class I and Class II) can also be explained by differences in stabilities of the two archaeal cells in the culture conditions. This can easily be tested by qPCR analysis of archaeal RNA in cell culture supernatants. As a control, authors should spin down archaeal cells and use only the supernatants for stimulating PBMCs to rule out contributions of contaminants.
4. This could be an alternative mechanism more in agreement with the author's proposed model. It is possible that RNA from the two archaea is differentially modified. What are those modifications?
5. What is the effect of phagocytosis inhibitors on innate immune stimulation by the two archaea? As a control, authors should spin down archaeal cells and use only the supernatants in combination with beads to rule out the contribution of contaminants to differences in phagocytosis.
6. Lines 671-674: I am not sure there is evidence to support this statement. It may be true, but there is no evidence at this point given that TLR8 inhibitor + agonist combination did suppress inflammatory gene expression. Lines 681-683 are more reasonable.
7. TLR8 is facing outside the cell. Why do the authors need to transfect RNA to engage TLR8? Cytosolic delivery of RNA should engage other innate immune sensors. What is the effect of just stimulating cells with archaeal RNA? Phagocytosis and rupture of archaeal cells would release RNA which can be sensed by TLR8 in endosomes. Transfection of RNA with DOTAP does not capture this process. Just adding archaeal RNA to cells and incubating them would lead to activation of TLR8, although this would require large amounts of RNA.
8. Why are authors using DOTAP for RNA transfections? In this reviewer's experience, DOTAP induces significant toxicity in myeloid cells. It is recommended to use reagents that are optimized for RNA transfections.
Minor points:
1. Lines 80-82: There are some reports of viral proteins being detected by TLR2.
2. TNF-α is now named TNF.

Reviewer #2

(Remarks to the Author)
SUMMARY

The manuscript by Xu et al. examines differential transcriptomic responses of PBMCs and monocytes to two species of human commensal methane-producing archaea. The main observation in the first portion of study are that M. smithii and M. stadtmanae induce overlapping but distinct gene expression programs. Specifically, M. stadtmanae induces stronger expression of many immune response genes at 4 hours compared to M. smithii. By 24 hours, the transcriptomes have largely converged.

The remainder of the manuscript examines the mechanisms of these differential gene expression responses. The authors show that
- TLR8 is required for almost all gene expression responses to both species
- M. stadtmanae is taken up by monocytes more efficiently than M. smithii
- Stimulation with purified archaeal RNA from the two species produces the same gene expression responses, indicating that the differences require whole-cell archaea.
- Thus, it appears that the two archaea species signal through identical mechanisms and the difference in transcriptomic response can be fully explained by the slower and overall lower phagocytic uptake of M. smithii.
- M. stadtmanae activates NFkB more efficiently, leading to greater p65 occupancy at Class I genes. In contrast, STAT1/2 occupancy at target genes is comparable between the two species.
- This is likely explained by the fact that TLR8 activates NFkB and Type I IFN / ISGF3 gene expression programs with different dose-sensitivity. The NFkB pathway has a higher dynamic range, whereas ISGF3-gene expression responses saturate at a lower dose

Overall, this is a well-executed study with notable strengths:
- The main dataset is derived from a fairly large number of donors (n=9), and reproducibility is impressively high between the donors
- The timecourse measurements, especially in Figure 3, are informative
- The proposed mechanism is interesting and has broader implications for our understanding of TLR8 signaling

MAIN AREAS FOR IMPROVEMENT:

If one of the goals of the study is to elucidate differences between M. smithii and M. stadtmanae (as suggested by the title and abstract), then more attention needs to be paid to differences that would explain differential TLR8 engagement (since the data indicate that equivalent activation of TLR8 using archaeal RNA produces the same transcriptomic responses). For example:
- The Introduction should provide more background on the differences between the two species
- Why is M. stadtmanae taken up by monocytes more efficiently? What is known in the literature? If this is not known, can the authors identify proteins (or genes) that are more highly expressed in one species than another?
- The entire study is done using a fixed dose of archaea (10^7 per ml). Can the gene expression differences between the two species be overcome by increasing the amount of M. smithii?

The proposed mechanism is that weak engagement of TLR8 is sufficient to strongly induce ISGF3 target genes but not NFkB target genes. This is a very interesting finding that would be generalizable to all TLR8-activating ligands and warrants additional investigation.
- Does stimulation with different doses of a synthetic TLR8 ligand recapitulate the gene expression differences seen with the two archaea species? If the hypothesis is correct, low dose TLR8 agonist should preferentially induce Class II genes.
- ChIP-qPCR is not the best way to examine differential activation of NFkB vs. IRF-ISGF3 axes. Additional biochemical studies would provide more direct evidence of differential pathway activation. For example: nuclear localization of IRF3, STAT1, STAT2, and IRF9 (similar to the p65 immunofluorescence in Fig. 7a), or Western blots (e.g. for phospho-IRF3, phospho-STAT1, etc), or EMSA to measure DNA-binding activity of NFkB vs. ISRE elements.
- Reporting expression of type I IFN genes would be more informative than STAT1 and STAT2 expression (current Fig 7e)
- Are there other studies on dose-dependent TLR responses that suggest expression of ISGF3 target genes saturate at lower doses?

ADDITIONAL MINOR CONCERNS
- PBMCs are not likely to ever interact with these archaea in vivo, and the data indicate that CD14+ cells are more important than lymphocytes. Why did the authors use PBMCs rather than monocytes or moDCs for the whole project?
- The transcription factor motif analysis (Figure 7C) is an important piece of data that sheds light on the difference between Class I and II genes. This should be presented earlier, preferably as part of Figure 2, before any of the mechanistic experiments.
- Are there IRF motifs that appear in the analysis of Figure 7C? If so, they should be highlighted in the figure. If not, their absence should be considered in the Discussion because ISGF3 target genes should be enriched IRF motifs.
- The writing would benefit from further editing, especially in the Introduction. The writing could be more concise and clearer.
- The interpretation of Figure 4 data (Lines 606-614) is somewhat vague
- Figure 6b statistical comparisons should be between species, not between doses.
- What was the source of the human PBMCs? Healthy volunteers, patients at a clinic, etc?
- Line 143 – for the moDC culture methods, please at least include what growth factors were used (with doses) and how many days monocytes were grown in culture before they were considered to be DCs.
- Line 349 reports 10261 genes with “significant response (adjusted $p < 0.01$).” I initially interpreted that to mean genes that are induced by stimulation with either archaea, which would be an exceptionally large number of induced genes. However, Figure 1b volcano plots report a much smaller (and more reasonable) number of induced genes. What is the explanation for this discrepancy? Maybe just a wording mistake in Line 349?
- Did the authors filter their RNA-seq data for genes that are not expressed in moDCs (this step is not mentioned in the Methods)?
- What type of multiple comparisons correction is being used to adjust p-values for RNA-seq data?
- Figure 1c – 3D PCA is difficult to interpret. Would prefer to see two separate 2D PCA plots (e.g. PC1 vs PC2, PC1 vs PC3)
- Figure 1 legend – define “BP” and “MF” within the legend text
- Figure 3e, one of the microscopy labels is incorrect, should be 4h instead of 2h

Reviewer #3

(Remarks to the Author)
The manuscript submitted by Xu and colleagues entitled “Differential methanogenic archaea-induced TLR8-dependent signaling is governed by NF-κB p65- and STAT1/2-controlled gene classes” deals with divergent M. stadtmanae and M. smithii induced immune responses in human PMBCs and monocytes. They propose a model in which differential uptake leads to quantitative differences in intracellular archaeal RNA, sensed predominantly via TLR8, resulting in high-threshold NF-κB–driven ‘Class I’ responses versus low-threshold STAT1/STAT2–driven ‘Class II’ responses. In general, the authors address an exciting topic.

The topic is novel and of broad interest to immunological research, and the experimental design is generally strong and internally consistent. Overall, the manuscript is too long and the paragraphs should be much more focused. In addition, some major and minor issues should be addressed to strengthen the mechanistic conclusions and improve clarity.
Major points:
1. The use of “Class I” and “Class II” to describe transcriptional programs is potentially misleading, as these terms are strongly associated with MHC class I/II in immunology. This may cause confusion, particularly given the immune context of the study. The authors should consider renaming these programs or explicitly clarifying that this classification is unrelated to antigen presentation.
2. Pharmacologic inhibition with CU-CPT9a strongly suggests TLR8 as the dominant upstream sensor. However, reliance on a single antagonist is insufficient to fully establish specificity. Concerning the data in Figure 5b: what are the TLR8-independet effects? Additional controls demonstrating that non-TLR8 pathways are unaffected (e.g. responses to other TLR agonists) or orthogonal validation (genetic knockdown) would substantially strengthen this claim. Conclusions regarding TLR8 exclusivity should be phrased more cautiously.
3. The DOTAP-mediated RNA delivery experiments are central to the threshold model, but the manuscript lacks sufficient controls to exclude confounding by RNA contaminants (e.g. endotoxin, DNA, protein).
4. The use of F420 autofluorescence as a label-free readout of archaeal uptake is interesting (Fig. 3a); however, F420 is not exclusive to archaea and can also be found in certain bacterial taxa (for example: https://pmc.ncbi.nlm.nih.gov/articles/PMC7267189/). This raises the possibility of misinterpretation, particularly in mixed-cell or complex biological settings. To strengthen the claim that the detected signal reflects archaeal internalization, the authors should consider complementary approaches, such as staining with archaeal-specific antibodies or other archaeal markers,

to independently validate uptake. Similarly, in Fig. 3e: DAPI was used to visualize DNA, but this signal is not necessarily archaea-derived.
5. Conceptually, the question is whether PBMCs are the primary cells that encounter archaea regularly during steady-state or infection? What about intestinal immune cells? The authors should clarify why they were investigating PBMCs and also name related limitations.

Minor points:
1. The manuscript alternates between PBMC-based discovery and monocyte-focused mechanistic studies. While logical, this should be stated more explicitly, ideally with a schematic summarizing which experiments were performed in which cell populations and with how many donors.
2. Donor numbers, pairing, and statistical tests should be clearly stated for each figure panel. Where possible, effect sizes in addition to p values should be provided.

This manuscript presents a compelling and conceptually coherent model for archaeal sensing in human monocytes, linking uptake efficiency, RNA dose, TLR8 signaling, and promoter-level transcriptional control. Addressing the points above, particularly terminology, RNA controls, and the strength of mechanistic claims, will significantly improve clarity and rigor and will strengthen the impact of the study.

Reviewer #4

(Remarks to the Author)
The human gastrointestinal tract has a substantial number of archaea. However, the interactions between archaea and the immune system and their roles in physiology remain poorly understood. In this paper, the authors report the systematic, time-resolved transcriptomic dissection of human PBMCs' responses to the two dominant gut methanogens. They show that class II genes have a lower activation threshold than class I genes. Previous papers, including those of the authors, have demonstrated the immunogenicity of M. smithii and M. stadtmanae in human peripheral blood mononuclear cells (PBMCs) and monocyte-derived dendritic cells, and that archaeal immune recognition is primarily driven by RNA50. Therefore, a significant drawback of this study is that the conceptual advancement derived from the results appears to be limited. Additional weaknesses are described below.

Major comments:

They nicely show using purified RNA that the immunostimulatory capacity of M. stadtmanae and M. smithii RNA is broadly similar. As a result, they conclude that the stronger Class I output seen with intact M. stadtmanae is best explained by enhanced cellular uptake rather than intrinsically “stronger” RNA. This raises the key question: what mechanism explains the different uptake rates of these two strains? Does M. smithii actively inhibit phagocytosis or does M. stadtmanae express a molecule that facilitates phagocytosis? Addressing this mechanism will strengthen the paper.

Given that these two methane-forming archaeal strains reside in the gut, examining how they interact with gut epithelial cells (or even intestinal epithelial cell lines) and whether they stimulate immune responses in these cells can provide valuable basic insights.

Based on the FACS plot, M. smithii internalization rate was still lower than that of M. stadtmanae. The authors should also look at MFI to assess the phagocytosis rate per cell.

Did the intrinsic differences in F420 fluorescence for M. smithii and M. stadtmanae (MFI ratio M. stadtmanae/M. smithii = 0.38) affect the interpretation of FACS assessment of internalization?

The experiments with CU622 CPT9a TLR8 antagonist should include at least one negative control.

Reviewer #5

(Remarks to the Author)
I co-reviewed this manuscript with one of the reviewers who provided the listed reports. This is part of the Nature Communications initiative to facilitate training in peer review and to provide appropriate recognition for Early Career Researchers who co-review manuscripts.

Version 1:

Reviewer comments:

Reviewer #1

(Remarks to the Author)
Please see attachment.

Reviewer #2

(Remarks to the Author)
The authors have responded to the critiques with adequate additional data and reasonable counterarguments. The amount of work that has gone into this study is to be commended.

The intrinsic limitations of this study remain: it straddles two interesting findings without going deeply into either. The first finding is that M. smithii has lower uptake in phagocytes than M. stadtmanae, resulting in weaker induction of some genes ("class I"). The second finding is that M. stadtmanae preferentially induces NFkB but not IRF target genes at the same dose as M. smithii. Neither finding is given a fully satisfying mechanistic explanation, and the results of the new experiments (especially different doses of archaea and TLR8 agonist alone) did not shed much light on the mechanistic details.

That being said, I believe the authors are aware of the limitations of the study and do reasonably consider them in the discussion. Ideally, the manuscript could be made more concise for improved readability.

Reviewer #3

(Remarks to the Author)
The authors have performed additional experiments and provided many new suitable data. Overall, this significantly strengthens the manuscript and the majority of my raised concerns were addressed.

Reviewer #4

(Remarks to the Author)
The revised manuscript is satisfactory.

Reviewer #5

(Remarks to the Author)
I co-reviewed this manuscript with one of the reviewers who provided the listed reports. This is part of the Nature Communications initiative to facilitate training in peer review and to provide appropriate recognition for Early Career Researchers who co-review manuscripts.

Version 3:

Reviewer comments:

Reviewer #1

(Remarks to the Author)
Authors have addressed all my concerns. I recommend acceptance. I also want to thank them for a constructive response to my critiques.
This is not a critique for this manuscript but a suggestion for the future: the data in the manuscript is also compatible with a model where a chemical difference (maybe an RNA modification) between archaeal RNA and mammalian RNA makes some form(s) of archaeal RNA a TLR8 ligand. If it turns out to be true, authors would have made another truly fundamental contribution to the field of innate immunity.

Reviewer #2

(Remarks to the Author)
The authors have provided the needed data to support their claims. The additional dose response data in Supplemental Figure 7 is a strength, and the addition of Figure 8 clearly presents the main findings: M. smithii is taken up less efficiently and activates TLR8 less strongly than M. stadtmanae, and weak vs strong induction of TLR8 produces distinct gene expression programs driven by STATs and NFkB, respectively. These findings are supported by an extensive amount of experimental work. The study leaves two intriguing questions that deserve to be investigated in follow-up work: 1) Why is M. smithii phagocytosed less efficiently than M. stadtmanae? 2) Why does weak TLR8 activation favor STAT signaling over NFkB signaling?

My only feedback to improve the manuscript at this time are:
- Figure 8 would be better if the third layer was flipped, so that "high input" is on the left and "low input" is on the right, so that this layer is aligned vertically with the two species of archaea
- The manuscript is very long; it would improve readability if the authors can find places to make the writing more concise, especially in the discussion

Overall, I have no remaining concerns about the scientific rigor or the interpretation of the data. The central question for the editor to decide is whether the significance of this work is suitable for this journal.

Open Access This Peer Review File is licensed under a Creative Commons Attribution 4.0 International License, which permits use, sharing, adaptation, distribution and reproduction in any medium or format, as long as you give appropriate credit to the original author(s) and the source, provide a link to the Creative Commons license, and indicate if changes were made.
In cases where reviewers are anonymous, credit should be given to 'Anonymous Referee' and the source.
The images or other third party material in this Peer Review File are included in the article's Creative Commons license, unless indicated otherwise in a credit line to the material. If material is not included in the article's Creative Commons license and your intended use is not permitted by statutory regulation or exceeds the permitted use, you will need to obtain permission directly from the copyright holder.
To view a copy of this license, visit https://creativecommons.org/licenses/by/4.0/

Point-by-point response

Reviewer 1

In the manuscript entitled, "Differential methanogenic archaea-induced TLR8-dependent signaling is governed by NF-κB p65- and STAT1/2-controlled gene classes," Xu et al are reporting results from their study investigating how human associated archaea are sensed by the mammalian innate immune system. Some archaea species have been linked to human diseases but the molecular basis for host-pathogen interactions between humans and archaea remain poorly understood. As such, this is a well-timed study with a strong biomedical impact addressing a glaring knowledge gap. Authors start with performing a transcriptomic analysis of PBMCs stimulated with the two archaeal species for 4 or 24 hours. They find species and stimulation duration dependent differences in transcriptional changes in PBMCs. They used the STRING database to look for protein-protein interactions-based relationships between a conserved core of 443 differentially expressed genes and found STAT1 as one of the bridge nodes linking larger clusters. Additionally, pathway analysis revealed several pathways involved in antiviral signaling, which is consistent with authors' contention that archaeal RNA is the primary innate immune stimulator. Authors then classified genes into several classes, such as early and late responders. To account for species specific differences authors used flow cytometry and microscopy to measure phagocytosis efficiency of the two archaea which shows that Methanosphaera stadtmanae was more efficiently phagocytosed. It would be interesting to find out the mechanism behind the differential phagocytosis, probably in a separate follow up study. To understand the mechanism behind the differential kinetic regulation of early and late responders to Methanobrevibacter smithii stimulation (ClassI/II), authors tested archaeal RNA transfection followed by qPCR and ChIP-qPCR. They find that late responders were activated at higher concentrations, which is consistent with more efficient phagocytosis of Methanosphaera stadtmanae resulting in achieving higher RNA concentrations early in case of stimulation with Methanosphaera stadtmanae. They also find that NF-κB is differentially bound to the two classes of genes. Overall, this is an interesting study. Novelty is derived from investigations of these processes in the context of innate immune sensing of archaea. However, the study lacks several controls, there are concerns about physiological relevance of experimental setup, and the data can be explained by alternative models as outlined below.

Response: We thank the reviewer for the positive overall assessment and helpful comments. We agree that careful controls are important, and we have added the requested control experiments in the revised manuscript. The details are provided in the point-by-point responses below.

We also agree that the different uptake/phagocytosis observed for *M. stadtmanae* and *M. smithii* is an important point. In the revised manuscript, we added further experiments to strengthen this aspect. However, the precise mechanisms underlying these differences are still under investigation and remain part of our ongoing work.

Major points:

1. Species specific differences in pathogenicity of the two methanogens (Methanosphaera stadtmanae and Methanobrevibacter smithii) is interesting. Authors propose that this could be due to differential engagement of NF-κB signaling by the two. Is there any difference in methane production or tolerance for oxygen between the two species? The reason why I am asking this question is that the NLRP1 inflammasome can be activated by reductive stress. If there is a difference in methane production or tolerance for oxygen, it might be associated with extracellular environments more or less conducive to NLRP1 inflammasome activation. This in turn would result in differences in levels of secreted IL-1β and IL-18, which would have differential effect on NF-κB signaling. Essentially, differential engagement of the NLRP1 inflammasome can also explain the observed differences between the two species.

Response: We thank the reviewer for this interesting suggestion. Regarding oxygen tolerance, available data do not support a marked difference between the two methanogens (NPJ Biofilms Microbiomes, 2022; PMID: 36550275). In a recent study by our co-authors, *M. smithii* and *M. stadtmanae* showed broadly similar resistance to transient oxygen exposure, making it unlikely that oxygen tolerance is the main driver of the distinct cytokine responses observed here. Instead, our functional data more strongly support a mechanism based on **phagocytic uptake and subsequent intracellular RNA-TLR8 sensing.**

Our previously published data more strongly support **phagocytosis as a prerequisite** for methanogen-induced immune activation. In our previous study using human monocyte-derived dendritic cells, inhibition of uptake with cytochalasin D, together with blockade of lysosomal processing by bafilomycin A1, strongly impaired methanogen-induced activation. Consistent with this, confocal microscopy with LysoTracker provided evidence for lysosomal involvement during

archaeal uptake, and both interventions strongly reduced IL-1β release induced by *M. stadtmanae* and *M. smithii* to near-baseline levels as measured by ELISA (PLOS One, 2014; PMID: 24915454). We have now incorporated this point into the revised manuscript **(Lines 578-580)**.

Extending these findings, we additionally examined phagocytosis dependence in the current study and found that pharmacologic inhibition of phagocytosis with cytochalasin D in primary human monocytes reduced *M. smithii*-induced IL-1β and IL-6 release to near-basal levels, whereas *M. stadtmanae*-induced responses were strongly suppressed but not completely abolished, likely reflecting incomplete pharmacologic inhibition in the context of the substantially higher uptake efficiency of *M. stadtmanae* **(Supplementary Fig. 5g; newly added data, Lines 627-633).** Importantly, before inhibition, *M. stadtmanae* induced significantly higher IL-1β and IL-6 release than *M. smithii*, **whereas after inhibition this difference was no longer significant. This suggests that the stronger IL-1β and IL-6 (both belong to Class I genes) response to *M. stadtmanae* is largely dependent on more efficient uptake and subsequent intracellular sensing.** If extracellular factors such as methane production or oxygen tolerance were the dominant drivers of the species-specific difference, one would expect the difference between *M. stadtmanae* and *M. smithii* to persist even when phagocytic uptake is inhibited. Instead, this difference was lost after inhibition.

Second, our data support the RNA - TLR8 axis as the main mechanism underlying the differential responses induced by the two methanogens. **We would like to clarify that human TLR8 is not a cell-surface receptor. Rather, TLR8 is localized to endosomal compartments, where it recognizes RNA degradation products in the endosomal lumen.** Thus, archaeal material must first be taken up by the cell before it can reach TLR8. Since TLR8 recognizes ssRNA in this intracellular compartment, this also explains why blocking phagocytosis largely abolishes the response. This model is supported by both our previous and current results. In our previous study, using a TLR8-KO system, we showed that the response to *M. stadtmanae* depends critically on RNA - TLR8 signaling (Front. Immunol. 2017; PMID: 29181003). In the current manuscript, pharmacological inhibition of TLR8 markedly suppressed most archaeal-induced responses **(Fig. 5)**. To address IL-1β more directly, we additionally measured its release and found that pharmacological inhibition of TLR8 strongly reduced IL-1β secretion induced by both methanogens as well as by the TLR8 agonist TL8-506 (see figure below; these data were not included in the revised manuscript because they are informative but not central to the main

mechanistic focus of the study). This further supports that IL-1β induction in our system occurs downstream of phagocytosis-dependent TLR8 activation, rather than representing an independent upstream mechanism.

Taking together, our data suggests that the different inflammatory responses induced by the two methanogens are mainly driven by **differential uptake** and **endosomal TLR8 sensing**. Although we cannot completely exclude additional extracellular contributions, the available evidence indicates that cellular uptake and subsequent intracellular RNA sensing are the dominant mechanisms underlying the species-specific differences observed in our system.

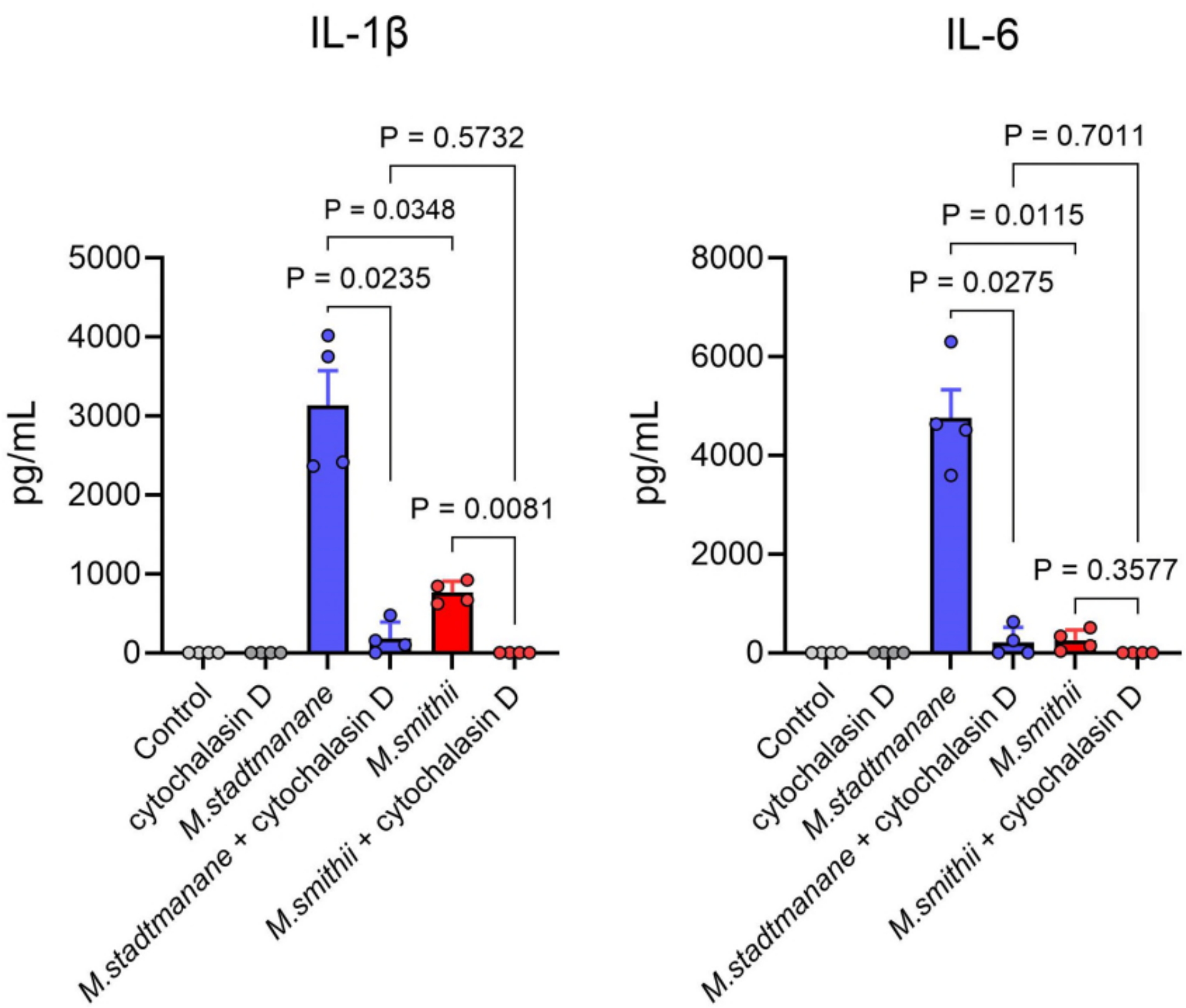

Supplementary Fig. 5g ELISA was used to quantify IL-1β and IL-6 in culture supernatants from monocytes stimulated with *M. stadtmanae* or *M. smithii* in the presence or absence of the phagocytosis inhibitor cytochalasin D. Each data point represents one donor and one independent biological batch (n = 4). Data are shown as mean ± SEM. Statistical significance was assessed

using one-way ANOVA followed by Tukey's multiple comparisons test. Exact P values are shown in the figure.

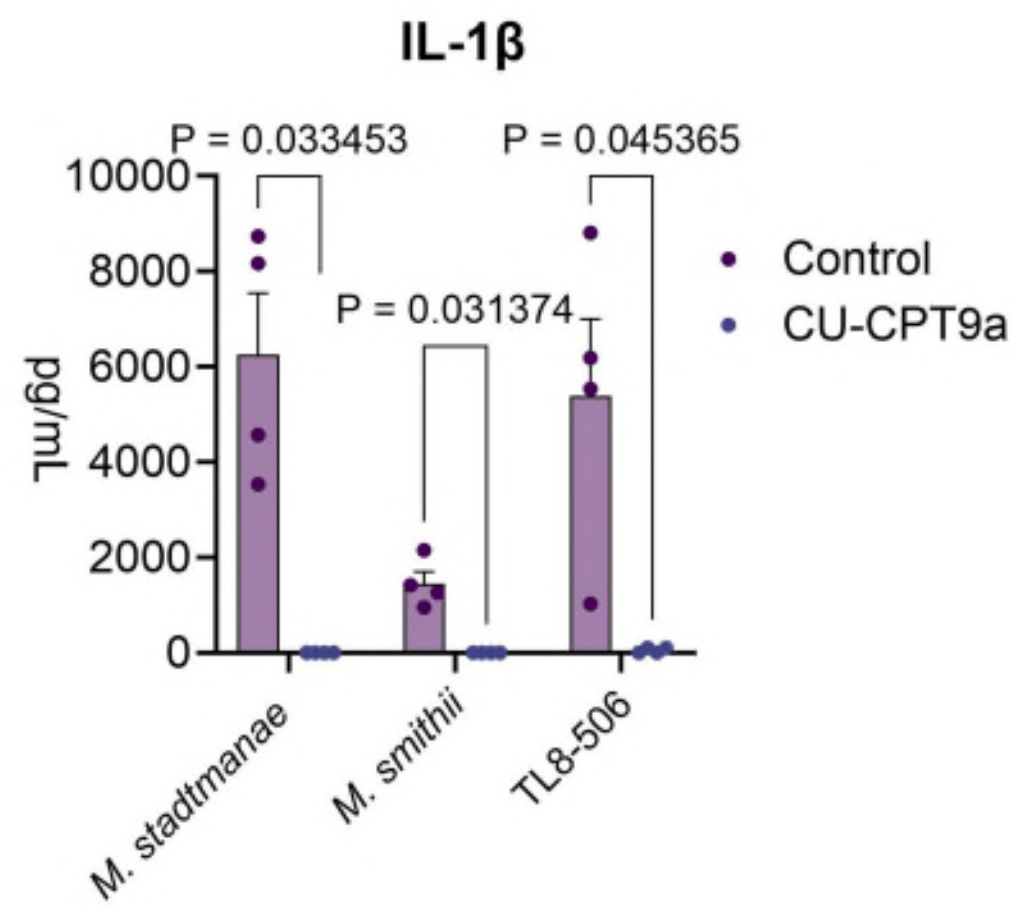

Figure: PBMCs from four independent donors were stimulated with *M. stadtmanae* or *M. smithii* in the presence or absence of the TLR8-specific inhibitor CU-CPT9a. As a positive control, PBMCs were stimulated in parallel with the TLR8-selective agonist TL8-506. IL-1β concentrations in culture supernatants were quantified by ELISA. Data are shown as mean ± SEM from 3 independent batches, each performed with cells from a different donor. Statistical analysis was performed using multiple paired t tests. Exact p values are shown in the figure.

2. Do the two archaea remain intact in the culture conditions used for stimulations? The reason why I am asking is that if they do not remain intact, and spill their intracellular contents, insights generated from the experiments are not going to have much relevance with respect to mechanisms of innate immune sensing. Another reason is that for archaeal RNA to become an innate immune ligand it has to come out of the cell. This will only happen if cells do not remain intact. Chemically, I am hard pressed to think of a difference in archaeal, bacterial and animal RNA other than 5`cap and modifications in tRNA. So basically, if author's model is correct, innate immune sensing of archaea only happens when archaeal cells are ruptured.

Response: We thank the reviewer for raising this important point. We agree that archaeal RNA must become accessible to host receptors to act as an innate immune ligand. **However, our model does not require extracellular rupture of archaeal cells in the culture medium.** Rather, both our previous and current data support that phagocytic uptake is required for methanogen-induced

immune activation **(Supplementary Fig. 5g.; newly added data)**. In this model, intact archaea are internalized and processed in endosomal or endo-lysosomal compartments, where their RNA becomes available for TLR8 sensing. This is consistent with **the subcellular localization of TLR8 as an endosomal, rather than cell-surface, RNA sensor.** Together, these findings indicate that innate immune sensing of methanogens in our system depends on uptake and intracellular processing rather than spontaneous extracellular rupture of archaeal cells.

3. The difference between the kinetics of transcriptional suppression and activation (Class I and Class II) can also be explained by differences in stabilities of the two archaeal cells in the culture conditions. This can easily be tested by qPCR analysis of archaeal RNA in cell culture supernatants. As a control, authors should spin down archaeal cells and use only the supernatants for stimulating PBMCs to rule out contributions of contaminants.

Response: We agree that extracellular instability would be an important alternative explanation for the different Class I and Class II kinetics and therefore addressed this possibility directly. First, we analyzed carefully prepared cell-free supernatants from the stimulation cultures. After removal of archaeal cells by centrifugation and 0.22 µm filtration, RNA was extracted from the supernatants, treated with DNase I, and analyzed by RT-qPCR using species-specific *rpoB* primers for *M. stadtmanae* and *M. smithii*. Under these conditions, we did not detect archaeal RNA in the cell-free supernatants, whereas the corresponding positive controls gave clear amplification signals **(Supplementary Fig. 3a; newly added data)**.

Second, we used the same processed cell-free supernatants to stimulate PBMCs and measured IL-1β, IL-6, TNF, and CCL2. These supernatants did not induce detectable cytokine production. Importantly, even when the input volume of supernatant was increased fivefold, no cytokine induction was observed **(Supplementary Fig. 3b; newly added data)**.

Together, these results argue against a relevant contribution of extracellularly released archaeal RNA or other soluble factors under our stimulation conditions. They also do not support extracellular instability as the main explanation for the distinct response kinetics. Instead, our data is more consistent with a model in which the dominant immunostimulatory pathway depends on cellular uptake and intracellular processing of archaea. We have now incorporated this point into the revised manuscript: Methods **(Lines 133 – 137; Lines 258 - 260)** and Results **(Lines 583 - 590).**

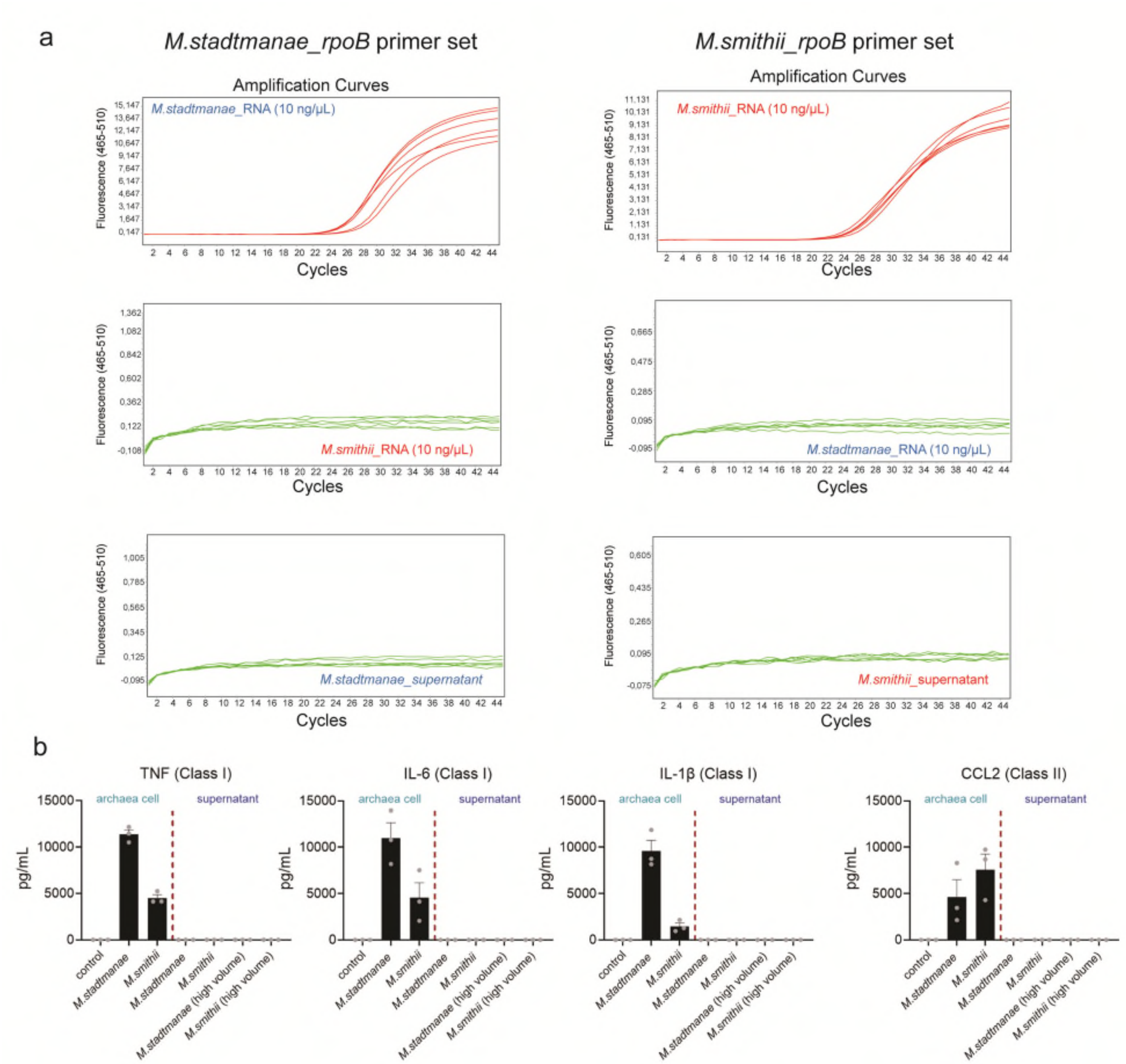

Supplementary Fig. 3 Cell-free archaeal culture supernatants do not account for PBMC activation

a Representative RT-qPCR amplification curves (x-axis: cycle number; y-axis: fluorescence intensity) obtained using two species-specific primer sets targeting rpoB. Left: *M. stadtmanae* rpoB primers; right: *M. smithii* rpoB primers. Amplification is shown for purified archaeal standard RNA (10 ng/μL) and for rigorously processed archaeal culture supernatants. The experiment was performed in three independent batches, each with two technical replicates per condition, each amplification curve represents one replicate. **b** ELISA quantification of cytokines/chemokines in PBMCs culture supernatants following stimulation with intact archaeal cells or corresponding cell-

free supernatants. Supernatants were tested at the standard working volume and at high volume (5× the working volume). Data are shown as mean ± SEM from 3 independent batches, each performed with cells from a different donor.

4. This could be an alternative mechanism more in agreement with the author's proposed model. It is possible that RNA from the two archaea is differentially modified. What are those modifications?

Response: We agree that differential RNA modifications could, in principle, influence TLR8 activation. Archaeal RNAs, especially tRNAs and rRNAs, are known to carry numerous post-transcriptional modifications. However, we did not perform a comprehensive analysis of RNA modification patterns in *M. stadtmanae* and *M. smithii* in this study and therefore cannot specify which individual modifications differ between the two species.

However, to this end, we directly tested whether RNA from the two archaea differs in intrinsic immunostimulatory activity when delivered at identical doses. As shown in Fig. 6a and Fig. 6b (newly added data), transfection of equal amounts of purified *M. stadtmanae* and *M. smithii* RNA induced indistinguishable Class I and Class II expression programs. If RNA modifications do contribute, they would more likely do so by affecting RNA processing in endosomal compartments. However, in our standardized RNA delivery experiments, both archaeal RNAs elicited comparable responses.

Taken together, these findings argue against RNA sequence or modification differences as the main explanation for the divergent kinetics observed with intact cells and instead support a model in which differences in uptake and endosomal RNA delivery determine the downstream response. We have now incorporated this point into the revised manuscript: Results **(Lines 769 - 774).**

5. What is the effect of phagocytosis inhibitors on innate immune stimulation by the two archaea? As a control, authors should spin down archaeal cells and use only the supernatants in combination with beads to rule out the contribution of contaminants to differences in phagocytosis.

Response: In our previous study (PLOS ONE; PMID: 24915454), we showed that *M. stadtmanae* and *M. smithii*-induced activation depends on phagocytosis and endosomal processing, as inhibition of actin-dependent uptake with cytochalasin D or interference with endosomal maturation and acidification using bafilomycin A1 strongly reduced TNF and IL-1β release, whereas LPS-induced cytokine production was unaffected. We confirmed this again in the present study, where cytochalasin D strongly suppressed IL-1β and IL-6 responses induced by both *M.*

stadtmanae and *M. smithii*, further supporting that phagocytosis is required for innate immune stimulation by both archaea **(Supplementary Fig. 5 newly added data)**.

We also thank the reviewer for the important suggestion to control for a possible contribution of soluble contaminants to the observed differences in phagocytosis. As noted above in our response to comment 3, we had already tested cell-free archaeal supernatants in stimulation assays and found that they did not induce detectable cytokine responses, arguing against the presence of soluble immunostimulatory material at levels sufficient to explain the observed effects.

To address this point more directly **in the context of phagocytosis**, we additionally performed the suggested bead-based control experiment. Cell-free supernatants were incubated together with 0.5 µm fluorescent carboxylate-modified polystyrene latex beads, followed by flow-cytometric analysis of bead uptake by human monocytes. This assay was adapted from a published method for quantifying phagocytosis by human monocytes (Cytometry A., 2014; PMID: 24132941). Across three independent experiments using cells from three donors, we did not observe a clear difference in bead uptake between conditions (see figure below; these data were not included in the revised manuscript because they are informative but not central to the main mechanistic focus of the study.). These results argue against soluble contaminants in archaeal supernatants as the cause of the differential uptake observed with intact archaea.

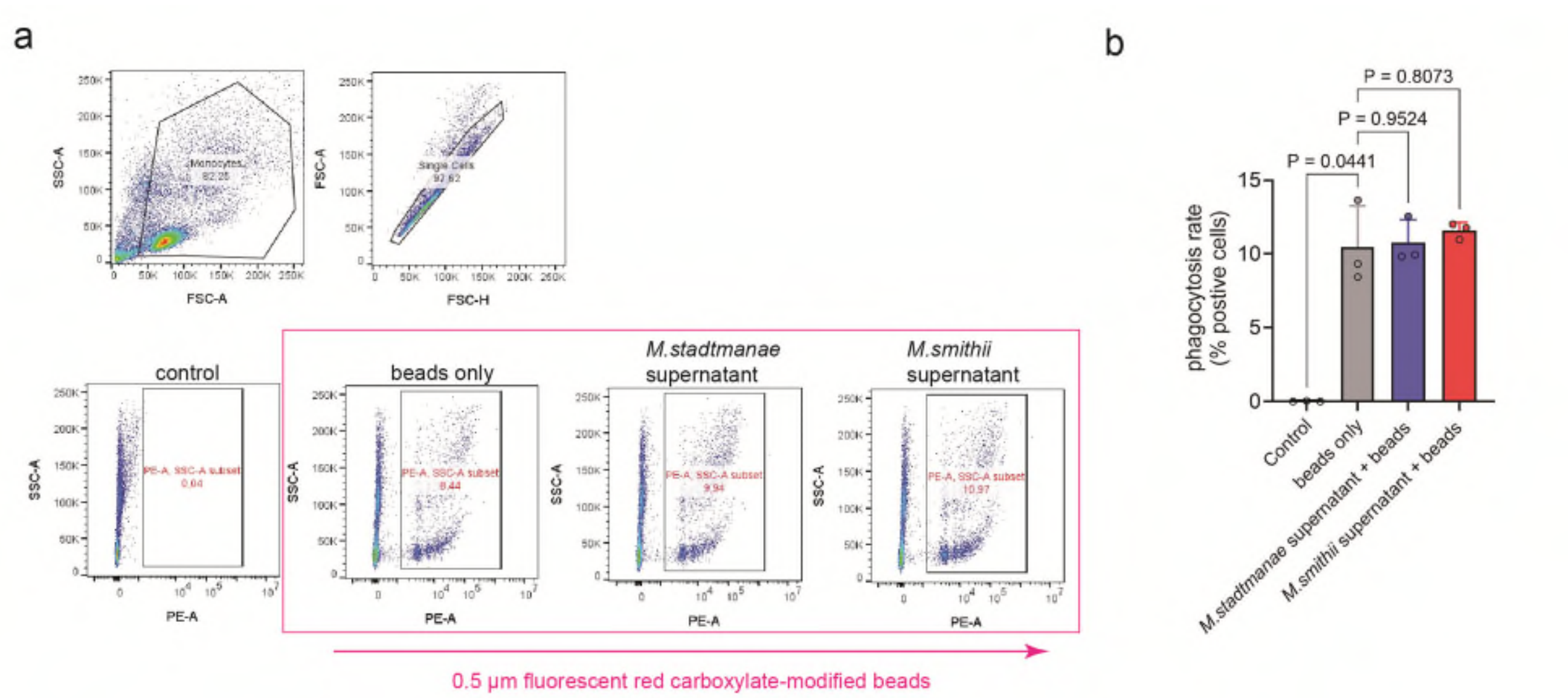

Figure. **a** Representative flow-cytometry gating strategy for uptake of 0.5 µm fluorescent carboxylate-modified polystyrene latex beads by human monocytes. Upper panel, monocyte gating strategy. Lower panel, identification of bead-positive cells within the monocyte gate. **b** Quantification of bead-positive monocytes in the presence of the indicated cell-free archaeal

supernatants. Data from three independent experiments using three donors are shown, with each dot representing one donor. Bars indicate mean ± SD. Statistical significance was assessed by one-way ANOVA followed by Dunnett's multiple-comparisons test against the beads-only control. Exact p values are shown in the figure.

6. Lines 671-674: I am not sure there is evidence to support this statement. It may be true, but there is no evidence at this point given that TLR8 inhibitor + agonist combination did suppress inflammatory gene expression. Lines 681-683 are more reasonable.

Response: We agree with the reviewer that the original wording was too strong. Based on the inhibitor data alone, we could not conclude that residual gene expression was still TLR8-dependent. We have therefore revised the manuscript accordingly and removed the overinterpretation from the Results section **(Lines 715-748)**.

To address this point more directly, we added experiments using the BLaER1 monocyte model together with the corresponding TLR8 knockout line. BLaER1 cells are a well-established model for studying innate immune signaling in human monocytes and can be transdifferentiated into monocyte-like cells, as reflected by loss of the B cell marker CD19 and acquisition of the monocyte-associated markers CD11b and CD14 (Methods Mol Biol. 2018; PMID: 29177855). The TLR8 knockout BLaER1 line used here was established and functionally validated in our previous study on archaeal sensing (Front. Immunol. 2017; PMID: 29181003), and the relevant procedures are now described more clearly in the Methods **(Lines 153-160; Lines 308-312)**. Using this system, we then focused on two categories of *M. stadtmanae*-induced genes defined by their response to CU-CPT9a. One category comprised genes whose expression was markedly reduced by the inhibitor but remained above the DEG cutoff because of their strong induction. The second category comprised genes that were essentially unchanged by CU-CPT9a **(Fig. 5c; newly added data)**. As representative examples, we selected ***IL6*** from the first group and ***SLC7A11***, a representative NRF2-associated redox-response gene, from the inhibitor-insensitive group.

To further confirm the TLR8 knockout phenotype in the present study, we stimulated control and TLR8 KO cells with the synthetic TLR8 agonist TL8-506 and with LPS. As expected, TLR8 KO cells failed to respond to TL8-506, whereas responsiveness to LPS was preserved, confirming selective loss of TLR8 signaling **(Fig. 5d; newly added data)**. Using this system, we then examined representative genes from the two CU-CPT9a-defined categories under *M. stadtmanae* stimulation. *IL6* was strongly reduced by CU-CPT9a, but because its induction was very high,

expression still remained above the DEG cutoff in the inhibitor-treated condition. In TLR8 KO cells, however, *IL6* expression was abolished and returned to basal levels, showing that residual expression after inhibitor treatment does not indicate TLR8 independence **(Fig. 5d; newly added data)**. In contrast, *SLC7A11* was unaffected by CU-CPT9a and likewise remained unchanged in TLR8 KO cells **(Fig. 5d; newly added data)**. Together, these results indicate that the CU-CPT9a-insensitive gene set is genuinely TLR8-independent, whereas residual expression after inhibitor treatment, particularly for strongly induced genes such as *IL6*, does not exclude full TLR8 dependence.

To further characterize the genes that remained upregulated in the presence of CU-CPT9a, we analyzed the 107 transcripts that were still induced under inhibitor treatment. Interaction network analysis showed that a subset of these genes formed three connected clusters, including a CD276/IL3RA-centered cluster, a redox-associated cluster organized around the SLC7A11-GCLM axis, and an immediate-early signaling cluster featuring DUSP1 **(Fig. 5e; newly added data)**. To complement this network-based analysis, we also performed Reactome pathway enrichment analysis on these 107 genes. This revealed strong enrichment of NFE2L2-related signaling, with the top pathways including “NFE2L2 regulating antioxidant/detoxification enzymes” and the “KEAP1-NFE2L2 pathway” **(Supplementary Fig. 8g; newly added data)**, which is consistent with the redox-associated cluster identified in the interaction network.

We then revised the description of Fig. 5 in both the Results **(Lines 715-748)** and Discussion **(Lines 917 - 933)** sections to reflect more accurately that the response contains both TLR8-dependent and TLR8-independent components.

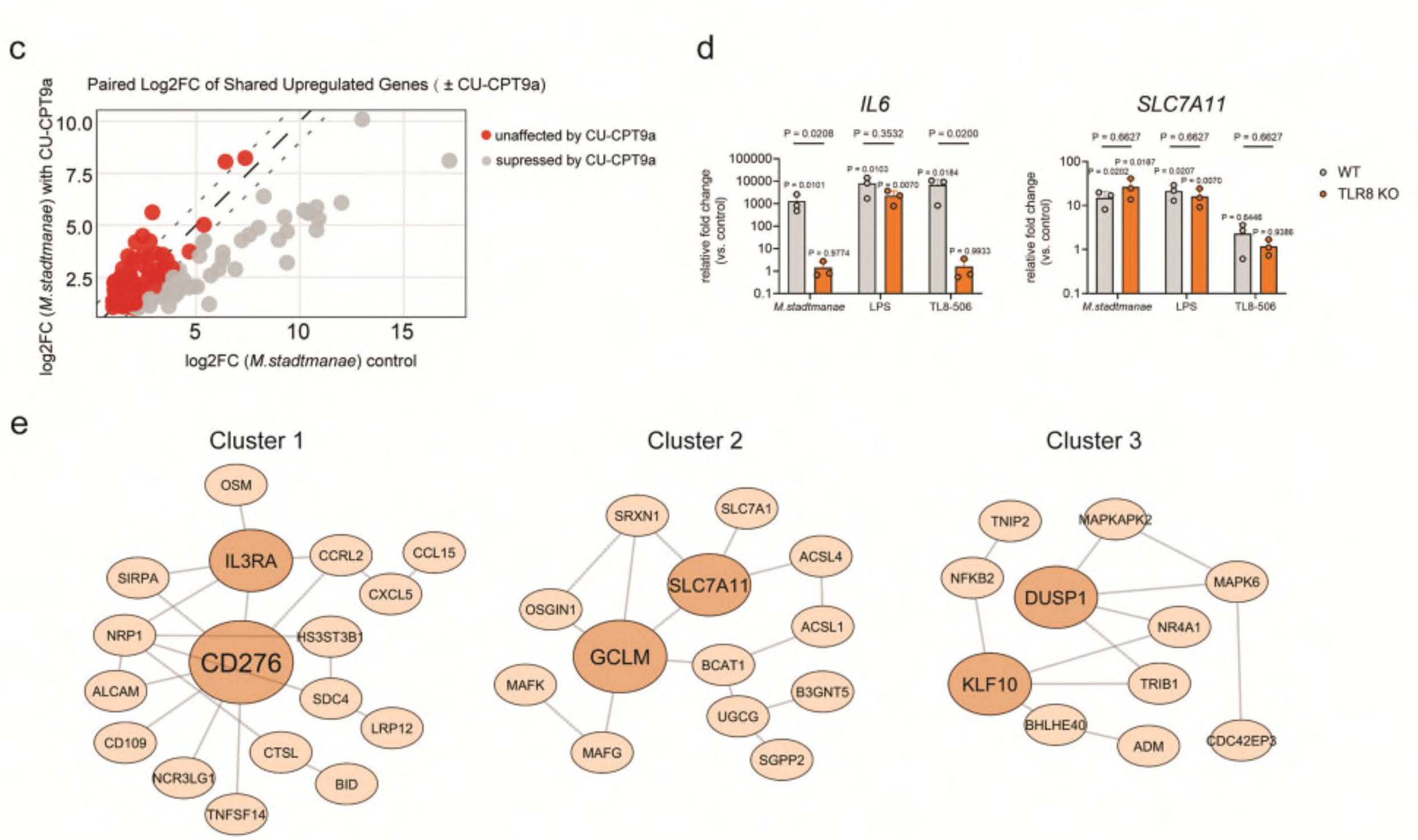

Fig. 5 c Dot plot showing paired log2FC values of 171 shared upregulated genes. Each dot represents one DEG detected under both *M. stadtmanae* stimulation alone and *M. stadtmanae* stimulation in the presence of CU-CPT9a. The x-axis shows the log2FC under *M. stadtmanae* stimulation alone, and the y-axis shows the corresponding log2FC in the presence of CU-CPT9a. The long dashed diagonal line (y = x) indicates equal expression between conditions. Two additional short dashed lines, drawn parallel to the diagonal at ±1 log2FC difference, define two response categories based on CU-CPT9a sensitivity, where Δlog2FC is defined as log2FC in the absence minus log2FC in the presence of CU-CPT9a: (1) gray dots, 64 genes markedly reduced by CU-CPT9a (Δlog2FC ≥ 1); and (2) red dots, 107 genes largely unaffected by CU-CPT9a (Δlog2FC < 1). **d** RT-qPCR analysis of *IL6* and *SLC7A11* expression in WT and TLR8 KO BLaER1 cells following stimulation as indicated. TL8-506 and LPS were included to confirm selective loss of TLR8 signaling in TLR8 KO cells. Data are shown as mean ± SD from three independent batches. Statistical significance was assessed by one-way ANOVA with Dunnett's multiple comparisons test for comparisons of each stimulated group with its corresponding unstimulated control within the same genotype. These p values are indicated above the bars. Comparisons between WT and TLR8 KO cells under each stimulation condition were performed using multiple t-tests, and the corresponding p values are indicated above the connecting lines. **e** Interaction network analysis of the 107 genes that remained upregulated in the presence of CU-CPT9a. High-confidence

interactions (STRING score ≥ 0.9) were retrieved from STRING and visualized in Cytoscape, revealing three connected clusters.

7. TLR8 is facing outside the cell. Why do the authors need to transfect RNA to engage TLR8? Cytosolic delivery of RNA should engage other innate immune sensors. What is the effect of just stimulating cells with archaeal RNA? Phagocytosis and rupture of archaeal cells would release RNA which can be sensed by TLR8 in endosomes. Transfection of RNA with DOTAP does not capture this process. Just adding archaeal RNA to cells and incubating them would lead to activation of TLR8, although this would require large amounts of RNA.

Response: We would like to clarify that human TLR8 is not a cell-surface receptor. Rather, TLR8 is localized to endosomal compartments, where it recognizes RNA degradation products in the endosomal lumen. Thus, RNA must first gain access to this intracellular compartment in order to engage TLR8 efficiently. Since extracellular RNA does not efficiently reach the endosomal lumen in a controlled way, RNA is commonly complexed with a cationic carrier such as DOTAP to promote cellular uptake and endosomal access. This allows direct testing of the TLR8-dependent activity of RNA itself under standardized conditions and avoids confounding by species-specific differences in uptake of intact archaeal particles. This approach is widely used in the TLR7/8 field for mechanistic interrogation of endosomal RNA sensing (Science, 2004; PMID: 14976262; PLoS One, 2015; PMID: 25785446; J Immunol., 2014; PMID: 24813206).

To address the reviewer's point more directly, we also tested archaeal RNA in the absence of any delivery reagent. Under our experimental conditions, naked archaeal RNA at 1 μg/mL did not induce detectable production of IL-6, IL-1β, TNF, or CCL2 **(Supplementary Fig. 9c, newly added data)**. In contrast, the same RNA reproducibly induced these cytokines when complexed with DOTAP, indicating that efficient intracellular delivery is required for robust activation in our system.

To address whether DOTAP-mediated RNA delivery altered the sensing pathway, we stimulated primary monocytes with DOTAP-complexed archaeal RNA in the presence or absence of the selective TLR8 antagonist CU-CPT9a. CU-CPT9a reduced the response to baseline and rendered IL-6, IL-1β, TNF, and CCL2 undetectable in all three donors **(Supplementary Fig. 9c, newly added)**, indicating that the measurable cytokine response under these conditions is largely dependent on endosomal TLR8.

Taken together, these results show that naked archaeal RNA is not sufficient to trigger a robust response under our experimental conditions, whereas DOTAP-mediated delivery provides access to the endosomal TLR8 pathway and allows direct testing of archaeal RNA as a TLR8 ligand. We have now incorporated this point into the revised manuscript: Results **(Lines 761-768).**

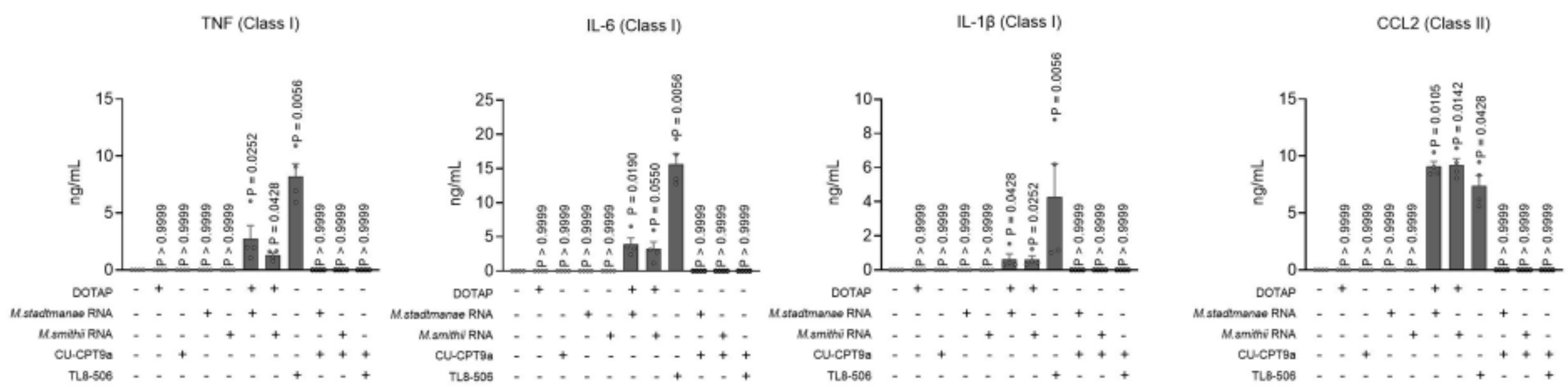

Supplementary Fig. 9 c Primary monocytes were stimulated for 24 h with archaeal total RNA (1 μg/mL), either without a delivery reagent (naked RNA) or complexed with DOTAP, in the presence or absence of the selective TLR8 antagonist CU-CPT9a. Cell-free supernatants were collected and TNF, IL-6, IL-1β, and CCL2 were quantified by ELISA. Data are shown as individual donors with mean ± SEM (n = 4 donors from 3 independent batches). Statistical significance was assessed using the Friedman test followed by Dunn's multiple comparisons test, with each condition compared against the unstimulated control. Exact p values are shown in the figure.

8. Why are authors using DOTAP for RNA transfections? In this reviewer's experience, DOTAP induces significant toxicity in myeloid cells. It is recommended to use reagents that are optimized for RNA transfections.

Response: We chose DOTAP intentionally because our goal was not generic cytosolic RNA transfection, but efficient delivery of RNA into the endosomal compartment, where human TLR8 senses RNA. In the original study establishing human TLR8 recognition of ssRNA, RNA ligands were likewise complexed with DOTAP to enable uptake and endosomal sensing (Science, 2004; PMID: 14976262). We also used the same approach in our previous study on archaeal RNA sensing in human myeloid cells (Front Immunol., 2017; PMID: 29181003).

We agree with the reviewer that cationic lipids, including DOTAP, can be toxic in myeloid cells depending on dose and exposure time. For this reason, all experiments included a DOTAP-only control, which did not induce detectable cytokine production or other activation readouts compared with untreated cells. In addition, we directly assessed viability in primary human $CD14^+$ monocytes

by PI staining and flow cytometry. At the working concentration used in our study (10 µg/mL), DOTAP did not increase the proportion of PI-positive cells compared with the no-DOTAP control across three donors, even after 24 h exposure. By contrast, a 10-fold higher concentration (100 µg/mL) caused near-complete loss of viability, confirming that the assay readily detected toxicity when present.

Together, these data indicate that the DOTAP concentration used in our RNA delivery experiments does not cause detectable cytotoxicity in primary monocytes and is therefore unlikely to confound the conclusions drawn from these assays. We have now stated the DOTAP working concentration explicitly in the Methods and added the PI-based viability control, including gating and donor-resolved data, as **Supplementary Fig. 9a, b; newly added data, and Lines 756-761 in Results.**

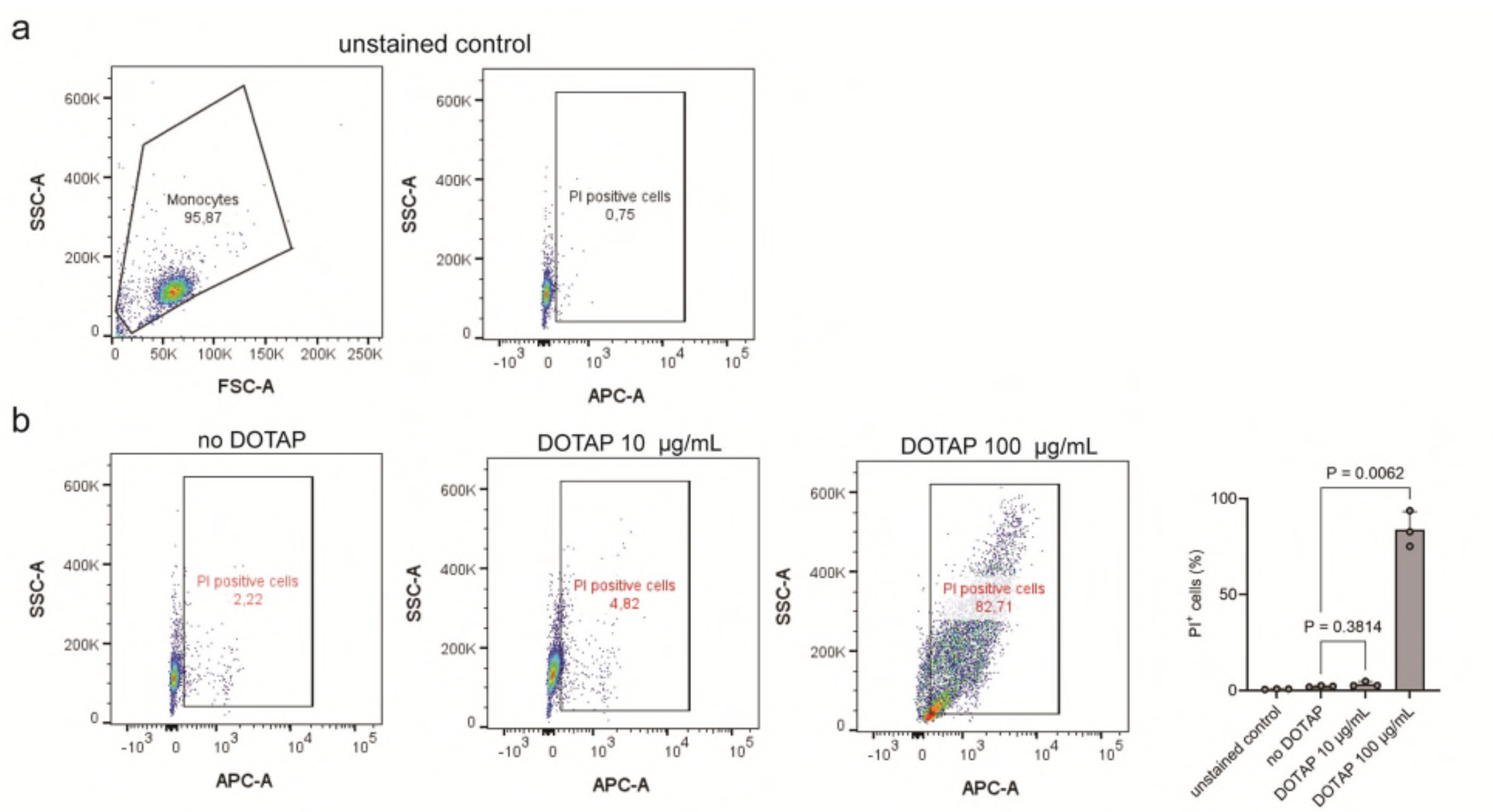

Supplementary Fig. 9 a Gating strategy for flow-cytometric analysis of PI staining in primary human PBMCs derived monocytes. Cells were first gated on single cells and then on monocytes, followed by assessment of PI fluorescence in the APC channel to define the PI + population. **b** left panel: representative flow-cytometry dot plots of PI staining in primary human PBMCs monocytes, including an unstained control to define background fluorescence and set the PI+ gate, a no-DOTAP control, DOTAP at the working concentration (10 µg/mL), and a 10× DOTAP (100 µg/mL) condition as a positive toxicity control. Right panel: Quantification of PI^+ cells across three independent donors shown as mean ± SD (n = 3). Statistical analysis was performed on donor-

matched (paired) samples using repeated-measures one-way ANOVA followed by Dunnett's multiple comparisons test. Exact p values are shown in the figure.

Minor points:

1. Lines 80-82: There are some reports of viral proteins being detected by TLR2.

Response: We thank the reviewer for this important point. We agree that viral sensing is not exclusively mediated by nucleic acids, as several studies have shown that viral proteins can also engage TLR2. We have therefore revised the sentence to avoid overly absolute wording **(Line 68 - 69).**

2. TNF-α is now named TNF.

Response: We thank the reviewer for this correction. We have revised the text throughout the manuscript and now use TNF instead of TNF-α.

Reviewer #2 (Remarks to the Author)

SUMMARY

The manuscript by Xu et al. examines differential transcriptomic responses of PBMCs and monocytes to two species of human commensal methane-producing archaea. The main observation in the first portion of study are that M. smithii and M. stadtmanae induce overlapping but distinct gene expression programs. Specifically, M. stadtmanae induces stronger expression of many immune response genes at 4 hours compared to

M. smithii. By 24 hours, the transcriptomes have largely converged. The remainder of the manuscript examines the mechanisms of these differential gene expression responses. The authors show that TLR8 is required for almost all gene expression responses to both species M. stadtmanae is taken up by monocytes more efficiently than M.smithii Stimulation with purified archaeal RNA from the two species produces the same gene expression responses, indicating that the differences require whole-cell archaea. Thus, it appears that the two archaea species signal through identical mechanisms and the difference in transcriptomic response can be fully explained by the slower and overall lower phagocytic uptake of M. smithii. M. stadtmanae activates NFkB more efficiently, leading to greater p65 occupancy at Class I genes. In contrast, STAT1/2 occupancy at target genes is comparable between the two species. This is likely explained by the fact that TLR8 activates NFkB and Type I IFN / ISGF3 gene expression programs with different dose-sensitivity. The NFkB pathway has a higher dynamic range, whereas ISGF3-gene expression responses saturate at a lower dose Overall, this is a well-executed study with notable strengths:

The main dataset is derived from a fairly large number of donors

(n=9), and reproducibility is impressively high between the donors

The timecourse measurements, especially in Figure 3, are informative

The proposed mechanism is interesting and has broader implications for

our understanding of TLR8 signaling

Response: Thank you for this thoughtful and generous assessment of our manuscript. We are pleased that the reviewer finds the study well executed and the proposed mechanism compelling.

MAIN AREAS FOR IMPROVEMENT:

If one of the goals of the study is to elucidate differences between M. smithii and M. stadtmanae (as suggested by the title and abstract), then more attention needs to be paid to the differences that would explain differential TLR8 engagement (since the data indicate that equivalent activation of TLR8 using archaeal RNA produces the same transcriptomic responses). For example:

- The Introduction should provide more background on the differences between the two species

- Why is M. stadtmanae taken up by monocytes more efficiently? What is known in the literature? If this is not known, can the authors identify proteins (or genes) that are more highly expressed in one species than another?

Response: We thank the reviewer for this important point. We agree that, because the manuscript focuses on differential responses to *M. stadtmanae* and *M. smithii*, the revised version should provide clearer biological context for these two methanogens and better connect species-level differences to differential TLR8 engagement.

We therefore expanded the Introduction to include additional background on these organisms. In addition to previously described differences in abundance and disease associations, we now also highlight that *M. stadtmanae* and *M. smithii* share the same ecological niche but differ in fundamental biology that may influence host interaction, including methanogenic metabolism, genome characteristics, and predicted adhesin-like surface protein repertoires and domain architecture. Together, these published observations support the idea that the two methanogens are not equivalent from the perspective of host cell interaction **(Lines 93-100).**

In the revised manuscript, we further strengthened the evidence for differential uptake by adding additional validation experiments, including archaeal **antibody-based analyses**, which again supported that *M. stadtmanae* is internalized more efficiently than *M. smithii* by human monocytes **(Fig. 3a, newly added data)**. This is an important part of the story in the present study, because our main focus here is to show that differences in uptake of intact archaea lead to different levels of TLR8 engagement and thereby shape the downstream transcriptional response.

We also thank the reviewer for raising the question of what causes this uptake difference. We agree that this is an interesting and important direction. At the same time, this process is likely influenced not only by archaeal features, but also by host-cell factors involved in recognition, phagocytic uptake, and intracellular handling. **At present, there is still limited evidence to explain this mechanism**. The available data support that the two species behave differently in their interaction with host cells, but resolving the basis of this difference will require much more systematic mechanistic work. This is something beyond the scope of this manuscript, but we are currently pursuing **follow-up studies**.

To further probe the basis of the strain difference, we performed an exploratory bead-uptake assay. We considered two possible explanations for the stronger uptake observed with *M. stadtmanae*: either *M. smithii* generally suppresses phagocytosis, or *M. stadtmanae* generally enhances it. To

distinguish between these possibilities in a simple experimental setting, cells were pre-exposed to each archaeal species and then challenged with phagocytosable beads. Under this logic, prior exposure to *M. smithii* would be expected to reduce bead uptake if it broadly inhibits phagocytosis, whereas prior exposure to *M. stadtmanae* would be expected to increase bead uptake if it broadly promotes phagocytosis. Primary human CD14^{+} monocytes were preincubated with *M. stadtmanae* or *M. smithii* for 2 h, washed to remove extracellular archaea, and then exposed to fluorescent phagocytosable beads. Under these conditions, prior exposure to *M. smithii* had little effect on subsequent bead uptake, arguing against a broad suppression of phagocytosis by *M. smithii*. In contrast, prior exposure to *M. stadtmanae* reduced subsequent bead uptake, which does not support a simple model in which *M. stadtmanae* generally enhances phagocytic activity. Rather, this result is more consistent with the idea that *M. stadtmanae* itself is taken up more efficiently and that this earlier uptake affects the subsequent handling of a second particulate cargo.

At the same time, we interpret this experiment cautiously. The bead assay helps to argue against two possibilities raised by the reviewer, but it does not identify the underlying uptake mechanism or explain the strain-specific difference at the molecular level. Because these data are exploratory and do not yet provide mechanistic resolution, we have not included them in the revised manuscript. Instead, we now acknowledge this point more explicitly as an important open question and a **clear direction for future work.**

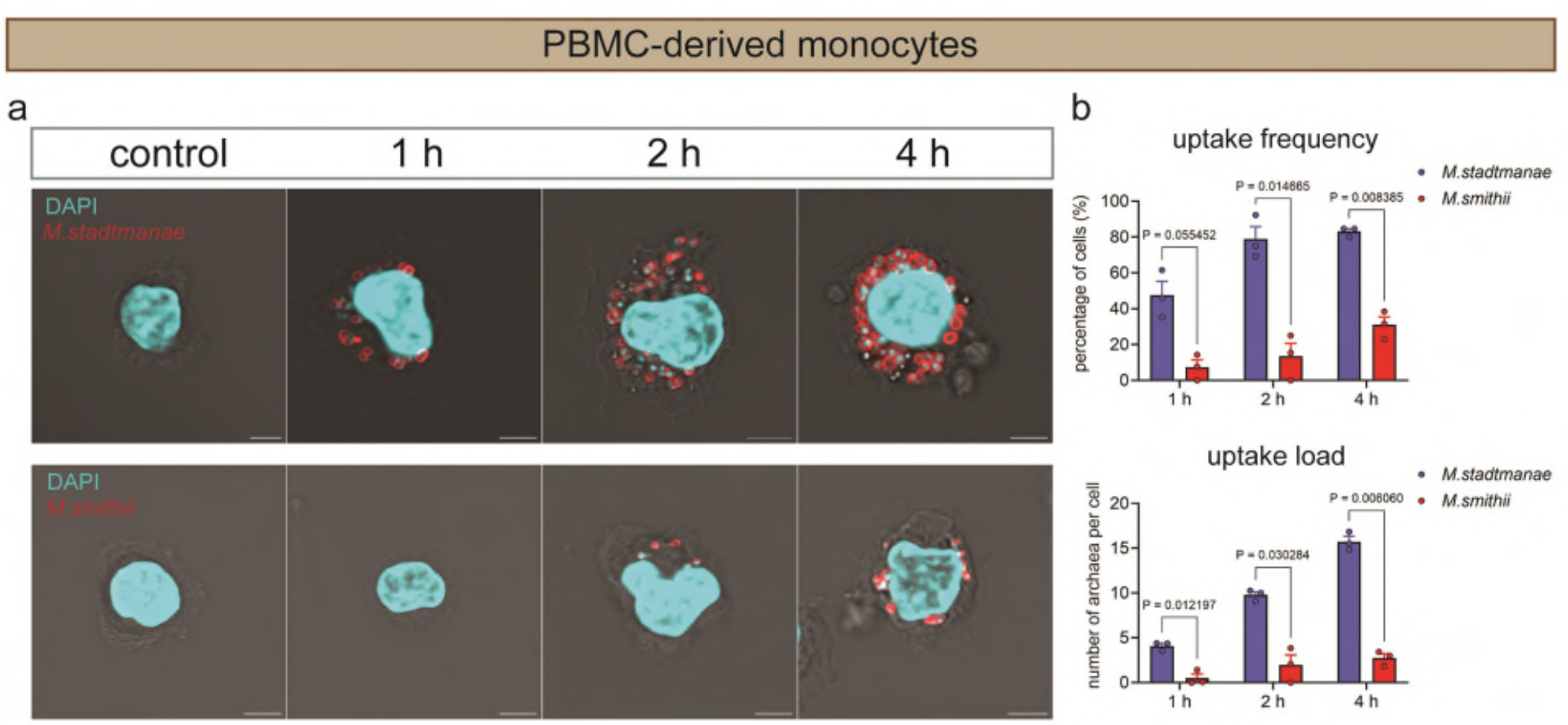

Fig. 3. a Representative merged confocal images of primary monocytes stimulated with *M. stadtmanae* or *M. smithii* for 1, 2, or 4 h, as indicated. Archaea were visualized by species-specific

antibody staining and appear as ring-like structures (red). Nuclei were counterstained with DAPI (cyan). Phase-contrast images are included to show cell morphology and outline. Images were acquired with a 63× oil immersion objective. Displayed images represent enlarged views of selected regions to better visualize archaea-associated signals. Scale bar = 5 µm. **b** Quantification of archaeal uptake in primary monocytes after stimulation with *M. stadtmanae* or *M. smithii* for 1, 2, or 4 h. Upper panel, uptake frequency, defined as the percentage of cells containing at least one internalized archaeal cell. Lower panel, uptake load, defined as the mean number of internalized archaea per cell, with each ring-like structure scored as one archaeal cell. For each condition, 30-50 cells were analyzed from 3 independent donors. For each donor, data were first summarized across the analyzed cells. In the upper panel, each dot represents the uptake frequency for one donor, and in the lower panel, each dot represents the mean uptake load for one donor. Data are shown as mean ± SEM. Statistical significance was assessed using a two-tailed paired test. P values are indicated in the figure.

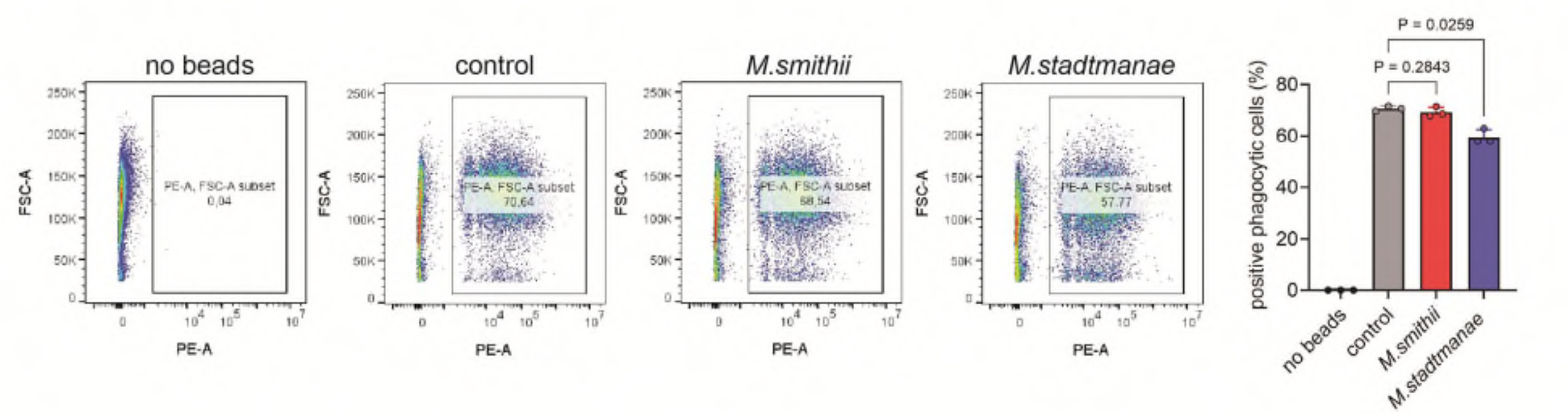

Figure Left panel: Representative flow-cytometry plots showing uptake of fluorescent beads measured in the PE channel in primary human CD14$^+$ monocytes. Monocytes were either left untreated or preincubated with *M. stadtmanae* or *M. smithii* for 2 h, followed by washing to remove extracellular archaea and incubation with beads for an additional 1 h. A no-bead control is included to define background fluorescence and set the gate. Right panel: Quantification of bead uptake across three independent donors. Each dot represents one individual donor from one independent batch (n = 3). Data are shown as mean ± SD. Statistical significance was determined using one-way ANOVA. Exact P values are indicated in the panel.

- The entire study is done using a fixed dose of archaea (10^7 per ml). Can the gene expression differences between the two species be overcome by increasing the amount of M. smithii?

Response: To address whether the difference between *M. smithii* and *M. stadtmanae* can be reduced by increasing the amount of *M. smithii*, we performed an additional dose-escalation experiment in primary human PBMCs from three independent donors (see Figure blow). Cells were stimulated with *M. stadtmanae* or *M. smithii* at 10^7 or 10^8 cells/mL, and cytokine/chemokine release in the supernatant was measured as representative Class I and Class II readouts (Class I: TNF, IL-6, IL-1β; Class II: CCL2).

The effects of the higher dose were not uniform. For *M. stadtmanae*, increasing the dose from 10^7 to 10^8 cells/mL led to only limited further changes, with slight increases in TNF and IL-1β, but lower IL-6 and CCL2 at the higher dose. For *M. smithii*, TNF increased clearly, IL-6 and IL-1β increased more modestly, and CCL2 decreased slightly.

These data show that increasing the amount of *M. smithii* can reduce the difference between the two species, particularly for the Class I readouts, because the relative increase was more pronounced for *M. smithii*, whereas *M. stadtmanae* showed only minor further increases or even slight reductions at the higher dose. At the same time, even at 10^8 cells/mL, *M. smithii* remained overall lower than *M. stadtmanae* at 10^7 cells/mL. Thus, increasing the amount of *M. smithii* can narrow the gap, but does not fully overcome the species-dependent difference.

We therefore consider 10^7 cells/mL to be a suitable working concentration for the main study, since differences are clearly detectable at this dose. The limited additional increase observed at higher input, particularly for *M. stadtmanae*, may suggest that uptake and/or downstream processing in monocytes are not simply proportional to particle number and may already approach a functional limit under these conditions. Because this experiment was performed to address a reviewer's specific question and does not form part of the main mechanistic line of the present study, we did not include these additional data in the revised manuscript.

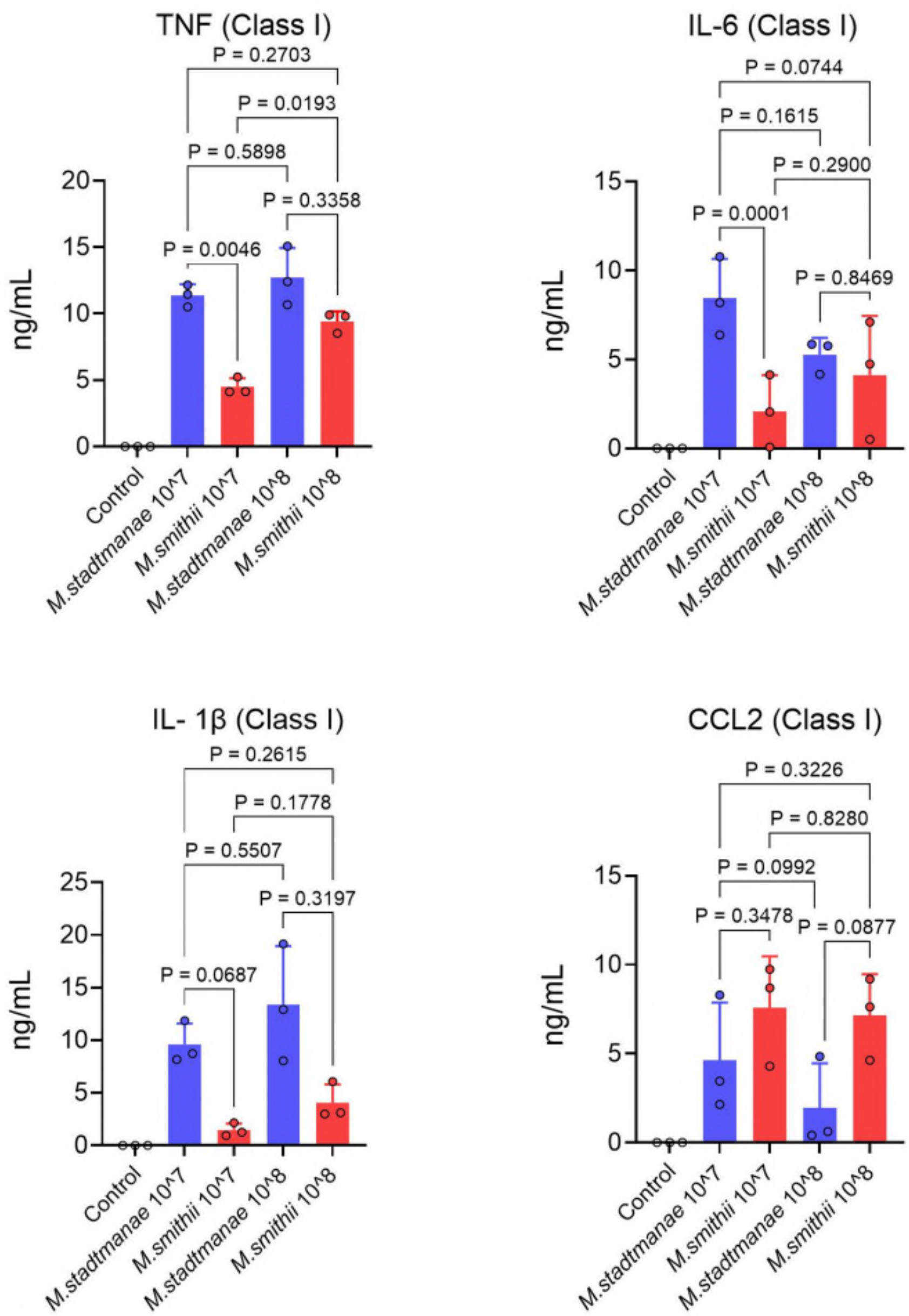

Figure. Primary human PBMCs were stimulated with *M. smithii* or *M. stadtmanae* at 10^7 or 10^8 cells/mL for the indicated time, and cytokines/chemokines in culture supernatants were quantified by ELISA. Each symbol represents one donor, with each donor corresponding to an independent batch (n=3). Bars indicate mean ± SD. Exact P values are shown in the figure.

The proposed mechanism is that weak engagement of TLR8 is sufficient to strongly induce ISGF3 target genes but not NFkB target genes. This is a very interesting finding that would be generalizable to all TLR8-activating ligands and warrants additional investigation.

- Does stimulation with different doses of a synthetic TLR8 ligand recapitulate the gene expression differences seen with the two archaea species? If the hypothesis is correct, low dose TLR8 agonist should preferentially induce Class II genes.

Response: We agree that this is an important question. If the difference between the two archaea simply reflected weaker versus stronger TLR8 stimulation, then one might indeed expect that low-dose stimulation with a synthetic TLR8 agonist would preferentially induce Class II genes. We therefore tested this directly in our system by performing a dose titration of the synthetic TLR8 agonist TL8-506 in primary human monocytes and measuring representative Class I and Class II readouts. **Under these conditions, low-dose TL8-506 did not show a clear preference for Class II induction.** Instead, both response classes were induced across the tested dose range, and the archaeal pattern was not reproduced in a simple way **(Supplementary Fig. 10; newly added data)**.

However, we do not think this is unexpected, because archaeal RNA and synthetic small-molecule agonists do not activate TLR8 in the same way. For human TLR8, RNA-driven activation is thought to involve a multistep process in which RNA must first be degraded into the actual ligands that bind the receptor. Structural and functional studies showed that optimal activation requires two ligand-binding pockets, with a uridine nucleoside in site 1 and a short oligoribonucleotide fragment in site 2 (Nat Struct Mol Biol., 2015; PMID: 25599397). Consistent with this, endolysosomal RNases upstream of TLR8, including RNase T2 and RNase 2, have been implicated in generating the relevant RNA-derived ligands (Cell., 2019; PMID: 31778653; Immunity., 2020; PMID: 32294405). Thus, for RNA, the effective TLR8 signal is shaped not only by RNA input, but also by endosomal uptake and trafficking, as well as by the timing and extent of ligand generation during RNA processing. By contrast, several commonly used synthetic TLR8 agonists, including the imidazoquinolines R848, do not reproduce the RNA-driven two-pocket mode of receptor engagement (Nat Chem Biol., 2018; PMID: 29155428), and TL8-506 has likewise been described as a synthetic TLR8 agonist that binds site 1 (Cell., 2019; PMID: 31778653). This is important for dose-response interpretation, because small molecules engage the receptor through a different structural and kinetic route and do not depend on the same RNase-dependent processing steps that shape RNA-mediated activation.

We also considered using a synthetic RNA oligonucleotide. However, this would not fully solve the problem either. In practice, it is difficult to identify an RNA reagent that is both clearly TLR8-selective and still reflects the archaeal particle setting. For RNA, the upstream steps matter greatly,

including endolysosomal delivery, acidification, and RNase-dependent processing. Different RNA substrates can be handled differently and may generate different degradation products with different kinetics. Therefore, even if an RNA oligonucleotide activates TLR8, it would not necessarily recreate the same processing dynamics that we think are important for the archaeal response. For this reason, we used a TLR8-selective agonist for the dose experiment and relied on TLR8-specific inhibition in our system to support pathway specificity.

These data have been added to the revised manuscript **(Results, Lines 782-790)** and are discussed in the revised **Discussion (Lines 939-954)**.

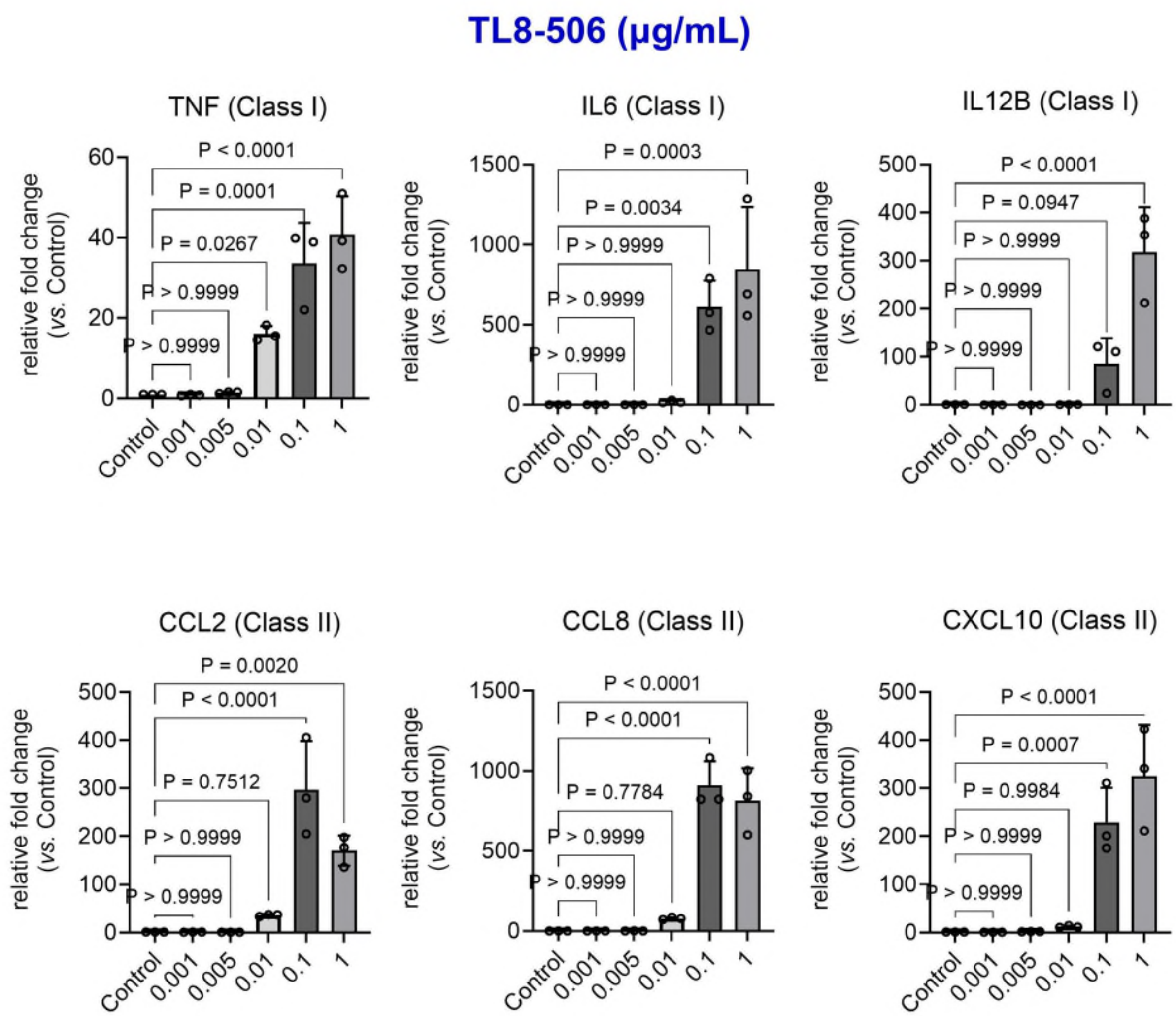

Supplementary Fig. 10. Dose-dependent induction of selected Class I and Class II genes by the synthetic TLR8 agonist TL8-506

RT-qPCR analysis of selected Class I and Class II gene expression in PBMC-derived monocytes stimulated for 4 h with increasing concentrations of the synthetic TLR8 agonist TL8-506 (0.001, 0.005, 0.01, 0.1, and 1 μg/mL). Gene expression levels are presented as fold induction relative to the housekeeping gene HPRT. Each dot represents one individual donor, with each donor representing an independent batch (n = 3). Data are shown as mean ± SD. Statistical significance was assessed using one-way ANOVA followed by Dunnett's multiple comparisons test, comparing each dose to the unstimulated control. Exact p values are shown in the figure.

- ChIP-qPCR is not the best way to examine differential activation of NFkB vs. IRF-ISGF3 axes. Additional biochemical studies would provide more direct evidence of differential pathway activation. For example: nuclear localization of IRF3, STAT1, STAT2, and IRF9 (similar to the p65 immunofluorescence in Fig. 7a), or Western blots (e.g. for phospho-IRF3, phospho-STAT1, etc), or EMSA to measure DNA-binding activity of NFkB vs. ISRE elements.

Response: We thank the reviewer for this helpful suggestion. We agree that additional biochemical evidence would strengthen the conclusions. To further assess STAT1 activation following archaeal stimulation, we performed Western blot analysis of phosphorylated STAT1.

Specifically, we examined phospho-STAT1 (Tyr701) in primary human PBMCs derived monocytes after 3 h stimulation with *M. stadtmanae* or *M. smithii*. As a positive control, we included human IFN-α stimulation for 15 min, since IFN-α is a well-established activator of STAT1 phosphorylation through the type I interferon pathway. Both *M. stadtmanae* and *M. smithii* clearly induced STAT1 phosphorylation, and we did not observe a difference between the two species **(Fig. 7f, g, Supplementary Fig. 12; newly added data).** As expected, the IFN-α control also gave a strong phospho-STAT1 signal.

We also noted a modest **increase in total STAT1 protein** relative to β-actin after archaeal stimulation. This is in line with the known positive-feedback regulation of STAT1 and is consistent with our RNA-seq data, which showed increased STAT1 transcription in response to both methanogens **(Supplementary Fig. 11a)**.

Taken together, these additional biochemical data support our ChIP-qPCR results and further strengthen the conclusion that both archaea activate a STAT1-associated response, without a detectable difference between *M. stadtmanae* and *M. smithii* at this level. These data have been

added to the revised manuscript **(Results, Lines 853-860)** and are discussed in the revised **Discussion (Lines 966-972)**.

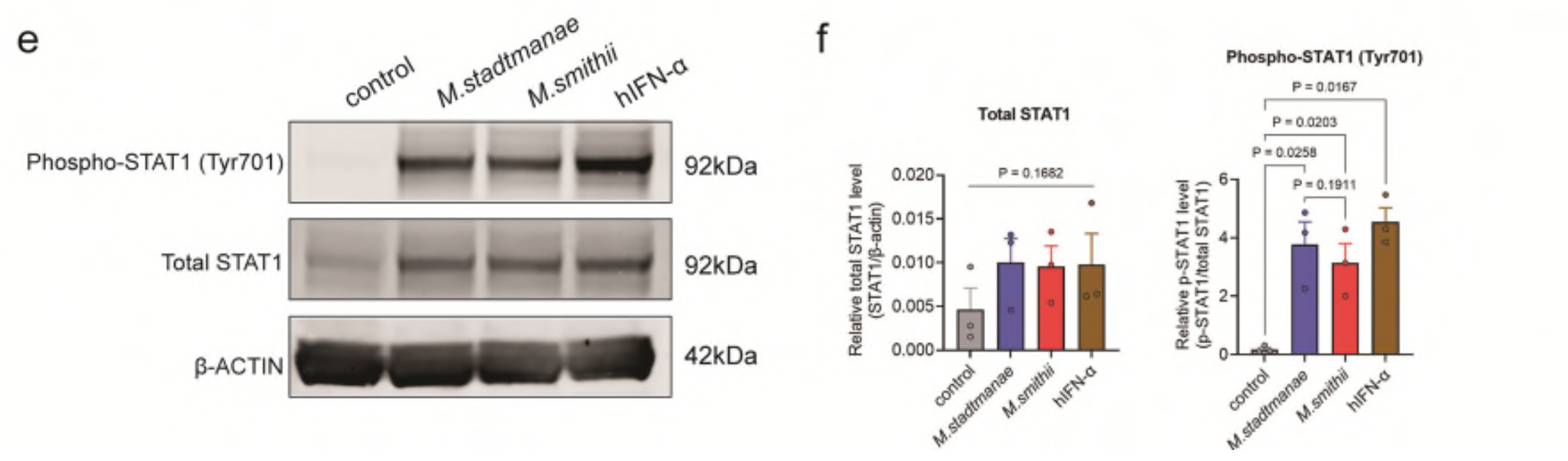

Fig.7. e Representative cropped Western blots showing phospho-STAT1 (Tyr701), total STAT1, and β-actin in primary PBMCs derived monocytes stimulated with *M. stadtmanae* or *M. smithii* for 3 h, or with human IFN-α for 15 min as a positive control. Molecular weights are indicated as follows: phospho-STAT1 and total STAT1, 92 kDa; β-actin, 42 kDa. Uncropped blots are provided in Supplementary Fig. 13. **f** Quantification of Western blot signals shown in Left, total STAT1 normalized to β-actin. Right, phospho-STAT1 normalized to total STAT1. Each dot represents one donor, corresponding to one independent batch (n = 3). Data are shown as mean ± SEM. Statistical significance was assessed by one-way ANOVA followed by Tukey's multiple comparisons test. Exact p values are indicated in the graph.

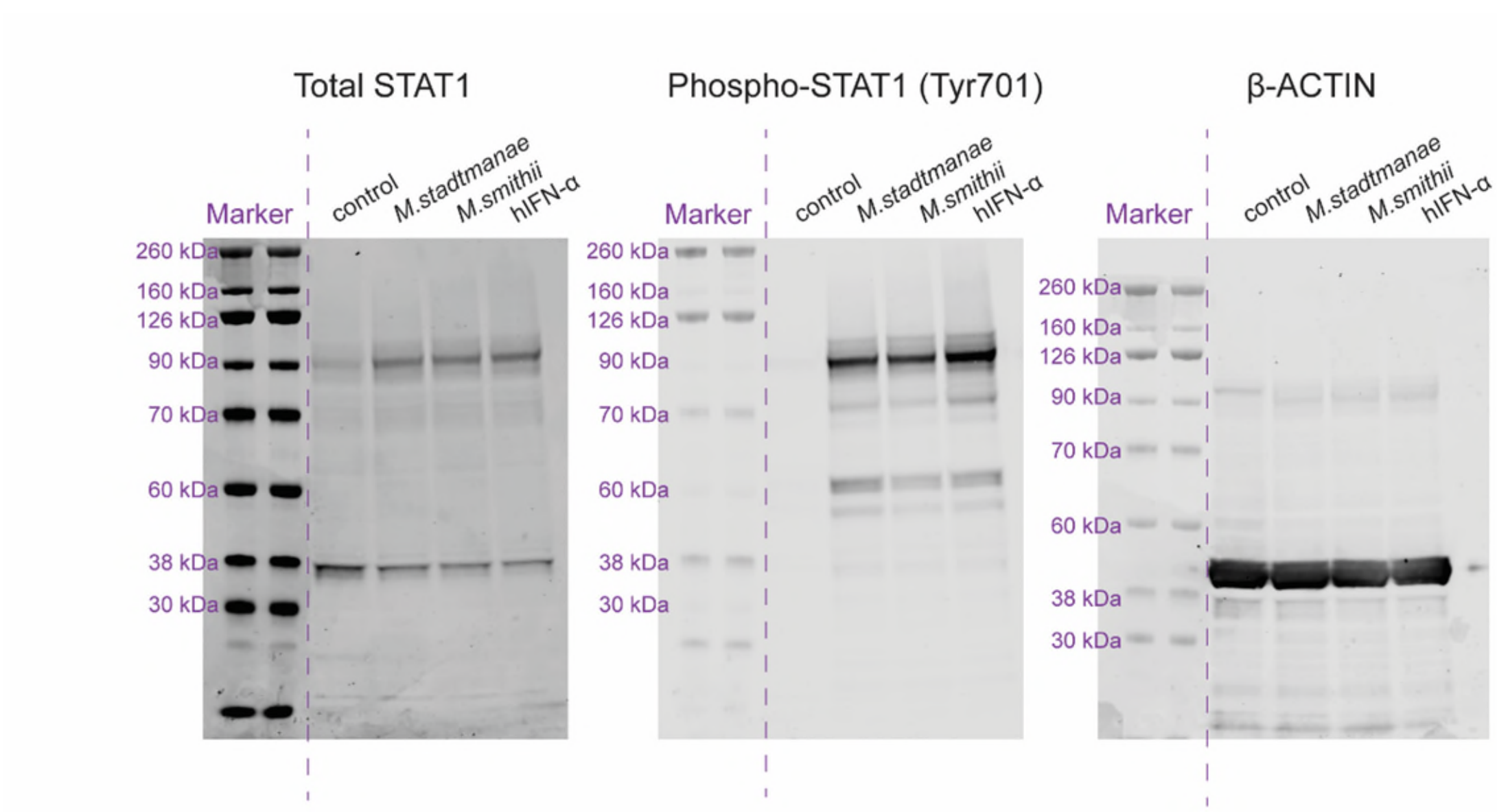

Supplementary Fig. 12. Uncropped western blot images. Representative uncropped western blot images corresponding to the blots shown in Fig. 7f, including total STAT1, phospho-STAT1

(Tyr701), and β-actin. Uncropped images of the remaining western blots are provided in the Source Data file.

– Reporting expressions of type I IFN genes would be more informative than STAT1 and STAT2 expression (current Fig 7e)

Response: We agree that reporting type I interferon genes is more informative than showing STAT1 and STAT2 expression alone. We therefore re-examined our transcriptomic dataset and analyzed normalized expression of the type I interferon gene *IFNB1*, which was the most prominently induced type I interferon transcript in our dataset, separately for each archaeon at 4 h and 24 h. For both *M. stadtmanae* and *M. smithii*, ***IFNB1* was significantly induced at 4 h**, whereas expression declined again after prolonged stimulation for 24 h. Thus, for both archaea, *IFNB1* induction is most evident at the early time point and diminishes over time. We have now included *IFNB1* as an upstream readout of the type I interferon branch in the revised manuscript. These data are shown in **Supplementary Fig. 11b (newly added data)**, and the corresponding description has been added to the Results section **(Lines 860 - 865).**

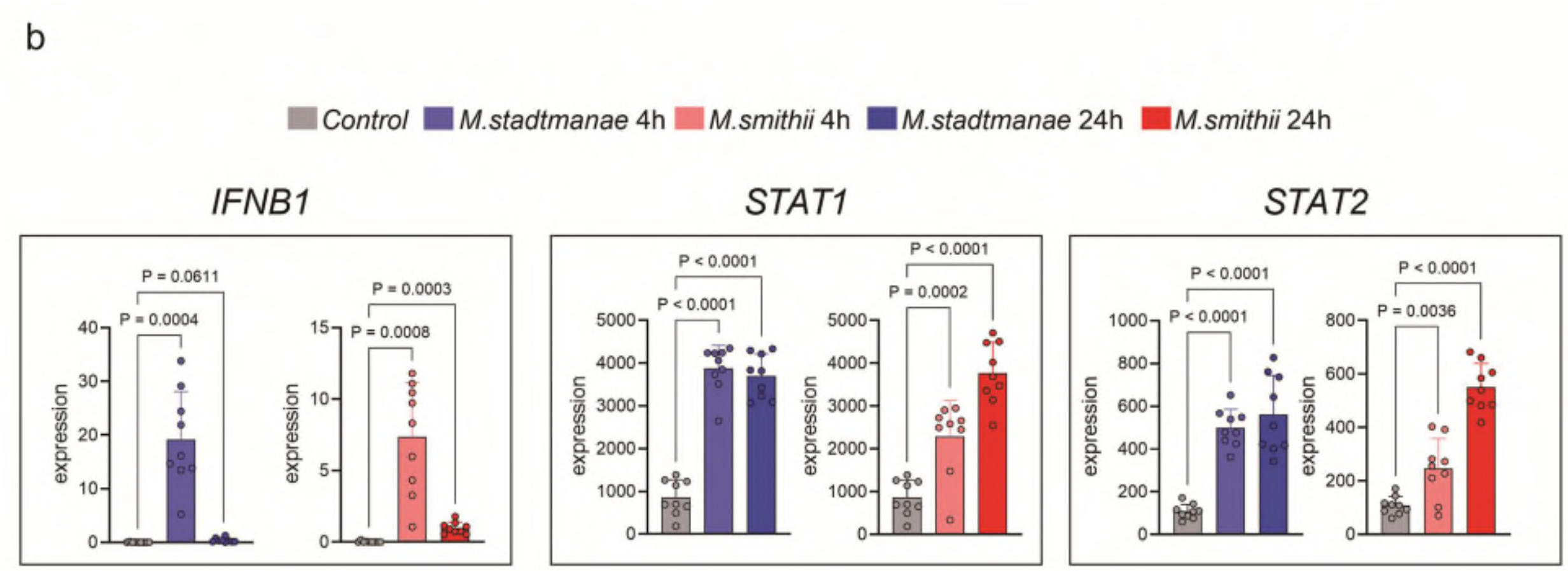

Supplementary Fig. 11. b Bar charts showing RNA-seq-derived normalized expression of the type I interferon gene IFNB1 together with STAT1 and STAT2 in PBMCs from nine healthy donors following stimulation with *M. stadtmanae* or *M. smithii* for 4 h or 24 h, compared with unstimulated controls. Each dot represents one donor (n = 9), and bars indicate mean ± SD. Statistical significance was determined by one-way ANOVA followed by Dunnett's multiple comparisons test versus the unstimulated control. Exact p values are indicated above the bars.

- Are there other studies on dose-dependent TLR responses that suggest expression of ISGF3 target genes saturate at lower doses?

Response: We therefore revisited the literature to see whether similar dose-dependent behavior has been described for other TLR pathways. While direct examples of early saturation of ISGF3 target genes in TLR systems are still limited, several studies are consistent with the idea that interferon-associated outputs can be engaged at relatively low effective stimulus levels and are regulated differently from more classical NF-κB-dominated inflammatory programs.

A recent dose-dependent TLR4 study showed that low-dose LPS preferentially induces interferon-responsive gene signatures, supporting the concept that distinct downstream transcriptional modules can operate with different activation thresholds (Commun Biol., 2022; PMID: 35091696). In addition, work on LPS signaling in macrophages showed that induction of ISGF3 activity and interferon-stimulated genes depends critically on intermediate IFN-β production and type I IFN receptor signaling, whereas canonical NF-κB activation and inflammatory cytokine programs are regulated through a different branch (J Leukoc Biol., 2014; PMID: 25024400). This supports the idea that interferon-associated outputs are not simply a weaker version of the inflammatory program but follow a distinct regulatory logic. More broadly, studies on TLR7/8 signaling have shown that IFN-associated outputs can be separated from NF-κB-dominated inflammatory outputs, again supporting the idea that these response modules are differentially regulated (ImmunoHorizons, 2020; PMID: 32086319).

ADDITIONAL MINOR CONCERNS

- PBMCs are not likely to ever interact with these archaea in vivo, and the data indicate that CD14+ cells are more important than lymphocytes. Why did the authors use PBMCs rather than monocytes or moDCs for the whole project?

Response: We appreciate this point and agree that circulating PBMCs are unlikely to encounter intact gut archaea under steady-state conditions. At the same time, PBMCs were not the only cellular system used in this study, and our experimental strategy was stepwise. We initially used PBMCs in the early phase of the project because they provided an accessible and robust primary human system in which responses could be assessed across multiple donors in a mixed leukocyte context. This was useful not only for establishing the reproducibility and overall structure of the archaeal response, but also for determining which immune cell compartment contributed most

strongly to that response. From these experiments, it became clear that $CD14^+$ monocytes were the dominant responsive population.

Based on this result, the later stages of the study were focused on purified $CD14^+$ monocytes and monocyte-derived dendritic cell-like cells. Accordingly, many of the key follow-up and validation experiments, including uptake analyses, TLR8 inhibition experiments, and downstream pathway measurements, were performed in these monocyte-centered systems rather than in PBMCs alone. **Thus, PBMCs served as the initial discovery and stratification platform, whereas the mechanistic dissection was carried out in the more directly relevant myeloid cell systems identified from that first step.** We have now inclined this point in the Discussion **(Lines 897 - 904)**

- The transcription factor motif analysis (Figure 7C) is an important piece of data that sheds light on the difference between Class I and II genes. This should be presented earlier, preferably as part of Figure 2, before any of the mechanistic experiments.

Response: We appreciate this suggestion and agree that the transcription factor motif analysis is an important part of the study. However, we prefer to retain it in the later mechanistic section because it reflects a downstream explanatory step rather than an initial observation.

The logic of the study moves from phenotype to mechanism. We first defined the Class I and Class II patterns empirically from the transcriptomic data. At that stage, it was not yet clear whether the difference between these gene groups primarily reflected transcription factor usage or instead arose from differences at the level of upstream stimulation. We therefore next examined upstream determinants and found that the two archaea differ in uptake and uptake kinetics. This provided an initial explanation for the stronger Class I bias, but it did not fully account for the behavior of the Class II genes.

This led us to further investigate response dynamics and RNA-based stimulation, which showed that Class II activation occurs at a lower effective threshold. Only after establishing these upstream and threshold-related differences did it become meaningful to ask how the downstream regulatory architecture differs between the two gene classes. In this context, the transcription factor motif analysis served as a hypothesis-generating step to explain the distinct downstream programs. For this reason, we feel that moving this analysis to an earlier figure would disrupt the logic of the

manuscript and would imply a transcriptional regulatory model before the upstream uptake- and threshold-based framework has been established.

- Are there IRF motifs that appear in the analysis of Figure 7C? If so, they should be highlighted in the figure. If not, their absence should be considered in the Discussion because ISGF3 target genes should be enriched IRF motifs.

Response: We thank the reviewer for this helpful suggestion. The original analysis shown in Figure 7c was a transcription factor prediction / upstream regulator analysis and did not directly evaluate transcription factor binding motif enrichment. We agree that this distinction is important. To address this point, we performed an additional **HOMER known motif enrichment** analysis on TSS-centered regions associated with the Class II gene set. The analysis was carried out using HOMER findMotifs.pl with regions defined as −2000 bp to +2000 bp relative to the transcription start site. Among the significantly enriched motifs, IRF1 was the top-ranked hit, and ISRE, IRF2, IRF8, and IRF3 were also significantly enriched (all adjusted $P < 0.01$). Together, these results reinforce the view that the Class II program is linked to an interferon-responsive transcriptional architecture consistent with STAT1/STAT2-associated regulation. We have added this analysis to the Methods **(Lines 355-359)** and Results **(Lines 841-847)** sections and included the data in **Supplementary Fig. 11a (newly added data)**.

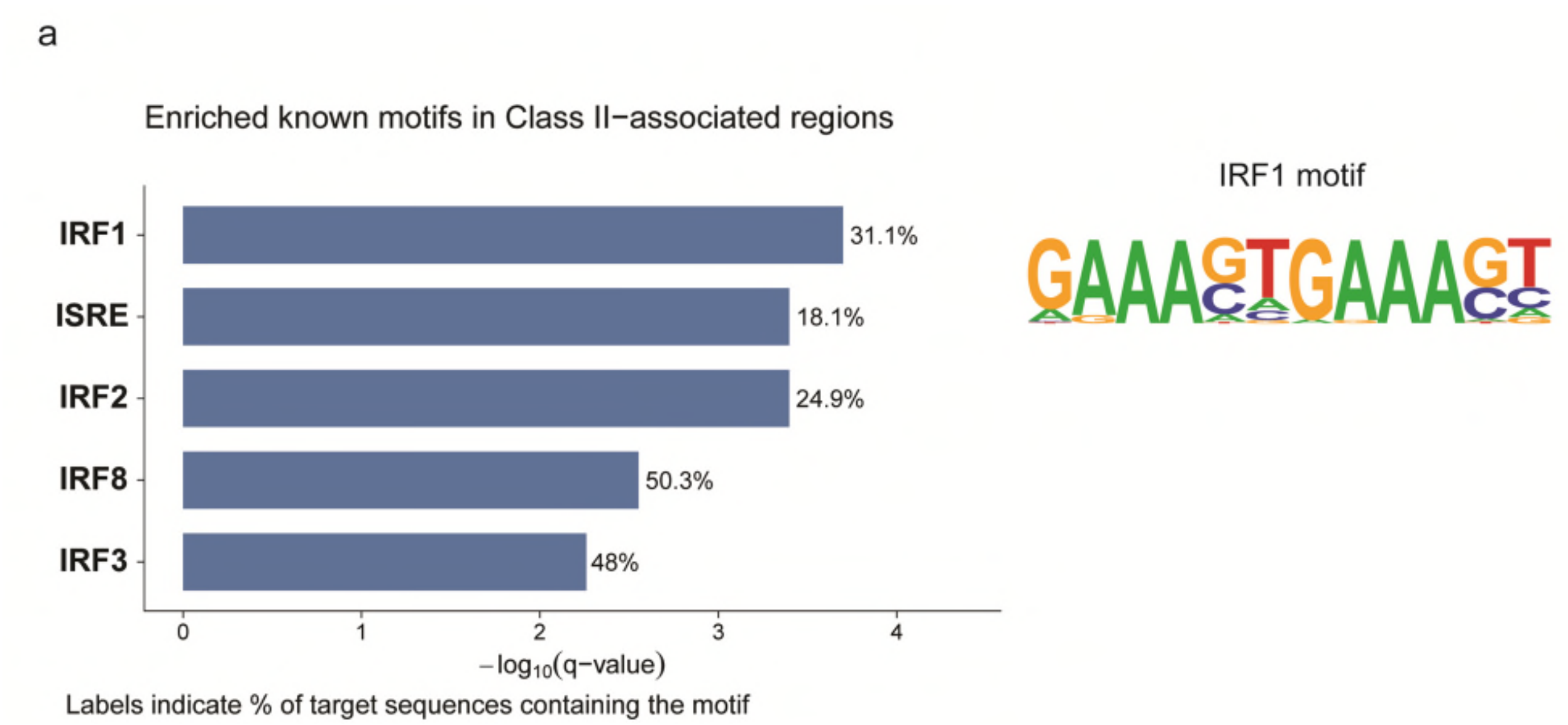

Supplementary Fig. 11. a Left, HOMER known motif enrichment analysis of genomic regions spanning −2000 bp to +2000 bp relative to the transcription start site of Class II genes. Only significantly enriched motifs with $q < 0.01$ are shown. Bar length represents motif enrichment

significance as −log10(q value), and percentages indicate the proportion of target sequences containing the motif. Statistical significance for motif enrichment was calculated by HOMER relative to sequence-matched background regions and is reported as Benjamini-adjusted q values. Right, sequence logo of the top enriched motif, IRF1.

- The writing would benefit from further editing, especially in the Introduction. The writing could be more concise and clearer.

Response: We thank the reviewer for this helpful suggestion. We revised the Introduction extensively to improve clarity and conciseness and to sharpen its focus on methanogenic archaea and their immunological functions. To achieve this, we streamlined several general background sections and removed redundant text **(see red highlighted text in the Introduction)**. At the same time, we intentionally retained a concise description of the differences between *M. stadtmanae* and *M. smithii* and added relevant references on their metabolic and genomic features. Because archaea remain far less studied than bacteria or viruses in the context of host interaction, particularly with respect to the human immune system, we believe that this background is important to frame the biological rationale for our comparative analysis.

- The interpretation of Figure 4 data (Lines 606-614) is somewhat vague

Response: We thank the reviewer for this helpful comment. We agree that the original text describing Figure 4 was not sufficiently clear, and we have substantially revised this section for clarity.

The purpose of this experiment was to resolve early response kinetics that are not captured by the 4 h time point alone. Based on our earlier uptake measurements showing significantly earlier internalization of *M. stadtmanae* than *M. smithii*, we reasoned that differences during the earliest stimulation phase could contribute to the stronger Class I induction observed with *M. stadtmanae*. **However, this uptake difference did not readily explain why Class II expression was already similar at 4 h.** We therefore hypothesized that the two species differ mainly in the kinetics of Class II induction, with *M. stadtmanae* triggering an earlier response and *M. smithii* showing delayed accumulation that narrows the difference over time. To capture these early dynamics more precisely, we extended the time course to 1, 2, 4, and 6 h.

Based on these data, we now describe Fig. 4 more precisely as showing a kinetic difference between the two gene classes **(Lines 665 - 676)**. For Class I genes, the main difference is response amplitude,

with faster and stronger induction after *M. stadtmanae* stimulation than after *M. smithii*. For Class II genes, the main difference is timing: induction occurs earlier with *M. stadtmanae*, whereas under *M. smithii* stimulation expression accumulates more slowly but reaches similar levels by 4 h and continues to increase thereafter. We also revised the wording to avoid implying that the time-course data alone establish a direct causal link between uptake and transcription.

- Figure 6b statistical comparisons should be between species, not between doses.

Response: We thank the reviewer for this comment. We agree that species-to-species comparisons are important, but in our study, these were addressed in **Fig. 6a, b (b is newly added data)**, where equal amounts of *M. stadtmanae* and *M. smithii* RNA were tested side by side. This experiment was designed to assess whether the two RNA preparations differ in intrinsic activity **at matched input.**

Fig. 6b (now Fig.6c) addresses a different question. Here, we used DOTAP-complexed RNA in a dose-titration setting to determine the minimal RNA concentration required to induce each response class. For this reason, the relevant statistical comparisons in this panel are between each RNA dose and the DOTAP-only control, rather than between the two species. **This analysis allowed us to define the effective activation threshold for each gene class.** Using this approach, Class II genes were significantly induced already at 0.1 µg/mL, whereas robust induction of Class I genes required higher RNA doses (≥ 0.5 µg/mL; Fig. 6c).

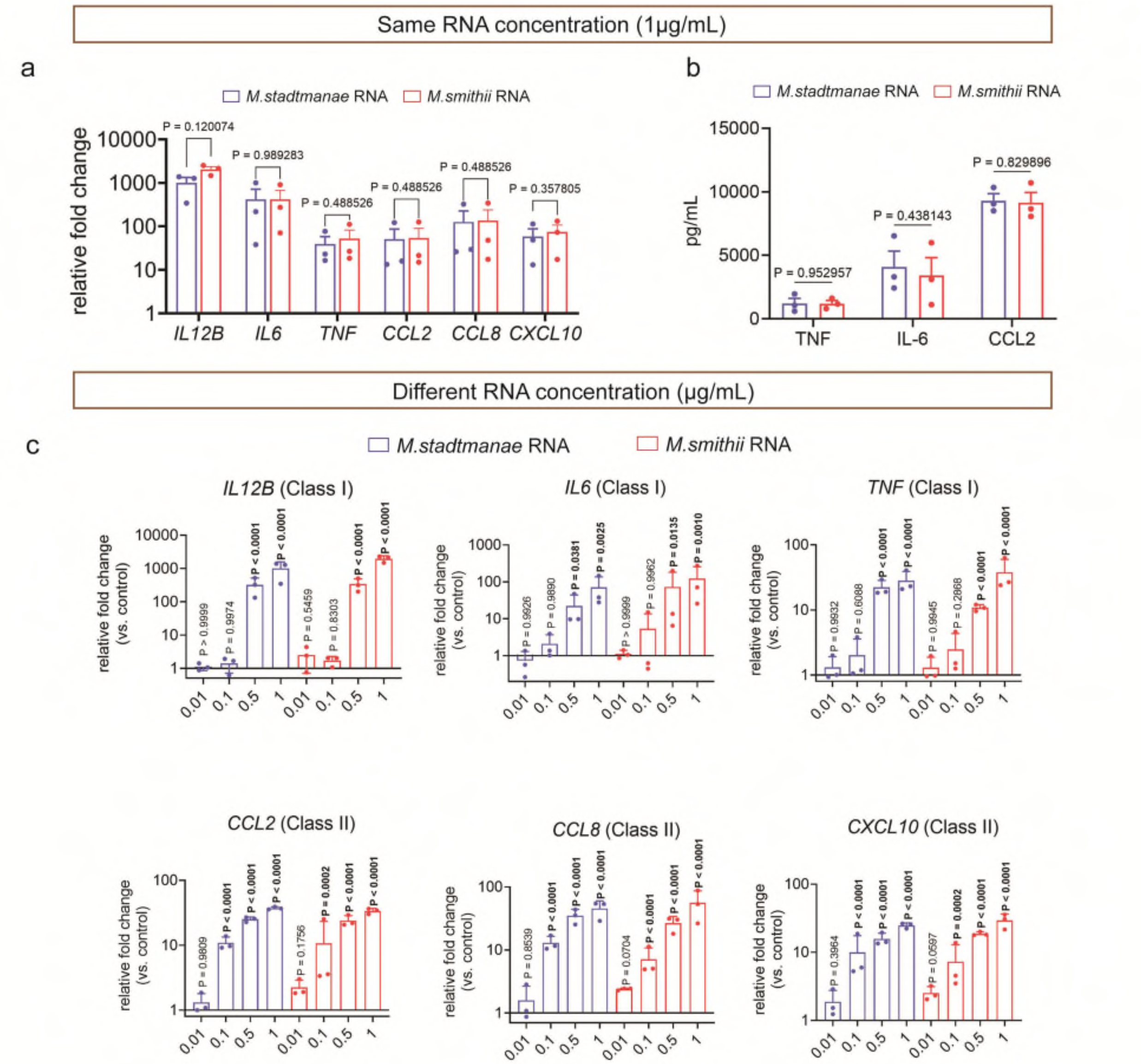

Figure 6. Selective Low-Dose Activation of Class II Genes by Archaeal RNA

a PBMC-derived monocytes were stimulated for 4 h with total RNA (1 µg/mL) extracted from *M. stadtmanae* or *M. smithii*, followed by RT-qPCR quantification of selected Class I and Class II genes. Gene expression levels are presented as fold induction relative to the housekeeping gene *HPRT*. Each dot represents one donor, corresponding to one independent batch (n = 3). Bars indicate mean ± SEM. Statistical significance was assessed using multiple paired t-tests. **b** ELISA analysis of TNF, IL-6, and CCL2 in supernatants from PBMC-derived monocytes stimulated for 4 h with total RNA (1 µg/mL) extracted from *M. stadtmanae* or *M. smithii*. Each dot represents one donor, corresponding to one independent batch (n = 3). Bars indicate mean ± SEM. Statistical

significance was assessed using multiple paired t-tests. **c** RT-qPCR analysis of selected Class I and Class II gene expression in PBMC-derived monocytes stimulated for 4 h with varying concentrations (0.01, 0.1, 0.5, and 1 μg/mL) of total RNA extracted from *M. stadtmanae* or *M. smithii* and delivered with DOTAP. Gene expression levels are presented as fold induction relative to the housekeeping gene HPRT, with the y-axis shown on a log10 scale to reflect the magnitude of induction. Each dot represents one donor, corresponding to one independent batch (n = 3). Bars indicate mean ± SD. Statistical significance was assessed using one-way ANOVA followed by Dunnett's multiple comparisons test versus the DOTAP control. Exact P values are indicated above the bars.

- What was the source of the human PBMCs? Healthy volunteers, patients at a clinic, etc?

Response: We appreciate the reviewer's question. The human PBMCs were obtained **from healthy adult volunteers**. We have now clarified this in the Methods **(Lines 117-120)** by specifying "healthy adult volunteers" in the Cell Culture section, and we also updated the Ethics Approval Statement to indicate that written informed consent was obtained from all healthy adult donors (Protocol No. Az. 12-202A). In addition, healthy adult donors were included without selection based on sex or gender. Where available, sex information was based on donor information collected at recruitment. As the study was not designed or powered to assess sex- or gender-specific effects, no stratified analyses were performed.

- Line 143 – for the moDC culture methods, please at least include what growth factors were used (with doses) and how many days monocytes were grown in culture before they were considered to be DCs.

Response: Thank you for this helpful suggestion. We have now revised the moDC culture methods to explicitly specify the differentiation factors, concentrations, and timeline. In brief, CD14+ monocytes were differentiated in the presence of GM-CSF (50 ng/mL) and IL-4 (1000 U/mL) for 7 days, and we also added the medium refresh procedure on day 3 and 5, in which half of the spent medium was replaced with fresh cytokine-supplemented medium using 2× cytokine concentrations to maintain the intended final working concentrations. These details have been incorporated in the revised Methods section **(Lines 144-149).**

- Line 349 reports 10261 genes with "significant response (adjusted p < 0.01)." I initially interpreted that to mean genes that are induced by stimulation with either archaea, which would be

an exceptionally large number of induced genes. However, Figure 1b volcano plots report a much smaller (and more reasonable) number of induced genes. What is the explanation for this discrepancy? Maybe just a wording mistake in Line 349?

Response: Thank you for pointing this out. The discrepancy arises because these numbers are based on two different analysis approaches and are therefore not directly comparable. The number 10,261 refers to genes that were significant in the global Sleuth joint model across all conditions and time points (adjusted $p < 0.01$). Thus, it reflects genes that changed significantly somewhere in the overall experimental design, without a fold-change cutoff and without implying that they were all induced in a single stimulation condition. The relatively large number is therefore not unexpected in this type of global analysis and includes genes with small effect sizes.

By contrast, the volcano plots in Fig. 1b are based on individual pairwise comparisons, such as control versus *M. stadtmanae* at 4 h or 24 h. In these plots, genes were defined as differentially expressed using both adjusted $p < 0.01$ and $|\log2 \text{ fold change}| \geq 1$. This additional filtering was used to focus on genes with clearer effect sizes and to make the plots easier to interpret. As a result, the numbers shown in the volcano plots are substantially smaller.

To avoid this confusion, we will revise the wording in Line 349 **(now Lines 414)** to clarify that the 10,261 genes represent genes significant in the global model, rather than induced genes in a specific pairwise comparison.

- Did the authors filter their RNA-seq data for genes that are not expressed in moDCs (this step is not mentioned in the Methods)?

Response: We thank the reviewer for this important point. We interpret the question as referring to whether genes not expressed in the analyzed cell populations were filtered out prior to RNA-seq analysis. In this study, RNA-seq was performed on bulk PBMCs and purified CD14+ monocytes, whereas moDCs were used only for complementary functional assays and were not included in the transcriptomic analyses. We did not apply any additional manual filtering based on presumed cell-type-specific expression or on low baseline expression in unstimulated cells, since many inducible immune genes show minimal basal expression but become robustly induced upon stimulation. Very low-abundance features were instead handled in a data-driven manner using the standard filtering steps of the kallisto/sleuth workflow prior to differential testing.

- What type of multiple comparisons correction is being used to adjust p-values for RNA-seq data?

Response: Multiple testing correction was performed within **sleuth** (performed by default). Specifically, sleuth reports both raw *p* values (*pval*) and false discovery rate (FDR)-adjusted *q* values (*qval*), where *qval* corresponds to **Benjamini-Hochberg** adjusted *p* values. Accordingly, all "adjusted *p* values" reported in the manuscript refer to multiple testing adjusted q-values controlling the false discovery rate. We have now clarified this in the Methods **(Lines 215-216)**.

- Figure 1c – 3D PCA is difficult to interpret. Would prefer to see two separate 2D PCA plots (e.g. PC1 vs PC2, PC1 vs PC3)

Response: Thank you for this suggestion. We agree that the 3D PCA visualization can be difficult to interpret in a static figure. We therefore replaced the 3D PCA with two separate 2D PCA plots, showing PC1 versus PC2 and PC1 versus PC3, and included both panels in **Fig. 1c** in the revised manuscript **(Lines 438-442)**.

- Figure 1 legend – define "BP" and "MF" within the legend text

Response: Thank you for pointing this out. We have updated the Fig. 1e legend to define the abbreviations, specifying that BP denotes Gene Ontology Biological Process and MF denotes Gene Ontology Molecular Function. During revision, we also corrected a **minor plotting error in Fig. 1e**. This adjustment does not affect the overall interpretation or the conclusions drawn from the figure.

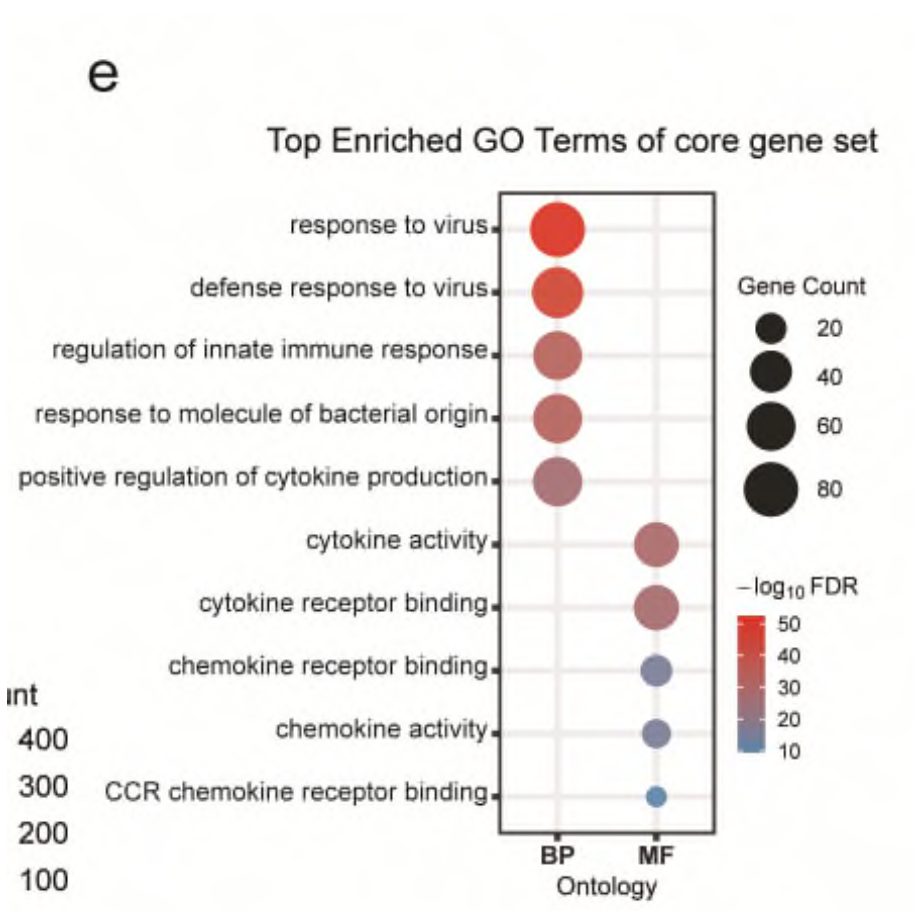

Fig.1 e A bubble plot displays the top five enriched GO terms in the categories of BP and MF, based on the 443 core genes in response to both *M. smithii* and *M. stadtmanae* at 4 h and 24 h. BP denotes gene ontology biological process and MF denotes gene ontology molecular function.

Bubble size indicates the number of core genes annotated to each term, while color represents the FDR for enrichment significance.

- Figure 3e, one of the microscopy labels is incorrect, should be 4h instead of 2h

Response: Thank you for noticing this. In the course of revision, we removed this microscopy-based analysis and replaced it with a more specific approach to quantify archaeal phagocytosis using an **archaeal-specific antibody**. The revised phagocytosis data are now presented in **Fig. 3f (newly added data)**.

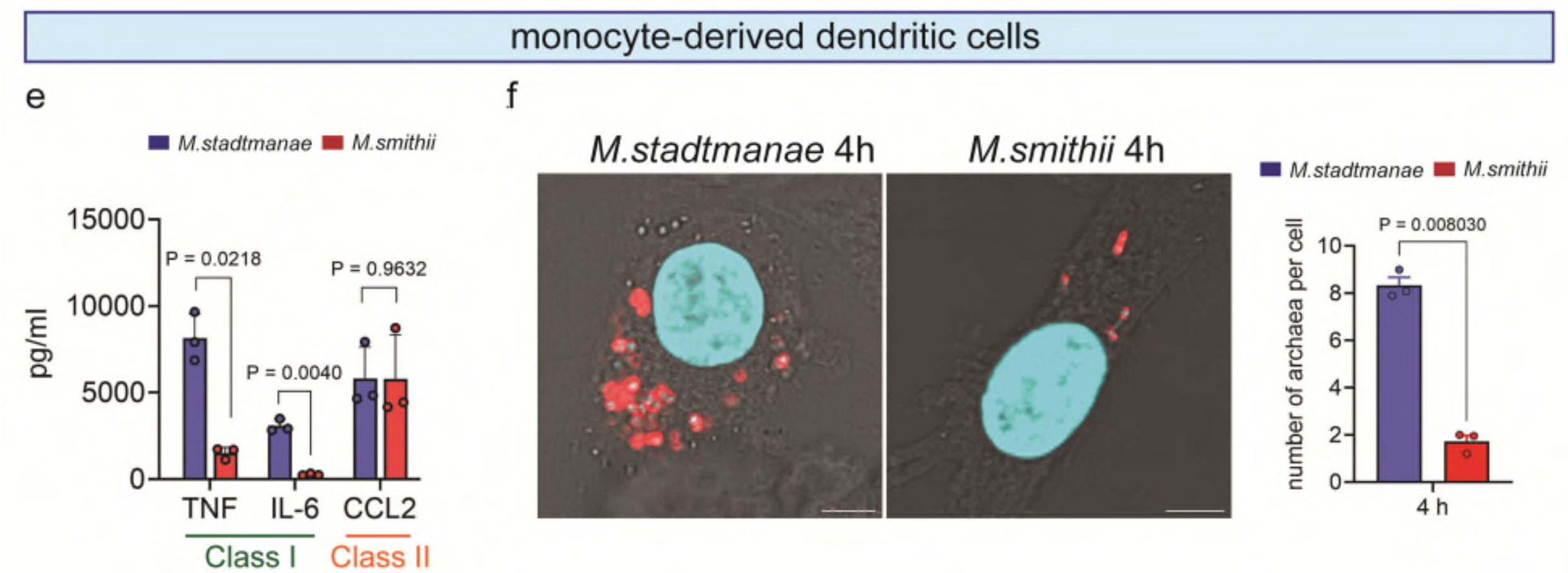

Fig. 3. f Left panel: representative confocal images and quantification of archaeal uptake in moDCs after stimulation with *M. stadtmanae* or *M. smithii* for 4 h. Archaea were visualized by species-specific immunofluorescence staining and appear as red fluorescent ring-like structures. Nuclei were counterstained with DAPI (cyan), and phase-contrast images show cell morphology and outline. Images were acquired with a 63× oil immersion objective. Displayed images represent enlarged views of selected regions to better visualize archaea-associated signals. Scale bar = 5 µm. Right panel, quantification of the number of internalized archaea per cell, with each red ring-like structure counted as one archaeal cell. For each condition, 30-61 cells from 3 independent donors/batches were analyzed. For each donor, data were first summarized across the analyzed cells. Each dot represents the mean uptake load for one donor. Data are shown as mean ± SEM. Statistical significance was assessed using a two-tailed paired test. Exact P values are shown in the figure.

Reviewer #3 (Remarks to the Author)

The manuscript submitted by Xu and colleagues entitled "Differential methanogenic archaea-induced TLR8-dependent signaling is governed by NF-κB p65- and STAT1/2-controlled gene classes" deals with divergent M. stadtmanae and M. smithii induced immune responses in human PMBCs and monocytes. They propose a model in which differential uptake leads to quantitative differences in intracellular archaeal RNA, sensed predominantly via TLR8, resulting in high-threshold NF-κB–driven 'Class I' responses versus low-threshold STAT1/STAT2–driven 'Class II' responses. In general, the authors address an exciting topic.

The topic is novel and of broad interest to immunological research, and the experimental design is generally strong and internally consistent. Overall, the manuscript is too long and the paragraphs should be much more focused. In addition, some major and minor issues should be addressed to strengthen the mechanistic conclusions and improve clarity.

Response: We thank the reviewer for this constructive overall assessment. In the revised manuscript, we have shortened and streamlined the text throughout to improve focus and clarity. Because the study includes multiple layers of results and mechanistic detail, some more extensive description is unavoidable. However, we have made every effort to reduce redundancy and present the findings as clearly and concisely as possible.

Major points:

1. The use of "Class I" and "Class II" to describe transcriptional programs is potentially misleading, as these terms are strongly associated with MHC class I/II in immunology. This may cause confusion, particularly given the immune context of the study. The authors should consider renaming these programs or explicitly clarifying that this classification is unrelated to antigen presentation.

Response: We appreciate this important point and agree that the terms "Class I" and "Class II" may be confused with MHC class I/II in an immunological context. To avoid this, we have added an **explicit clarification** at the first mention of these groups, in the relevant Results **(Lines 550-551)** section, and in the figure legend, stating that these are operational labels for expression-defined gene modules and are unrelated to MHC class I/II. In addition, we replaced the term "programs" with "modules" throughout to make this distinction clearer.

2. Pharmacologic inhibition with CU-CPT9a strongly suggests TLR8 as the dominant upstream sensor. However, reliance on a single antagonist is insufficient to fully establish specificity. Concerning the data in Figure 5b: what are the TLR8-independet effects? Additional controls demonstrating that non-TLR8 pathways are unaffected (e.g. responses to other TLR agonists) or orthogonal validation (genetic knockdown) would substantially strengthen this claim. Conclusions regarding TLR8 exclusivity should be phrased more cautiously.

Response: We agree with the reviewer that our original wording was too strong, and we have revised the manuscript to avoid implying TLR8 exclusivity based on inhibitor data alone.

First, to address potential **off-target effects** of CU-CPT9a, we tested responses to Pam3CSK4, a TLR2/1 agonist, and LPS, a TLR4 agonist, in the presence of the inhibitor under the same experimental conditions. CU-CPT9a did not reduce responses to either stimulus, indicating that these non-TLR8 pathways remained functional and supporting pathway selectivity in our system **(Supplementary Fig. 8a; newly added data)**. We also cite prior work showing that CU-CPT9a is a highly selective human TLR8 antagonist (Nat Chem Biol., 2018, PMID: 29155422; Cell Chem Biol., 2018, PMID: 30100350).

Second, to provide orthogonal genetic validation, we added experiments using the BLaER1 monocyte model together with the corresponding **TLR8 knockout line**. BLaER1 cells are a well-established model for studying innate immune signaling in human monocytes (Methods Mol Biol. 2018; PMID: 29177855). The TLR8 knockout BLaER1 line used here was established and functionally validated in our previous study on archaeal sensing (Front. Immunol. 2017; PMID: 29181003), and the relevant procedures are now described more clearly in the Methods.

To address this point more directly, we used the TLR8 KO model to examine **two gene categories** emerging from the CU-CPT9a analysis **(Fig. 5c, newly added data)**. The first category comprised genes that were **markedly reduced by CU-CPT9a but remained above the DEG cutoff** because of their strong induction. For these genes, we asked whether they were nevertheless fully TLR8-dependent. The second category comprised genes that were **unaffected by CU-CPT9a**. For these genes, we asked whether they were indeed TLR8-independent.

To validate the model, we stimulated control and TLR8 KO cells with the TLR8 agonist TL8-506 and with LPS. As expected, TLR8 KO cells failed to respond to TL8-506, whereas LPS responses were preserved, confirming selective loss of TLR8 signaling with intact non-TLR8 responsiveness

(Fig. 5d, newly added data). We then examined representative genes from the two categories defined above. *IL6*, which was strongly reduced by CU-CPT9a but remained above the DEG cutoff because of its high induction, was abolished in TLR8 KO cells. This shows that the residual expression observed after inhibitor treatment does not reflect TLR8 independence, but rather incomplete suppression of the response by CU-CPT9a. In contrast, *SLC7A11*, a representative redox-associated gene that was unaffected by CU-CPT9a, also remained unchanged in TLR8 KO cells. Together, these data indicate that genes such as *IL6* remain fully TLR8-dependent **despite residual expression** after inhibitor treatment, whereas the CU-CPT9a-insensitive gene set represents a TLR8-independent component of the archaeal response.

To further characterize the genes that remained upregulated in the presence of CU-CPT9a, we analyzed the 107 transcripts that were still induced under inhibitor treatment. Interaction network analysis showed that a subset of these genes formed three connected clusters, including a CD276/IL3RA-centered cluster, a redox-associated cluster organized around the SLC7A11-GCLM axis, and an immediate-early signaling cluster featuring DUSP1 (**Fig. 5e**, **newly added data)**. To complement this network-based analysis, we also performed Reactome pathway enrichment analysis on these 107 genes. This revealed strong enrichment of NFE2L2-related signaling, with the top pathways including “NFE2L2 regulating antioxidant/detoxification enzymes” and the “KEAP1-NFE2L2 pathway” **(Supplementary Fig. 8g)**, which is consistent with the redox-associated cluster identified in the interaction network. We have retained these data in the revised manuscript because they help define the inhibitor-insensitive component of the response more clearly.

Accordingly, we revised the Results **(Lines 686-693 and 715-748)** and Discussion **(Lines 917-933)** to describe TLR8 as the dominant upstream sensor in this setting, while avoiding claims of complete exclusivity.

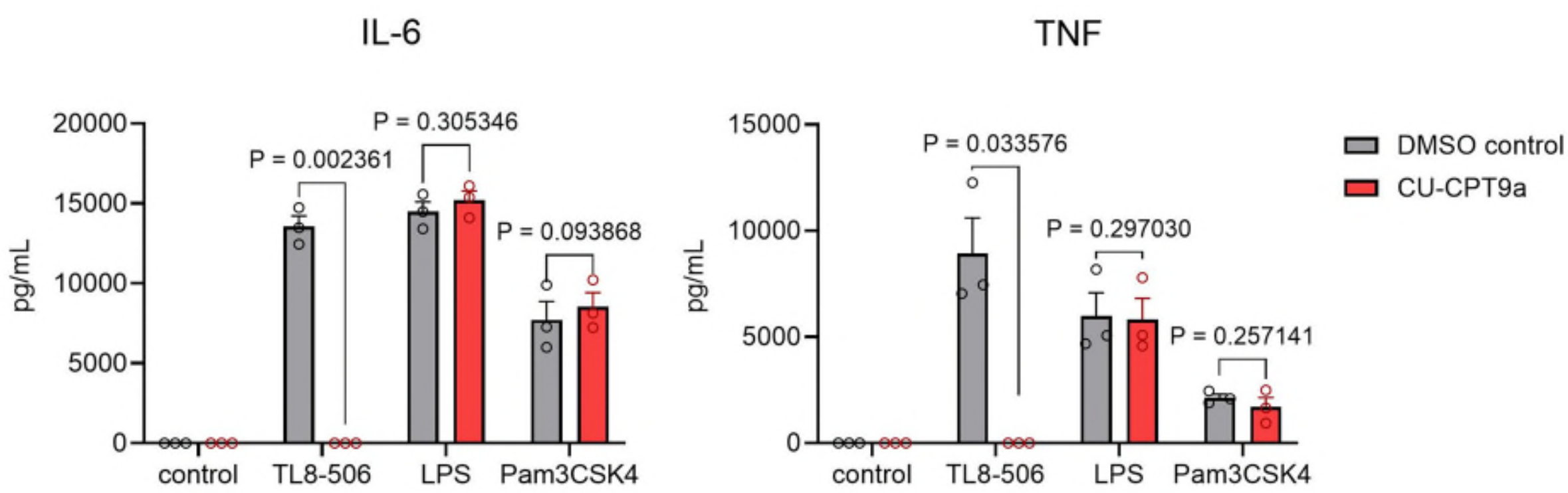

Supplementary Fig. 8a PBMC-derived monocytes were pre-treated with the TLR8 antagonist CU-CPT9a, CU-CPT9a alone, or the corresponding vehicle control (DMSO; matched final concentration in all conditions), and subsequently stimulated as indicated. Secreted IL-6 and TNF in culture supernatants were quantified by ELISA. Data are shown as individual donors (n = 3) with summary statistics (mean ± SEM). Each donor represents an independent experimental batch. Statistical significance was assessed by multiple paired t-tests. Exact adjusted P values are indicated in the figure.

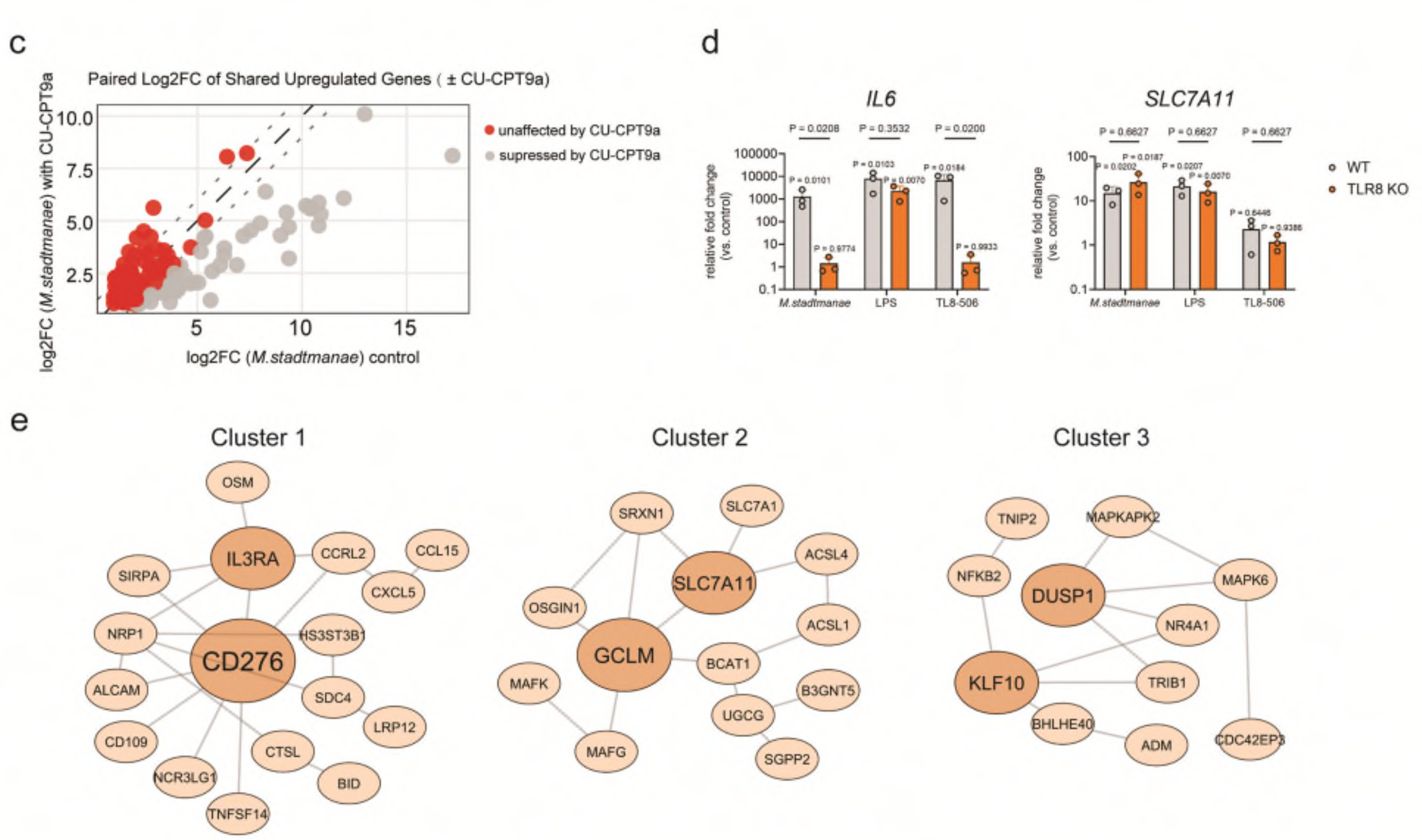

Fig. 5 c Dot plot showing paired log2FC values of 171 shared upregulated genes. Each dot represents one DEG detected under both *M. stadtmanae* stimulation alone and *M. stadtmanae*

stimulation in the presence of CU-CPT9a. The x-axis shows the log2FC under *M. stadtmanae* stimulation alone, and the y-axis shows the corresponding log2FC in the presence of CU-CPT9a. The long dashed diagonal line (y = x) indicates equal expression between conditions. Two additional short dashed lines, drawn parallel to the diagonal at ±1 log2FC difference, define two response categories based on CU-CPT9a sensitivity, where Δlog2FC is defined as log2FC in the absence minus log2FC in the presence of CU-CPT9a: (1) gray dots, 64 genes markedly reduced by CU-CPT9a (Δlog2FC ≥ 1); and (2) red dots, 107 genes largely unaffected by CU-CPT9a (Δlog2FC < 1). **d** RT-qPCR analysis of *IL6* and *SLC7A11* expression in WT and TLR8 KO BLaER1 cells following stimulation as indicated. TL8-506 and LPS were included to confirm selective loss of TLR8 signaling in TLR8 KO cells. Data are shown as mean ± SD from three independent batches. Statistical significance was assessed by one-way ANOVA with Dunnett's multiple comparisons test for comparisons of each stimulated group with its corresponding unstimulated control within the same genotype. These p values are indicated above the bars. Comparisons between WT and TLR8 KO cells under each stimulation condition were performed using multiple t-tests, and the corresponding p values are indicated above the connecting lines. **e** Interaction network analysis of the 107 genes that remained upregulated in the presence of CU-CPT9a. High-confidence interactions (STRING score ≥ 0.9) were retrieved from STRING and visualized in Cytoscape, revealing three connected clusters.

3.The DOTAP-mediated RNA delivery experiments are central to the threshold model, but the manuscript lacks sufficient controls to exclude confounding by RNA contaminants (e.g. endotoxin, DNA, protein).

Response: We agree that additional controls are important to exclude potential confounding by contaminants in DOTAP-mediated RNA delivery experiments.

First, we addressed the possibility that residual **DNA** contributes to the observed activity. During RNA isolation, samples were treated with DNase to minimize DNA carryover. In our previous study, DOTAP-complexed *M. stadtmanae* DNA failed to induce any cytokine release in human PBMCs (Front Immunol., 2017; PMID: 29181003). To directly verify this again under the conditions used in the present study, we tested DOTAP-complexed DNA isolated from both *M. stadtmanae* and *M. smithii* in PBMCs and primary monocytes. In both systems, DNA from neither species triggered detectable cytokine production, in clear contrast to archaeal RNA

(Supplementary Fig.9d; newly added data). Together, these findings strongly argue against residual DNA as the functional source of the observed activity.

Second, we directly tested whether RNA itself is required for activity. In our previous work, RNase A treatment abolished the immunostimulatory activity of *M. stadtmanae* RNA, indicating that the response depends on RNA rather than on another co-purified component (Front Immunol., 2017; PMID: 29181003). In the present study, we observed the same pattern, **with RNase A treatment fully abolishing IL-6 and IL-1β induction** after DOTAP-mediated delivery of RNA from both archaea **(Supplementary Fig. 9d; newly added data)**. Although TLR8 senses RNA degradation products, these are thought to arise through controlled endolysosomal processing, including RNase T2-dependent cleavage, after RNA uptake (Cell., 2019; PMID: 31778653). In contrast, RNase A treatment before stimulation is expected to degrade the RNA substrate prematurely and thereby prevent the generation of stimulatory ligands inside endosomes (Cell., 2019; PMID: 31778653). Taken together, these data further support that RNA is the functional stimulatory component in our preparations.

Third, we examined whether the response depends on **TLR8-mediated RNA sensing**, providing an additional functional test of RNA specificity. In newly added experiments, cytokine release induced by DOTAP-complexed archaeal RNA was completely abolished by the selective TLR8 antagonist CU-CPT9a, including IL-6, TNF, CCL2, and IL-1β **(Supplementary Fig. 9c, newly added data)**. Given that TLR8 is an endosomal sensor of ssRNA, this complete pharmacological blockade strongly supports that the measured activity reflects intracellular sensing of archaeal RNA rather than a nonspecific effect of the preparation.

These data have been added to the revised manuscript **(Results, Lines 750-768)**

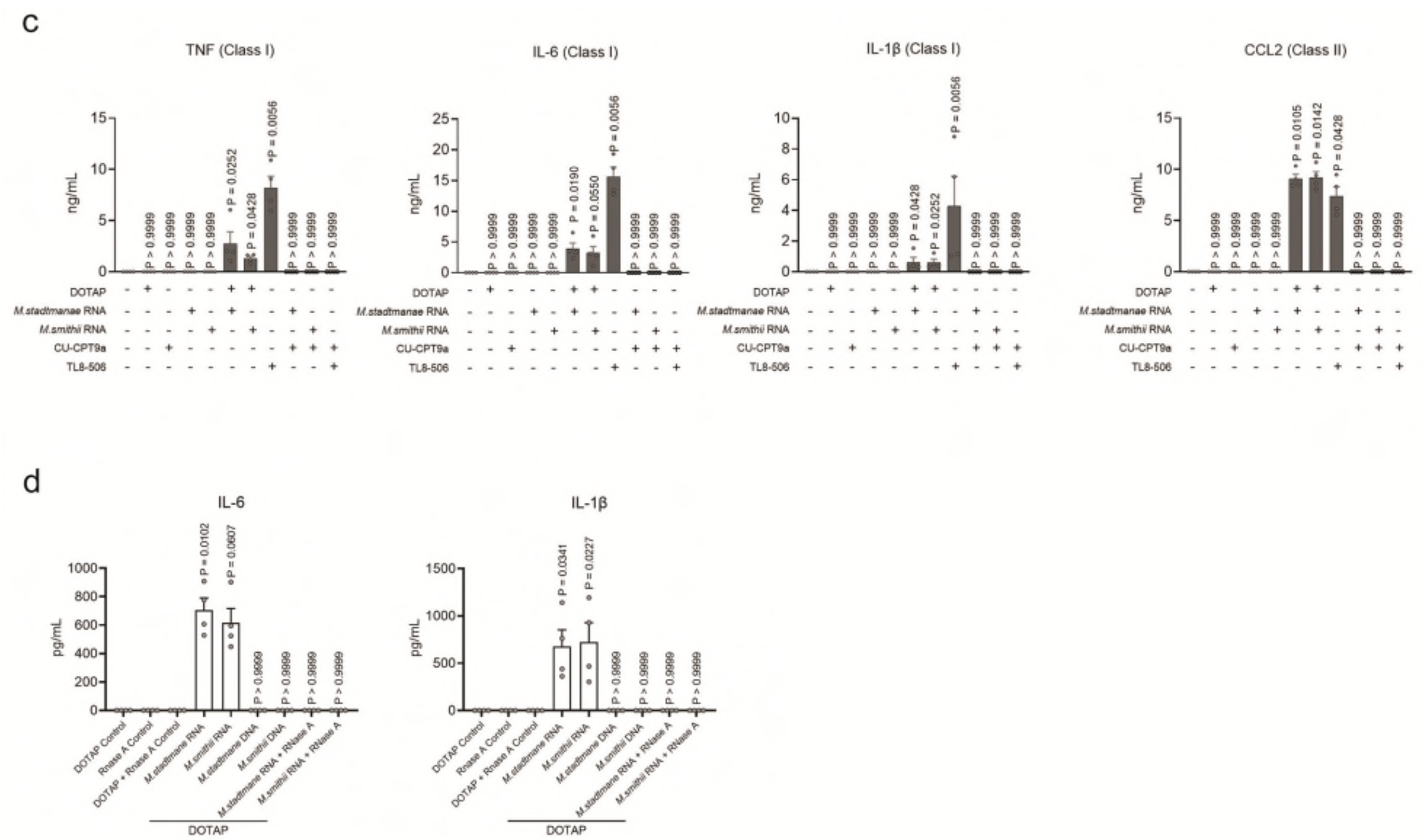

Supplementary Fig. 9 c Primary monocytes were stimulated for 24 h with archaeal total RNA (1 μg/mL), either without a delivery reagent (naked RNA) or complexed with DOTAP, in the presence or absence of the selective TLR8 antagonist CU-CPT9a. Cell-free supernatants were collected and TNF, IL-6, IL-1β, and CCL2 were quantified by ELISA. Data are shown as individual donors with mean ± SEM (n = 4 donors from 3 independent batches). Statistical significance was assessed using the Friedman test followed by Dunn's multiple comparisons test, with each condition compared against the unstimulated control. Exact P values are shown in the figure. **d** ELISA analysis of IL-6 and IL-1β after DOTAP-mediated delivery of archaeal RNA or archaeal DNA. RNase A treatment was applied only to RNA conditions, as indicated. Statistical significance was assessed by Kruskal-Wallis test followed by Dunnett's multiple comparisons test for comparisons of RNA/DNA + DOTAP versus DOTAP control, and by Friedman test followed by Dunnett's multiple comparisons test for comparisons of RNA + DOTAP + RNase A versus RNase A + DOTAP. Data are shown as individual donors with mean ± SEM (n = 4 donors from 3 independent batches). Exact P values are shown in the figure.

4. The use of F420 autofluorescence as a label-free readout of archaeal uptake is interesting (Fig. 3a); however, F420 is not exclusive to archaea and can also be found in certain bacterial taxa (for example: https://pmc.ncbi.nlm.nih.gov/articles/PMC7267189/). This raises the possibility of misinterpretation, particularly in mixed-cell or complex biological settings. To strengthen the claim

that the detected signal reflects archaeal internalization, the authors should consider complementary approaches, such as staining with archaeal-specific antibodies or other archaeal markers, to independently validate uptake. Similarly, in Fig. 3e: DAPI was used to visualize DNA, but this signal is not necessarily archaea-derived.

Response: We thank the reviewer for this important point. We agree that F_{420} is not strictly unique to archaea and that this could, in principle, complicate interpretation in complex microbial settings. However, this is not the case in our experiments, which were performed with purified archaeal preparations and primary human monocytes or moDCs under defined in vitro conditions rather than in mixed microbial samples. In this setting, F_{420} autofluorescence is a suitable label-free readout of archaeal uptake.

To further support this conclusion, we performed independent validation using species-specific antibodies against *M. stadtmanae* and *M. smithii*, which had been validated previously (Nat. Commun. 2025; PMID: 40461460). $CD14^+$ primary monocytes were isolated from PBMCs and then stimulated with whole cells of either archaeon for 1, 2, or 4 h. After fixation and immunostaining, archaeal cells were visualized by immunofluorescence microscopy as distinct red circular structures corresponding to cell wall staining. Uptake was then quantified by counting antibody-positive particles. We assessed two complementary parameters: **uptake frequency**, defined as the proportion of cells containing internalized archaea, and **uptake load**, defined as the number of internalized archaea per cell.

Distinct red fluorescent ring-like archaeal structures were detected within stimulated cells, consistent with internalized archaea **(Fig. 3a; Supplementary Fig. 6, newly added data)**. No such structures were observed in unstimulated control cells. Quantification confirmed that *M. stadtmanae* was taken up **both earlier and more efficiently** than *M. smithii* **(Fig. 3b, newly added data)**. Already at 1 h, the uptake frequency, measured as the percentage of cells containing at least one internalized archaeal cell, was markedly higher for *M. stadtmanae* than for *M. smithii* (47.66% vs 7.32%). The same pattern was observed at 2 h (78.85% vs 13.64%) and 4 h (83.08% vs 31.04%). In addition, the uptake load, quantified as the number of internalized archaea per phagocytic cell, was consistently higher for *M. stadtmanae* than for *M. smithii* at all time points examined **(Fig. 3b, newly added data)**. Together, these independent imaging analyses validated CD14+ monocytes as the principal uptake population and showed that *M. stadtmanae* is internalized earlier and more efficiently than *M. smithii*.

We also performed the same antibody-based staining and quantification in moDCs for the experiments shown in **Fig. 3f and supplementary Fig. 7 (newly added data)**. This yielded the same overall result as in monocytes. Moreover, the antibody-based analysis in MoDCs was fully consistent with our previous DAPI-based imaging, supporting the conclusion that *M. stadtmanae* is internalized more efficiently than *M. smithii* in both cell types.

We retained the F420-based analysis as a rapid label-free screening approach and complemented it with antibody-based imaging as an orthogonal validation strategy. In addition, we extended the F420 analysis by quantifying archaeal F420 signal on a per-cell basis **(Supplementary Fig.4 a-e)**.

Together, these complementary approaches consistently supported the same conclusion. These data have been added to the revised manuscript **(Results, Lines 606 – 617 and 642-644)**

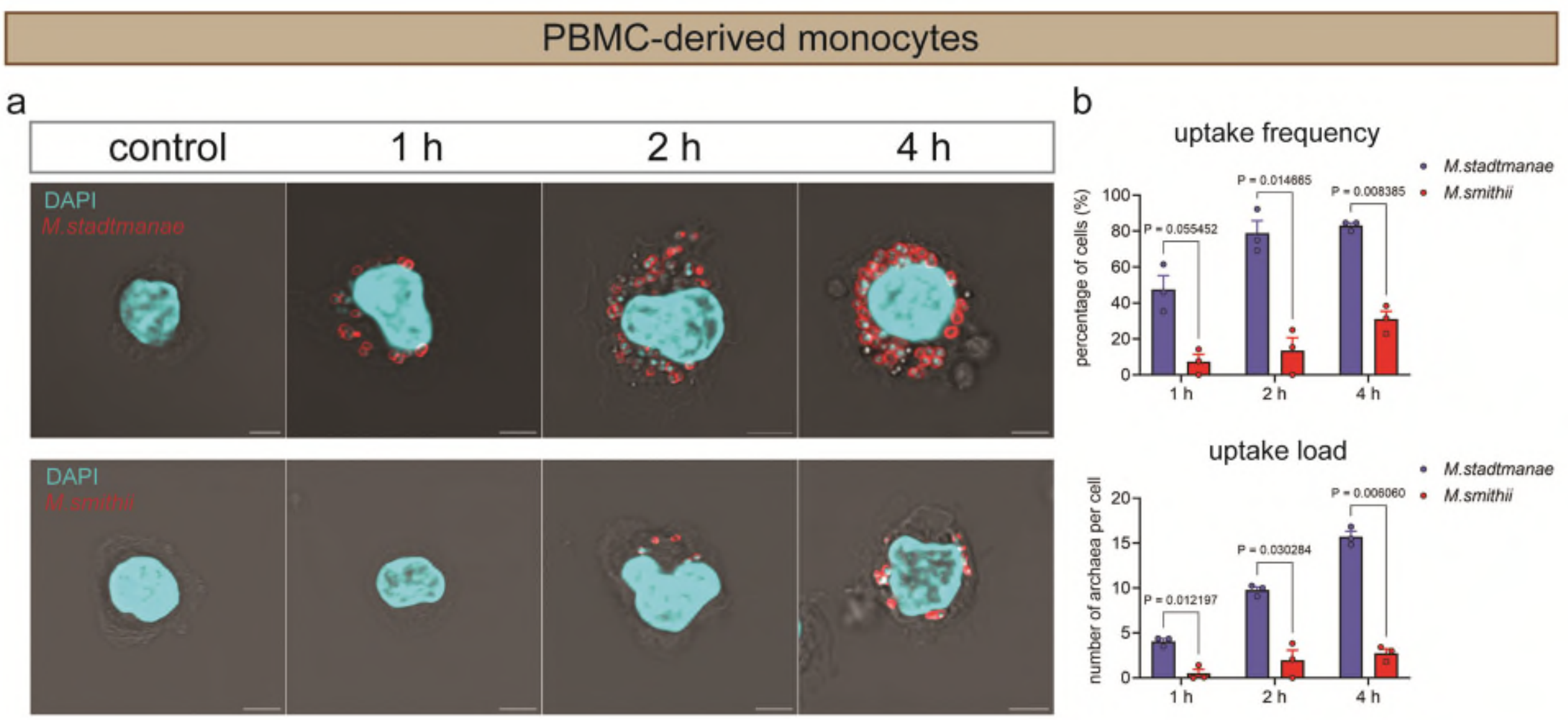

Fig. 3. a Representative merged confocal images of primary monocytes stimulated with *M. stadtmanae* or *M. smithii* for 1, 2, or 4 h, as indicated. Archaea were visualized by species-specific immunofluorescence staining and appear as red fluorescent ring-like structures. Nuclei were counterstained with DAPI (cyan). Phase-contrast images are included to show cell morphology and outline. Images were acquired with a 63× oil immersion objective. Displayed images represent enlarged views of selected regions to better visualize archaea-associated signals. Scale bar = 5 µm. **b** Quantification of archaeal uptake in primary monocytes after stimulation with *M. stadtmanae* or *M. smithii* for 1, 2, or 4 h. Upper panel, uptake frequency, defined as the percentage of cells containing at least one internalized archaeal cell. Lower panel, uptake load, defined as the mean number of internalized archaea per cell, with each ring-like structure scored as one archaeal cell.

For each condition, 30-50 cells were analyzed from 3 independent donors. For each donor, data were first summarized across the analyzed cells. In the upper panel, each dot represents the uptake frequency for one donor, and in the lower panel, each dot represents the mean uptake load for one donor. Data are shown as mean ± SEM. Statistical significance was assessed using a two-tailed paired test. P values are indicated in the figure.

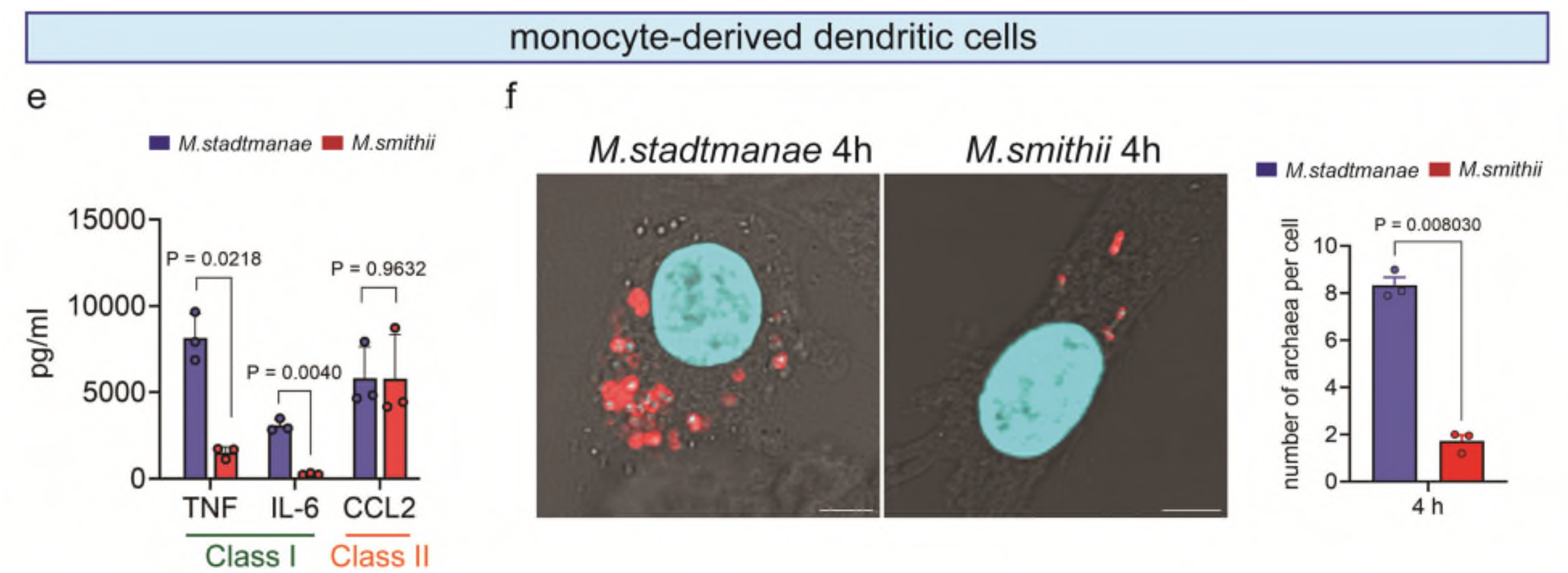

Fig. 3. f Left panel: representative confocal images and quantification of archaeal uptake in moDCs after stimulation with *M. stadtmanae* or *M. smithii* for 4 h. Archaea were visualized by species-specific immunofluorescence staining and appear as red fluorescent ring-like structures. Nuclei were counterstained with DAPI (cyan), and phase-contrast images show cell morphology and outline. Images were acquired with a 63× oil immersion objective. Displayed images represent enlarged views of selected regions to better visualize archaea-associated signals. Scale bar = 5 µm. Right panel, quantification of the number of internalized archaea per cell, with each red ring-like structure counted as one archaeal cell. For each condition, 30-61 cells from 3 independent donors/batches were analyzed. For each donor, data were first summarized across the analyzed cells. Each dot represents the mean uptake load for one donor. Data are shown as mean ± SEM. Statistical significance was assessed using a two-tailed paired test. Exact P values are shown in the figure.

5. Conceptually, the question is whether PBMCs are the primary cells that encounter archaea regularly during steady-state or infection? What about intestinal immune cells? The authors should clarify why they were investigating PBMCs and also name related limitations.

Response: We agree that intestinal immune cells, and likely also intestinal epithelial cells, are more direct host targets for methanogenic archaea in the gut, and we now state this more clearly as a

limitation of the study. At the same time, our use of PBMCs was deliberate. PBMCs provide an accessible primary human system for donor-matched and time-resolved analysis, which allowed us to define the overall response pattern first and then follow the underlying mechanisms in purified CD14$^+$ monocytes. They are also closely linked to the cell types relevant to our study, since monocytes are part of the PBMC fraction and monocyte-derived dendritic cells can be generated from them. In this sense, PBMCs served as a **practical starting point for mechanistic work** in human primary cells, rather than as a model intended to fully reflect the intestinal niche. In our study, this approach allowed us to begin with PBMCs and then narrow the key findings to monocytes and moDCs, which supports the relevance of this system for dissecting the sensing logic of methanogenic archaea. This broader relevance is also supported by **recent work from our co-authors showing that extracellular vesicles released by *M. stadtmanae* and *M. smithii* can interact not only with macrophages but also with epithelial cell lines** and induce species-specific responses (Nat. Commun. 2025, PMID: 40461460). We have revised the manuscript **(Discussion, Lines 886-896)** accordingly to clarify both the rationale for using PBMCs and the limitation that intestinal immune and epithelial cells should be addressed more directly in future studies.

Minorpoints:

1. The manuscript alternates between PBMC-based discovery and monocyte-focused mechanistic studies. While logical, this should be stated more explicitly, ideally with a schematic summarizing which experiments were performed in which cell populations and with how many donors. **Response:** We thank the reviewer for this helpful comment. We agree that the progression from the PBMC-based discovery phase to the monocyte-focused mechanistic analyses should be made easier to follow. We have therefore revised the manuscript to clarify this stepwise workflow more explicitly at the relevant transition points in the Results. In addition, we added a new summary schematic as **Supplementary Fig. 13**. This overview illustrates the experimental workflow across the different cellular systems used in the study, including PBMCs as the initial discovery platform, purified CD14$^+$ monocytes for most mechanistic follow-up analyses, and moDCs for selected validation experiments. It also indicates the main types of readouts performed in each system, including transcriptomic analysis, cytokine profiling, archaeal uptake measurements, archaeal RNA dose-response experiments, and TLR8- and transcription factor-related mechanistic analyses. **The exact donor numbers for the individual experiments are provided in the corresponding figure legends.** We hope that these changes improve the readability of the study design.

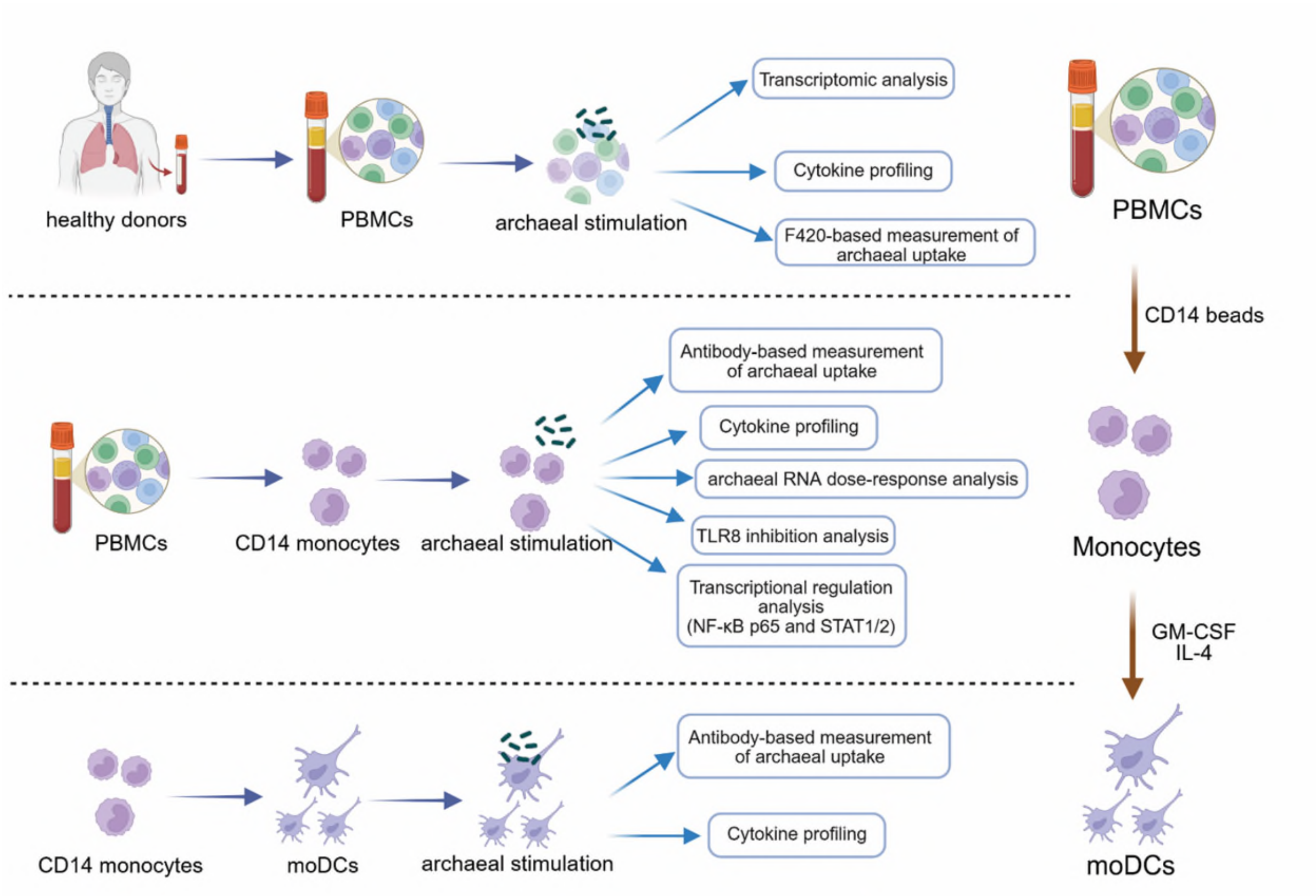

Supplementary Fig. 13 Schematic overview of the study workflow and cellular systems used in this study. PBMCs isolated from healthy donors were used as the initial discovery system for stimulation with methanogenic archaea, followed by transcriptomic analysis, cytokine profiling, and F420-based measurement of archaeal uptake. Based on these findings, purified CD14+ monocytes were used for mechanistic follow-up experiments, including antibody-based uptake measurements, cytokine profiling, archaeal RNA dose-response analysis, TLR8 inhibition analysis, and transcriptional regulation analysis focused on NF-κB p65 and STAT1/2. In selected experiments, CD14+ monocytes were further differentiated into moDCs using GM-CSF and IL-4 and analyzed for archaeal uptake and cytokine responses. [Editorial note: Created in BioRender. Xu, F. (2026) https://BioRender.com/a818mbg]

2. Donor numbers, pairing, and statistical tests should be clearly stated for each figure panel. Where possible, effect sizes in addition to p values should be provided.

Response: We thank the reviewer for this comment. We have revised the figure legends to state **donor numbers, sample pairing, and the statistical test used for each panel more clearly.** We also checked these points throughout the manuscript for consistency. In addition, **exact P values are now provided in the figures** where applicable.

This manuscript presents a compelling and conceptually coherent model for archaeal sensing in human monocytes, linking uptake efficiency, RNA dose, TLR8 signaling, and promoter-level transcriptional control. Addressing the points above, particularly terminology, RNA controls, and the strength of mechanistic claims, will significantly improve clarity and rigor and will strengthen the impact of the study.

Reviewer #4 (Remarks to the Author):

The human gastrointestinal tract has a substantial number of archaea. However, the interactions between archaea and the immune system and their roles in physiology remain poorly understood. In this paper, the authors report the systematic, time-resolved transcriptomic dissection of human PBMCs' responses to the two dominant gut methanogens. They show that class II genes have a lower activation threshold than class I genes. Previous papers, including those of the authors, have demonstrated the immunogenicity of M. smithii and M. stadtmanae in human peripheral blood mononuclear cells (PBMCs) and monocyte-derived dendritic cells, and that archaeal immune recognition is primarily driven by RNA50. Therefore, a significant drawback of this study is that the conceptual advancement derived from the results appears to be limited. Additional weaknesses are described below.

Response: We respectfully do not share the reviewer's assessment that the conceptual advance of this study is limited. Earlier work had already shown that gut methanogens can activate human immune cells and that archaeal RNA is a key driver of this response. What remained unresolved, however, was **how** this response is organized, **why** the two dominant gut methanogens produce different outputs, and whether this difference reflects distinct **downstream programs or unequal access** to a shared sensing pathway.

Because earlier studies relied largely on a limited set of readouts, they could not resolve either the breadth or the internal structure of the response. Here, time-resolved transcriptomics shows that archaeal stimulation engages a **broad innate program** with substantial overlap with host responses to bacterial and viral stimuli, moving the study well beyond a cytokine-based description. More importantly, the response is not uniform but separates into gene groups with distinct activation thresholds and regulatory features, with Class II genes induced at lower input levels than Class I genes. At a conceptual level, this suggests that innate immune output can be **organized in threshold-dependent layers rather than as a single graded response**, a principle that may extend beyond the TLR8 pathway studied here.

The new TL8-506 data further sharpen this point **(Supplementary Fig. 10, newly added data)**. Although TL8-506 activates TLR8, it does not recapitulate the preferential low-dose Class II induction seen with archaeal RNA. This difference should be interpreted with caution, since synthetic small-molecule agonists and RNA-derived ligands engage TLR8 in different ways. TL8-

506 acts through a distinct ligand-binding mode, so its failure to reproduce the archaeal RNA pattern suggests that the low-dose Class II bias may depend on features of RNA-mediated TLR8 activation, potentially including differential engagement of TLR8 ligand-binding sites. Importantly, this indicates that the preferential **low-dose induction of Class II genes is not a general feature of TLR8 activation**, but may emerge only under particular modes of RNA ligand engagement.

A further advance of the study is that it resolves the role of TLR8 at the level of gene programs rather than isolated readouts. Using primary human monocytes, transcriptomic profiling, and pharmacological TLR8 inhibition, we systematically captured the impact of TLR8 across the response. In the revised manuscript, we extended this analysis with a **TLR8 knockout system (Fig. 5c–e, newly added data)**, which allowed us to distinguish more clearly between TLR8-dependent and TLR8-independent programs. This provides a much more precise view of archaeal sensing than was possible from cytokine measurements alone. We also extended the analysis downstream of TLR8. In the revised manuscript, we added STAT1 phosphorylation data **(Fig. 7e, newly added data)** together with promoter motif analysis **(Supplementary Fig. 11a, newly added data)**, which further support the idea that the lower-threshold gene program is linked to a STAT-associated regulatory axis. This strengthens the conclusion that the Class I/Class II distinction reflects a genuine difference in regulatory control.

In addition, we strengthened the upstream arm of the study by introducing archaeal-specific antibody-based measurements of uptake dynamics, which further confirmed that ***M. stadtmanae* is taken up earlier and more efficiently (Fig. 3a, b)**. We also added multiple controls **to rule out alternative mechanisms** and to tighten the link between uptake, TLR8 engagement, and downstream gene induction.

Taken together, the study does not simply revisit archaeal immunogenicity. It defines the structure of the response, explains the apparent species difference in mechanistic terms, and strengthens this model at the levels of uptake, TLR8 dependence, downstream regulation, and experimental control. More broadly, our findings suggest that microbial RNA sensing should not be viewed simply in terms of response magnitude, but also in terms of how distinct gene programs are engaged across different activation thresholds. This provides a framework for studying **how natural ligands shape innate immune output in a more selective and layered manner.**

Major comments:

They nicely show using purified RNA that the immunostimulatory capacity of M. stadtmanae and M. smithii RNA is broadly similar. As a result, they conclude that the stronger Class I output seen with intact M. stadtmanae is best explained by enhanced cellular uptake rather than intrinsically "stronger" RNA. This raises the key question: what mechanism explains the different uptake rates of these two strains? Does M. smithii actively inhibit phagocytosis or does M. stadtmanae express a molecule that facilitates phagocytosis? Addressing this mechanism will strengthen the paper.

Response: We agree that the mechanism underlying the different uptake rates of *M. stadtmanae* and *M. smithii* is an important question. At the same time, we view this not as a single unresolved detail, but as a broader mechanistic problem that cannot yet be answered conclusively with the data currently available.

In our view, resolving this question will require work on two levels. **First, on the archaeal side,** it will be necessary to define which surface-exposed components of the two methanogens interact with host cells. This is especially relevant because archaeal cell envelope architecture differs from that of bacteria, making it unlikely that uptake-relevant ligands can simply be inferred from established bacterial systems. **Second, on the host side**, the responsible receptors and uptake pathways will need to be identified in a systematic way. Given the large number of candidate surface receptors and endocytic/phagocytic pathways in monocytes, this is likely to **require broader screening approaches** rather than a single candidate-based experiment. We therefore consider this an important next mechanistic step, and it is currently part of our ongoing follow-up work.

To address the two specific possibilities raised by the reviewer, we performed an exploratory bead-uptake assay. The idea was to test whether the strain difference could be explained by a more general effect on phagocytic capacity. If *M. smithii* broadly inhibits phagocytosis, prior exposure to *M. smithii* should also reduce the subsequent uptake of phagocytosable beads. Conversely, if *M. stadtmanae* broadly promotes phagocytosis, prior exposure to *M. stadtmanae* should increase bead uptake. Primary human $CD14^+$ monocytes were preincubated with *M. stadtmanae* or *M. smithii* for 2 h, washed to remove extracellular archaea, and then exposed to fluorescent phagocytosable beads. Under these conditions, prior exposure to *M. smithii* had little effect on subsequent bead uptake, arguing against a broad suppression of phagocytosis by *M. smithii*. In contrast, prior exposure to

M. stadtmanae reduced subsequent bead uptake, which does not support a simple model in which *M. stadtmanae* generally enhances phagocytic activity. Rather, this result is more consistent with the idea that *M. stadtmanae* itself is taken up more efficiently and that this earlier uptake affects the subsequent handling of a second particulate cargo.

At the same time, we interpret this experiment cautiously. The bead assay helps to argue against two possibilities raised by the reviewer, but it does not identify the underlying uptake mechanism or explain the strain-specific difference at the molecular level. Because these data are exploratory and do not yet provide mechanistic resolution, we have not included them in the revised manuscript. Instead, we now acknowledge this point more explicitly as an important open question and a **clear direction for future work.**

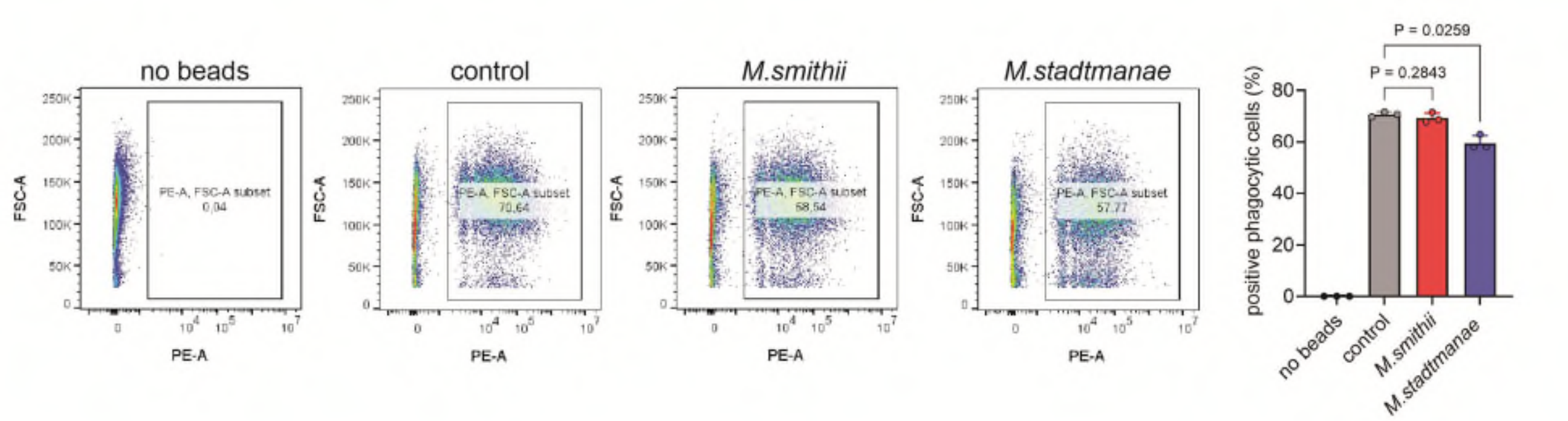

Figure Left panel: Representative flow-cytometry plots showing uptake of fluorescent beads measured in the PE channel in primary human CD14^{+} monocytes. Monocytes were either left untreated or preincubated with *M. stadtmanae* or *M. smithii* for 2 h, followed by washing to remove extracellular archaea and incubation with beads for an additional 1 h. A no-bead control is included to define background fluorescence and set the gate. Right panel: Quantification of bead uptake across three independent donors. Each dot represents one individual donor from one independent batch (n = 3). Data are shown as mean ± SD. Statistical significance was determined using one-way ANOVA. Exact P values are indicated in the panel.

Given that these two methane-forming archaeal strains reside in the gut, examining how they interact with gut epithelial cells (or even intestinal epithelial cell lines) and whether they stimulate immune responses in these cells can provide valuable basic insights.

Response: We thank the reviewer for this helpful suggestion and agree that interactions with intestinal epithelial cells are highly relevant for gut-associated methanogenic archaea. At the same time, this goes beyond the main focus of the present study.

We chose PBMCs and purified monocytes for specific experimental reasons. PBMCs provide an accessible primary human system that allows donor-matched, time-resolved, and mechanistic analyses across multiple readouts. This was particularly important for the design of the present study, because it enabled us to define the overall host response pattern first in a broader primary-cell context and then follow the key mechanism in matched $CD14^+$ monocytes, which emerged as the main archaeal uptake population in our PBMC experiments. Thus, PBMCs were used here as a **practical starting point for mechanistic work in human primary cells**, not as a model intended to fully represent the intestinal niche.

We fully agree that intestinal immune cells, and likely also intestinal epithelial cells, are more direct host targets in vivo, and we now state this more clearly as a limitation of the study. At the same time, we note that related recent work from our co-authors has shown that extracellular vesicles derived from gut methanogenic archaea, including the strains studied here, **can interact with intestinal epithelial cell lines** as well as immune cells and induce distinct host responses (Nat. Commun. 2025, PMID: 40461460). **Importantly, these responses are not identical to those observed in our PBMC-based system,** which further supports the view that different host compartments respond in different ways. Thus, PBMCs and monocytes provide a useful primary-cell reference, which will help to interpret future epithelial-cell studies rather than being replaced by them.

We therefore agree that epithelial-cell studies will be an important next step. However, we consider a dedicated epithelial-cell analysis to be beyond the scope of the present manuscript. Instead, the current study provides a necessary primary-cell framework for understanding archaeal sensing, which can now be extended in future work to more tissue-specific intestinal models.

Based on the FACS plot, M. smithii internalization rate was still lower than that of M. stadtmanae. The authors should also look at MFI to assess the phagocytosis rate per cell.

Response: We thank the reviewer for this valuable suggestion. We agree that, in addition to the frequency of F_{420} positive monocytes, a per-cell readout of archaeal uptake is informative.

We therefore performed an additional fluorescence-based analysis. First, we measured the basal F_{420} autofluorescence of free archaea under identical acquisition settings. This revealed marked strain-specific differences, with **free *M. smithii* showing substantially higher basal F_{420} signal than free *M. stadtmanae*** (mean fluorescence intensity: 4.26-fold higher; median fluorescence intensity: 2.61-fold higher; see **table 1** below, and **Supplementary Fig. 4 a, b**). These data indicate that raw monocyte-associated F_{420} intensity cannot be directly compared between strains without accounting for their different intrinsic fluorescence.

To address this, we quantified mean $CD14^+$ monocyte-associated F_{420} signal after 4 h archaeal stimulation (see **table 2** below) and calculated a normalized phagocytosis signal per cell as follows:

normalized phagocytosis signal per cell = mean $CD14^+$ monocyte-associated F_{420} intensity / mean free archaeon basal F_{420} intensity

Using this normalization, the average per-cell uptake signal was markedly higher for *M. stadtmanae* than for *M. smithii* (3.38 vs 0.71) **(Supplementary Fig. 5 c, d)**. Thus, after correction for strain-specific basal F_{420} differences, the data further support that *M. stadtmanae* is internalized more efficiently on a per-cell basis. These data have been added to the revised manuscript **(Results, Lines 595-605)**

To further support this conclusion, we performed independent validation using species-specific antibodies against *M. stadtmanae* and *M. smithii*, which had been validated previously (Nat. Commun. 2025; PMID: 40461460). Primary monocytes were stimulated with whole cells of either archaeon for 1, 2, or 4 h. After fixation and immunostaining, archaeal cells were visualized by immunofluorescence microscopy as distinct red circular structures corresponding to cell wall staining. Uptake was then quantified by counting antibody-positive particles. We assessed two complementary parameters: phagocytic frequency, defined as the proportion of cells containing internalized archaea, and uptake load, defined as the number of internalized archaea per cell.

In primary monocytes, this analysis confirmed that *M. stadtmanae* was taken up both earlier and more efficiently than *M. smithii* **(Fig.3a, b and supplementary Fig.5; newly added data)**. Already at 1 h, the phagocytic frequency was markedly higher for *M. stadtmanae* than for *M. smithii* (44.2% vs 7.5%). The same pattern was observed at 2 h (79.4% vs 18.0%) and 4 h (83.3% vs 31.1%). In addition, the uptake load was consistently higher for *M. stadtmanae* than for *M. smithii* at every time point examined. Thus, both the fraction of phagocytic cells and the number of internalized

archaea per cell supported the same conclusion. These data have been added to the revised manuscript **(Results, Lines 606-617)**

We also clarify why the 24 h uptake condition shown in the previous version was not included in the revised analysis. Because 24 h is a relatively long incubation period for assessing archaeal internalization, the F_{420}-associated signal at this time point is unlikely to reflect initial uptake alone. It may additionally be influenced by post-internalization processing, archaeal degradation, and prolonged effects on monocyte state. We therefore focused the revised analysis on the 4 h time point, which provides a more direct and interpretable measure of uptake efficiency.

Table 1. Free archaeon basal F_{420} signal

	M.smithii cells	*M.stadtmanae* cells
mean fluorescence intensity	2648	621
median fluorescence intensity	1100	421

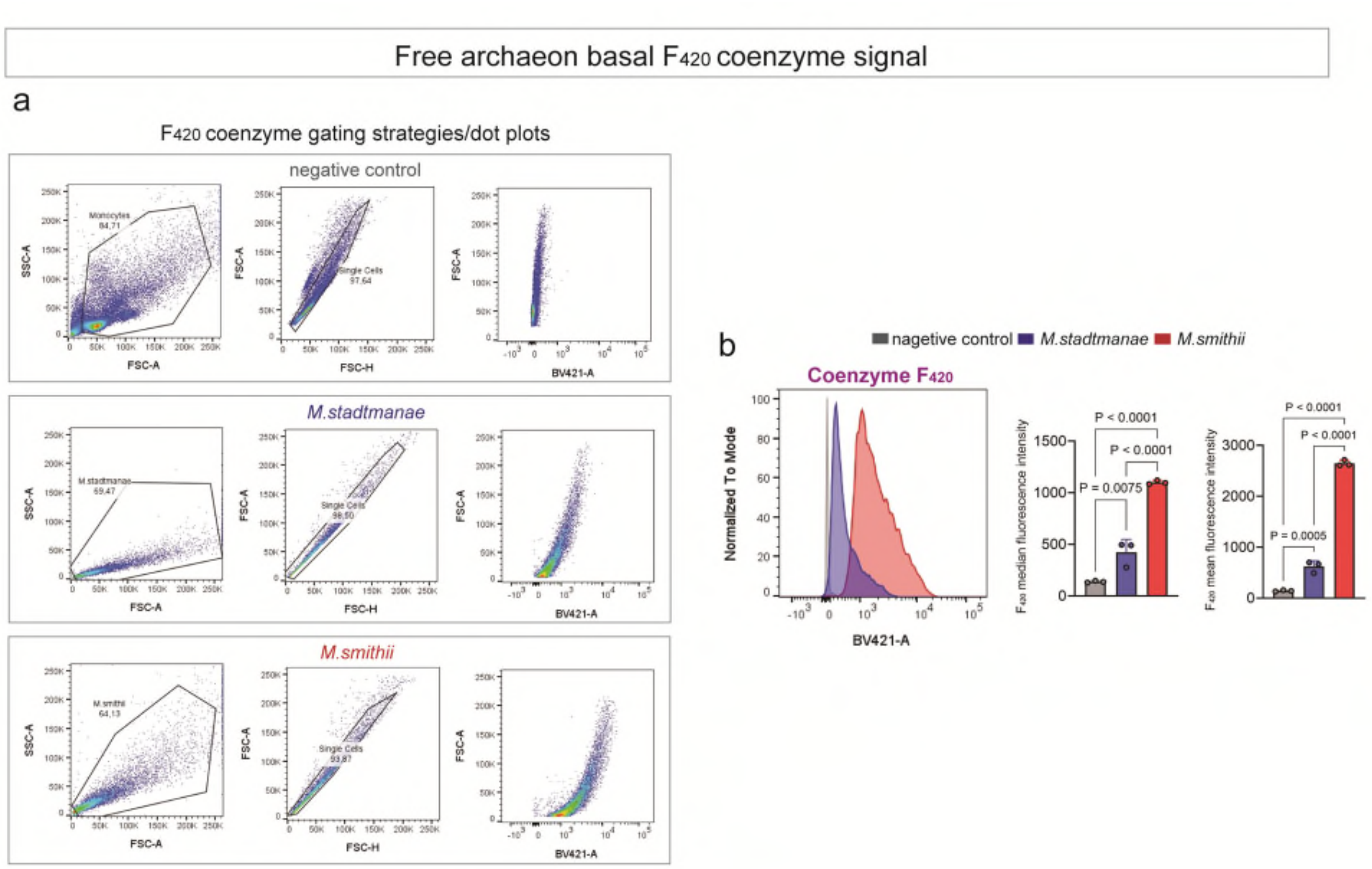

Supplementary Fig. 4. a Gating strategy for detection of archaeal F_{420} signal by flow cytometry. Cells were first gated on the main cell population based on forward and side scatter, followed by singlets, and then analyzed in the BV421 channel to detect F_{420} fluorescence. Three conditions are shown: negative control (PBMCs without archaea), *M. stadtmanae*, and *M. smithii*. **b** Left panel: Representative overlay histogram showing F_{420} fluorescence in gated cells under the indicated

conditions. Equal numbers (20000 cells) of *M. stadtmanae* and *M. smithii* cells were used for F_{420} measurement. Right panel: Quantification of F_{420} mean fluorescence intensity and median fluorescence intensity across three independent batches. Each point represents one batch. Data are shown as mean ± SD. Statistical significance was determined by one-way ANOVA followed by Tukey's multiple comparisons test. Exact p values are shown in the figure.

Table 2. $CD14^+$ monocyte-associated F_{420} signal

	F_{420}^+ monocytes stimulated with *M. smithii*	F_{420}^+ monocytes stimulated with *M. stadtmanae*
mean fluorescence intensity_batch_1	2533.47	2287.42
mean fluorescence intensity_batch_2	1051.4	1791.29
mean fluorescence intensity_batch_3	2133.48	3069.44

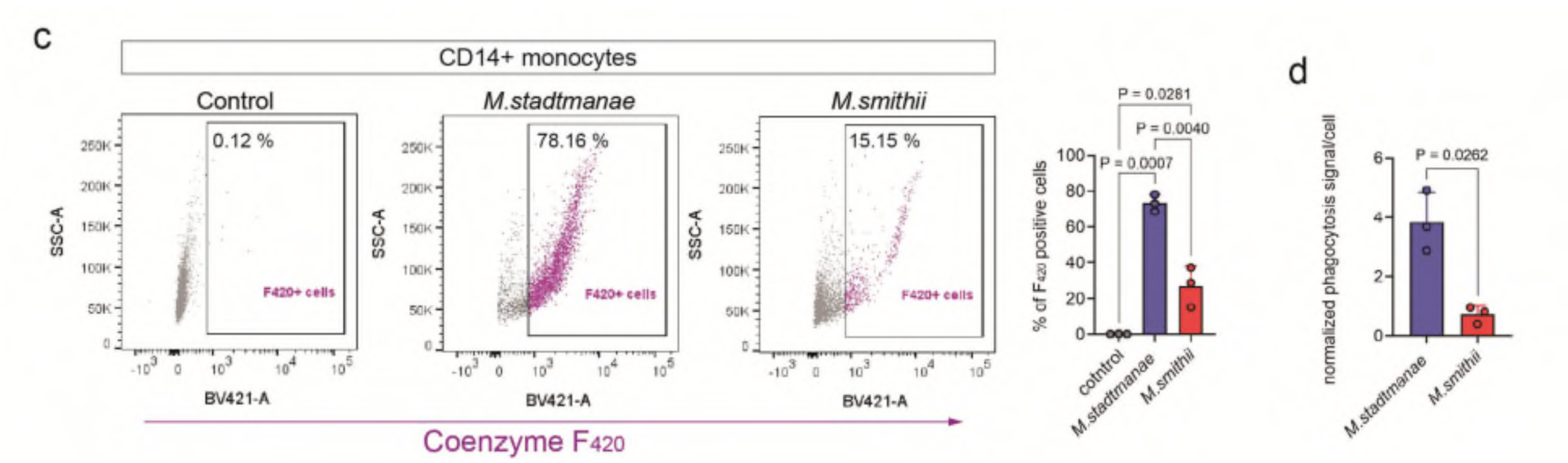

Supplementary Fig 5 c The left panel shows representative flow cytometry analysis of coenzyme F_{420} fluorescence detected in the BV421 channel in PBMCs following *M. stadtmanae* or *M. smithii* stimulation for 4 h. Cells were gated on the $CD14^+$ population from monocytes population, and F_{420}^+ cells were identified within this gate. The percentages of F_{420}^+ cells within each gated population are indicated on the plots. The right panel summarizes the percentage of F_{420}^+ cells. Each dot represents one individual donor from one independent batch (n = 3). The plots display the mean ± SD. Statistical significance was determined using one-way ANOVA followed by Tukey's multiple comparisons test. Exact P values are indicated in the right panel. **d** Quantification of the normalized phagocytosis signal per cell. For each donor, the normalized signal was calculated as the mean F_{420} fluorescence intensity associated with $CD14^+$ monocytes divided by the corresponding mean basal F_{420} fluorescence intensity of free archaea. Each dot represents one individual donor from one independent batch (n = 3). Data are shown as mean ± SD. Statistical significance was determined using a paired t-test. Exact P values are indicated in the panel.

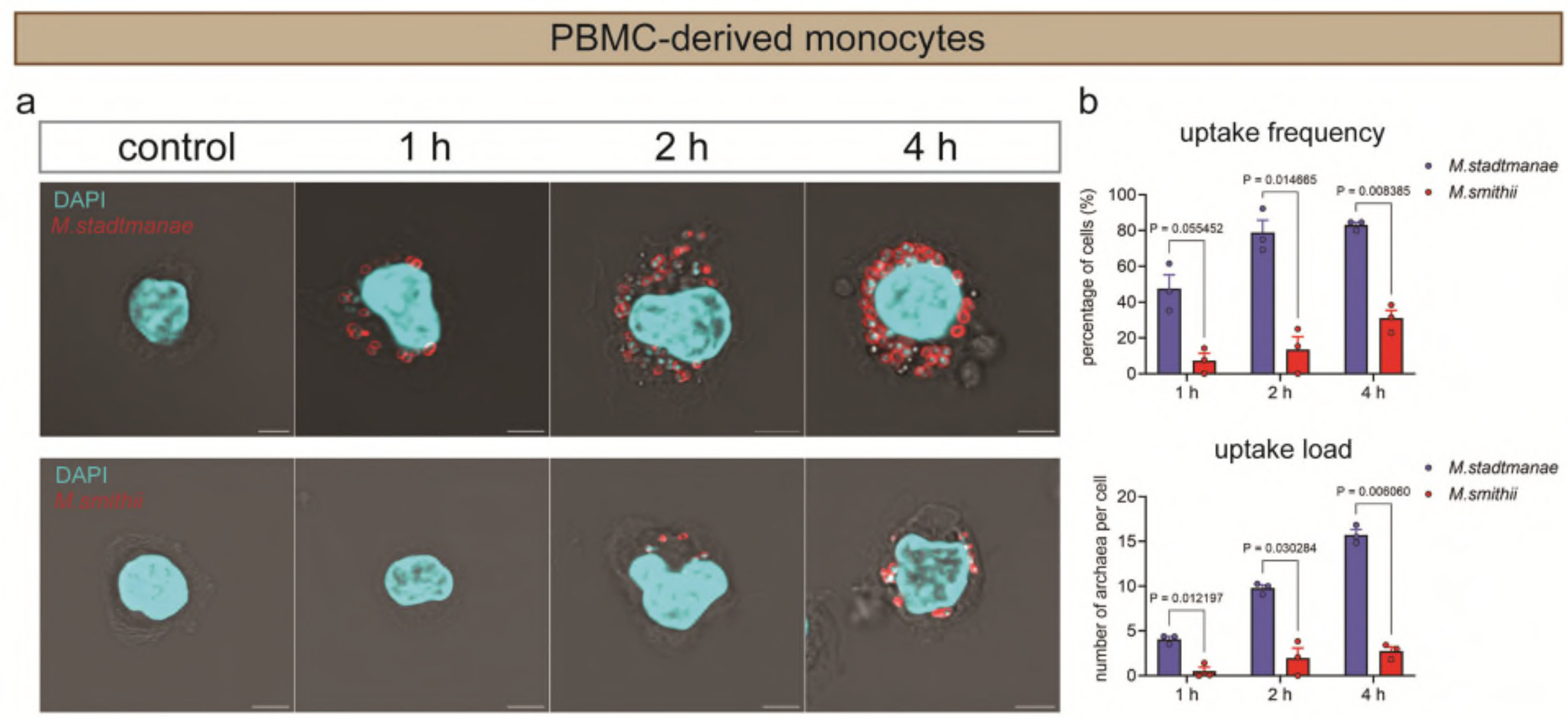

Fig. 3. a Representative merged confocal images of primary monocytes stimulated with *M. stadtmanae* or *M. smithii* for 1, 2, or 4 h, as indicated. Archaea were visualized by species-specific immunofluorescence staining and appear as red fluorescent ring-like structures. Nuclei were counterstained with DAPI (cyan). Phase-contrast images are included to show cell morphology and outline. Images were acquired with a 63× oil immersion objective. Displayed images represent enlarged views of selected regions to better visualize archaea-associated signals. Scale bar = 5 µm. **b** Quantification of archaeal uptake in primary monocytes after stimulation with *M. stadtmanae* or *M. smithii* for 1, 2, or 4 h. Upper panel, uptake frequency, defined as the percentage of cells containing at least one internalized archaeal cell. Lower panel, uptake load, defined as the mean number of internalized archaea per cell, with each ring-like structure scored as one archaeal cell. For each condition, 30-50 cells were analyzed from 3 independent donors. For each donor, data were first summarized across the analyzed cells. In the upper panel, each dot represents the uptake frequency for one donor, and in the lower panel, each dot represents the mean uptake load for one donor. Data are shown as mean ± SEM. Statistical significance was assessed using a two-tailed paired test. P values are indicated in the figure.

Did the intrinsic differences in F420 fluorescence for M. smithii and M. stadtmanae (MFI ratio M. stadtmanae/M. smithii = 0.38) affect the interpretation of FACS assessment of internalization?

Response: Yes, this intrinsic difference in F420 autofluorescence must be taken into account when interpreting the flow-cytometric uptake data. Under identical acquisition settings, free *M. smithii* showed a substantially higher basal F420 signal than free *M. stadtmanae*. Therefore, raw monocyte-associated F420 intensity cannot be interpreted as a strain-independent measure of

archaeal uptake. Importantly, however, this intrinsic difference would bias the raw signal against, rather than in favor of, detecting greater uptake of *M. stadtmanae*, because each *M. stadtmanae* cell contributes less basal F420 fluorescence than each *M. smithii* cell. To address this, we normalized the monocyte-associated F420 signal to the basal F420 signal of the corresponding free archaeal strain. After normalization, the uptake-associated signal remained clearly higher for *M. stadtmanae* than for *M. smithii*, supporting more efficient uptake of *M. stadtmanae* on a per-cell basis **(Supplementary Fig. 5 c, d)**.

The experiments with CU622 CPT9a TLR8 antagonist should include at least one negative control.

Response: We agree that appropriate controls are essential for interpreting the CU-CPT9a experiments. Unstimulated cells and cells treated with CU-CPT9a alone did not show detectable cytokine induction under our experimental conditions. To further address potential off-target effects and confirm that non-TLR8 pathways remained functional, we tested responses to agonists for other TLRs in the presence of CU-CPT9a. CU-CPT9a did not diminish responses to Pam3CSK4 (TLR2/1) or LPS (TLR4), indicating that the antagonist does not broadly suppress innate responsiveness and supporting its pathway selectivity in our system (**Supplementary Fig. 7a; newly added data**). In addition, CU-CPT9a has previously been characterized as a highly selective human TLR8 antagonist with minimal activity toward the closely related TLR7, showing >10,000-fold selectivity in TLR reporter assays and no inhibition of TLR7-driven cytokine responses in PBMC subset assays (Nat. Chem. Biol. 2018, PMID: 29155422; Cell Chem. Biol. 2018, PMID: 30100350). This data has been added to the revised manuscript **(Results, Lines 686-693)**

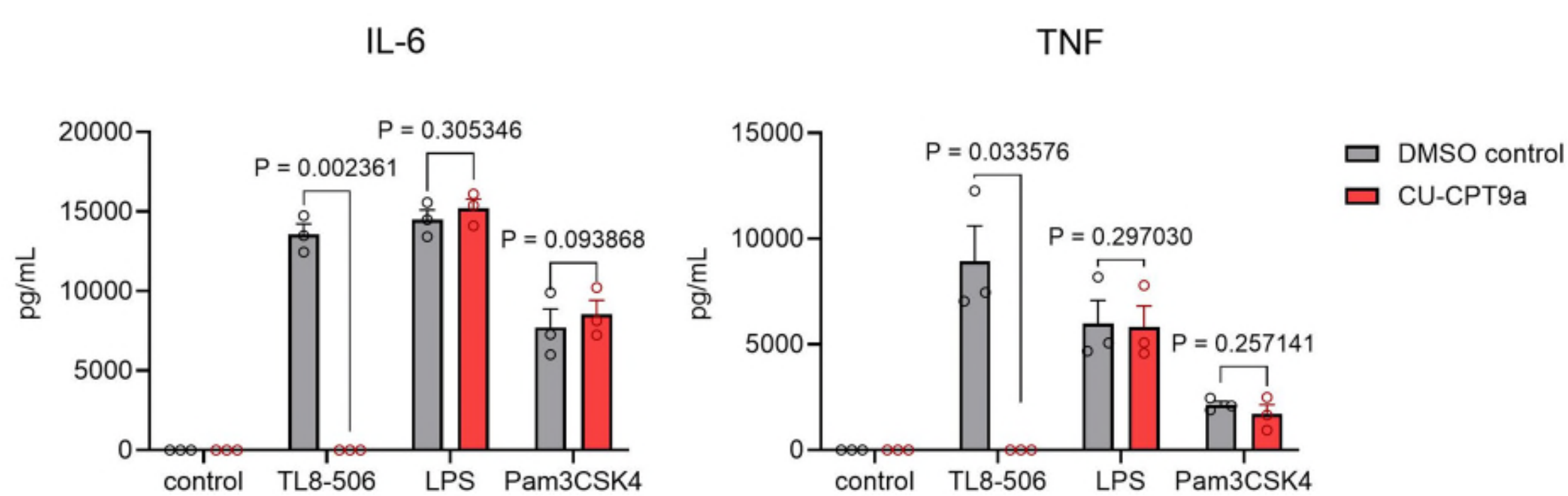

Supplementary Fig. 8a. PBMC-derived monocytes were pre-treated with the TLR8 antagonist CU-CPT9a, CU-CPT9a alone, or the corresponding vehicle control (DMSO; matched final concentration in all conditions), and subsequently stimulated as indicated. Secreted IL-6 and TNF in culture supernatants were quantified by ELISA. Data are shown as individual donors (n = 3) with summary statistics (mean ± SEM). Each donor represents an independent experimental batch. Statistical significance was assessed by multiple paired t-tests. Exact adjusted P values are indicated in the figure.

Reviewer #1 (Remarks to the Author):

The resubmitted manuscript entitled, "Differential methanogenic archaea-induced TLR8-dependent signaling is governed by NF-κB p65- and STAT1/2-controlled gene classes," by Xu et al addresses many of the questions raised by this reviewer. The central premise of the story is that there is something inherently different between RNA from *Methanosphaera stadtmanae* and *Methanobrevibacter smithii* which results in differential sensing by TLR8.

Preamble: we believe that part of the disagreement with certain points in our manuscript stems from either misunderstandings or imprecise wording on our side and we will try to clarify and explain these points.

Response: We thank the reviewer for acknowledging the revisions. We would like to clarify an important aspect of our model. The central premise of our study is not that RNA from *M. stadtmanae* is inherently different from the RNA of *M. smithii* and therefore sensed differently by TLR8. Rather, our data supports a model in which intact archaeal cells differ in their uptake by human monocytes, thereby changing the effective amount of archaeal input that becomes available to endosomal TLR8. In particular, the RNA-transfer experiments shown in Fig. 6a, b argue against the differential response being explained primarily by RNA sequence or RNA-intrinsic chemical features.

To make this model explicit, we have now added a new summary figure, Fig. 8, which illustrates three conceptual layers of the response. First, *M. stadtmanae* and *M. smithii* differ in their uptake by monocytes. Second, this differential uptake is proposed to shape uptake-dependent RNA-TLR8 ligand availability, with *M. stadtmanae* generating a stronger uptake-associated TLR8 input than *M. smithii*. Third, this TLR8 input strength is decoded into distinct transcription factor-dependent gene programs. Consistent with the archaeal RNA dose-response experiments, the STAT1/2-associated Class II program behaves as a lower-threshold module, whereas the NF-κB p65-controlled Class I program requires stronger TLR8 input.

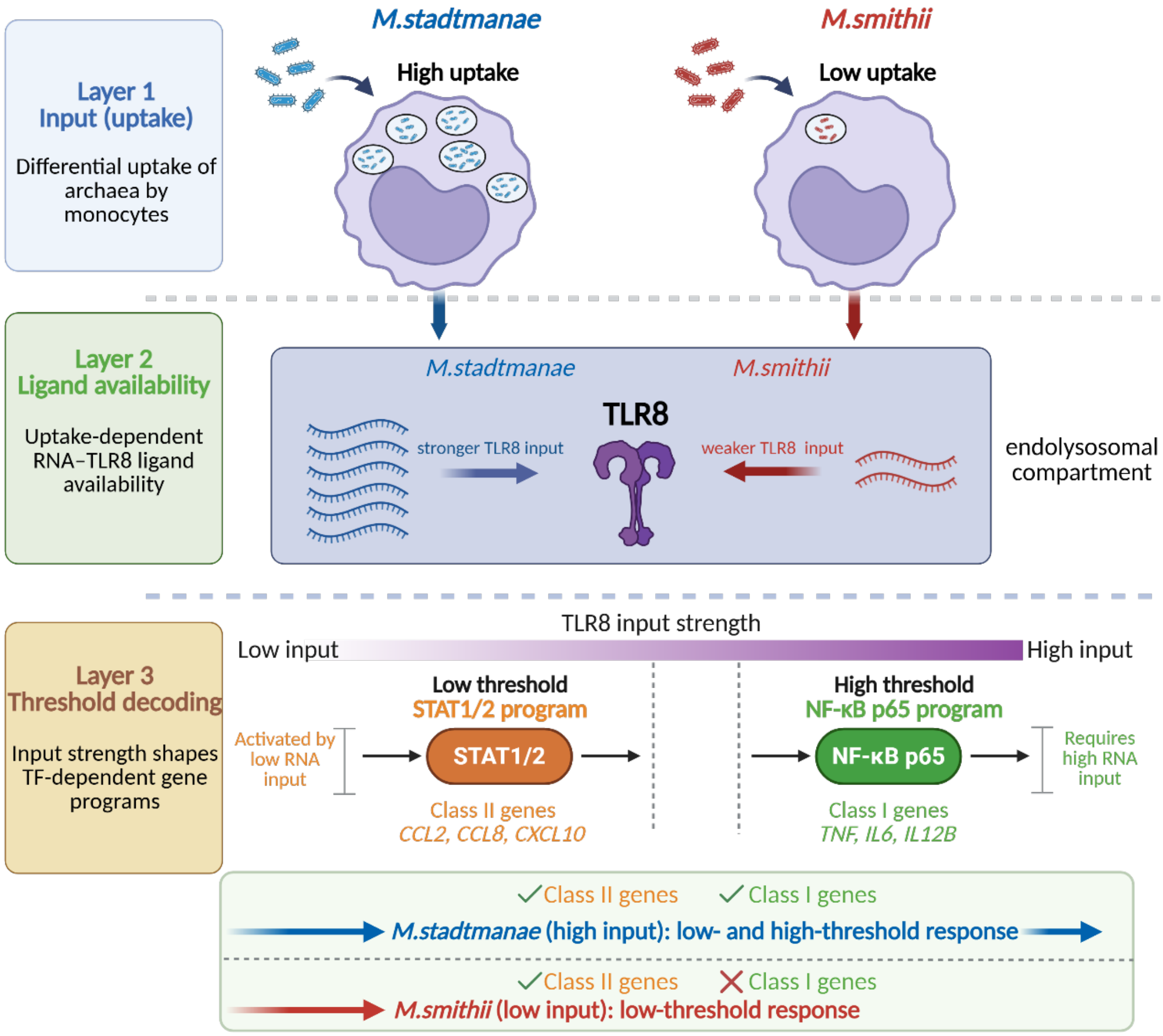

Fig. 8. Three-layer model of uptake-associated TLR8 input and threshold-dependent gene-class activation in PBMC-derived monocytes.

The model separates archaeal sensing into three layers. Layer 1 represents differential uptake of intact archaea by monocytes, with M. stadtmanae being internalized more efficiently than M. smithii. Layer 2 represents uptake-dependent RNA-TLR8 ligand availability, which is proposed to generate stronger uptake-associated TLR8 input for M. stadtmanae and weaker input for M. smithii. Layer 3 represents threshold-dependent decoding of this input into transcription factor-controlled gene programs. The STAT1/2-associated Class II program behaves as a lower-threshold module, whereas the NF-κB p65-controlled Class I program requires stronger TLR8 input. Consequently, M. stadtmanae activates both Class II and Class I genes, while M. smithii preferentially supports the lower-threshold Class II response. The model integrates the uptake data, TLR8 inhibition experiments, equal-delivery RNA experiments, RNA dose-response analyses, and p65/STAT1/2 ChIP-qPCR results. [Editorial note: Created in BioRender. Xu, F. (2026) https://BioRender.com/zucaeoc]

Their model claims that this is not due to differential cell integrity, metabolic activity or any other reason.

This reviewer is not arguing that the authors should exclude all of these possibilities. My concern is that authors have not demonstrated that their model is even feasible.

Response: We understand that the reviewer's central concern is whether our model is cell biologically feasible, in particular whether internalized intact archaea can be routed into acidified degradative compartments where archaeal RNA-derived material could become accessible to endosomal TLR8. We therefore performed a focused set of new experiments to address this feasibility issue directly, as detailed in the point-by-point responses below.

1. Inhibition of phagocytosis abrogates sensing in response to one species but not the other. Author's explanation makes sense, but they need to show that residual phagocytosis is still happening with *M. smithii*.

Response: In the ELISA experiment **(now Supplementary Fig. 7a)**, inhibition of phagocytosis virtually completely inhibited cytokine release induced by both archaeal species. Specifically, cytokine release was reduced by 94% for IL-1β and 95% for IL-6 in *M. stadtmanae*-stimulated cells, and by 100% in *M. smithii*-stimulated cells. We therefore do not interpret the data as showing that phagocytosis inhibition abrogates sensing of only one species. Instead, these data indicate that cytokine induction by both archaea is equally dependent on phagocytosis.

We note that the reviewer referred to residual phagocytosis with *M. smithii* in this context. In our ELISA data, however, *M. smithii*-induced cytokine release was no longer detectable above basal levels after cytochalasin D treatment. The residual signal relevant to this point was therefore limited to the very small remaining response observed for *M. stadtmanae* **(Supplementary Fig. 7a)**.

We therefore addressed the underlying concern by quantifying *M. stadtmanae* phagocytosis using flow cytometry, taking advantage of the intrinsic fluorescence of the archaeal cofactor F_{420}. Cytochalasin D treatment significantly reduced the archaeal F_{420} fluorescence associated with monocytes, indicating a marked decrease in the amount of internalized archaea. However, a very small population of F_{420}-positive monocytes remained detectable. Indeed, the proportion of F_{420}-positive monocytes was slightly higher after cytochalasin D treatment than under control conditions (5.48% versus 1.52%), despite the substantially lower archaeal fluorescence signal within these cells. This apparent discrepancy suggests that cytochalasin D strongly reduced the archaeal burden but did not eliminate all residual cell-associated events. Consistent with this interpretation, the remaining archaeal signal was accompanied by only a greatly reduced residual cytokine response.

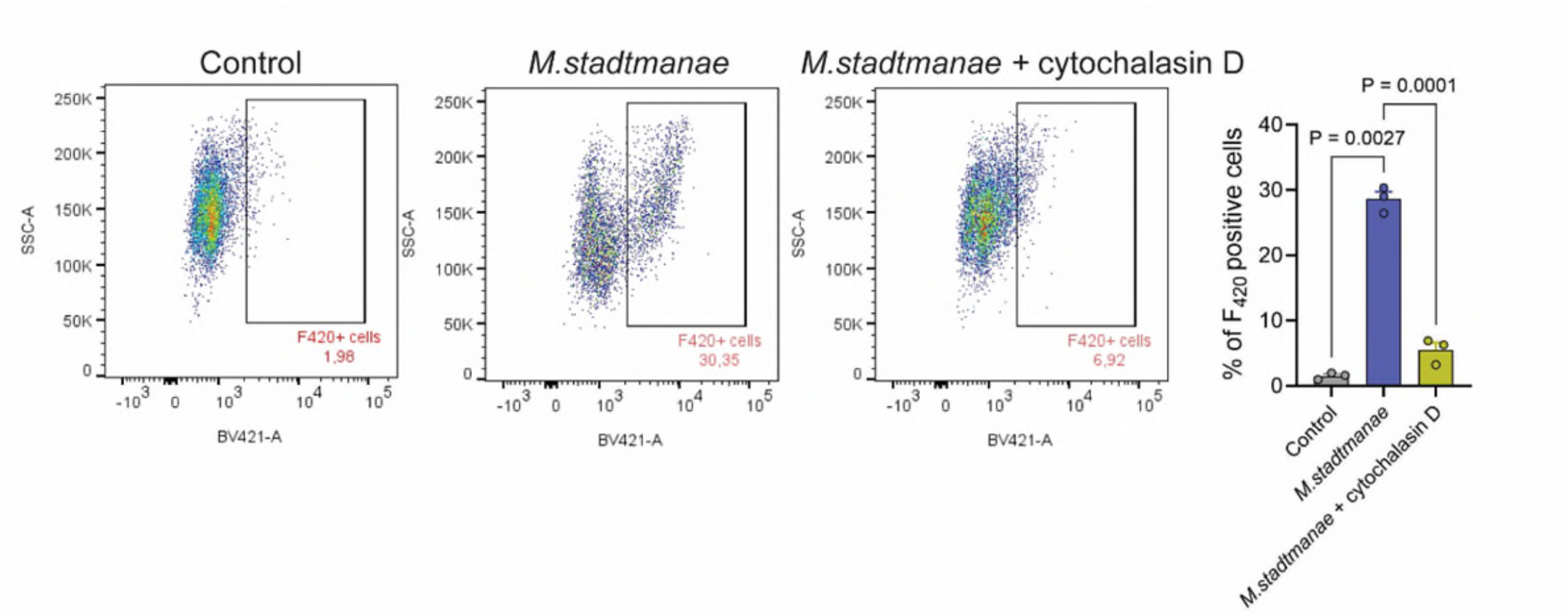

Flow-cytometric assessment of M. stadtmanae uptake by monocytes. *Monocytes were stimulated with M. stadtmanae for 4 h in the presence or absence of the phagocytosis inhibitor cytochalasin D. Archaeal uptake was assessed by flow cytometry based on the intrinsic fluorescence of the archaeal cofactor F_{420}. Representative flow-cytometry plots and quantification of monocyte-associated F_{420} fluorescence intensity and the percentage of F_{420}-positive monocytes are shown. Each data point represents one independent donor (n = 3). Statistical significance was assessed using one-way ANOVA followed by Dunnett's multiple-comparisons test; exact P values are indicated in the figure.*

Also, if *M. smithii* is being taken up more efficiently, why does it have a weaker effect on pro-inflammatory gene expression?

Response: We appreciate this point and would like to clarify the direction of the uptake difference. In our experiments, the higher uptake was observed for *M. stadtmanae*, not for *M. smithii* (Fig. 3). Thus, the weaker pro-inflammatory response induced by *M. smithii* is consistent with its lower uptake in our assay and with our proposed model.

2. They have not shown anything inherently different between RNA from the two archaeal species. Their RNA transfection experiments show no difference between the two species. This can be explained by the wrong usage of reagents (see belo), but they have also not provided how TLR8 is able to access. They "clarify" at several places that TLR8 is intracellular, which it is, but it is sensing extracellular danger coming in through endocytosis. The lumen of the endosome contains extracellular material that have been endocytosed/phagocytosed. This is the reason why it is well accepted that all endosomal sensors sense danger coming from the outside. This is the reason why this reviewer used the sentence, "TLR8 is facing outside the cell." Unfortunately, the idea that endosomal TLRs sense intracellular danger is a common mistake I see with first year graduate students when I teach innate immune sensing. But teaching is not my job here, and the only reason why I am pointing this out is that authors have come back to this point over and over again in their attempt to prove that their model is correct. I will now explain why my concerns have not been addressed by the authors on this point. I am copying

exact words (bold is coming from the authors) from the point by point response so that the editor can see where my concern is coming from: ***"However, our model does not require extracellular rupture of archaeal cells in the culture medium."*** *In this model, intact archaea are internalized and processed in endosomal or endo-lysosomal compartments, where their RNA becomes available for TLR8 sensing. This is consistent with* ***the subcellular localization of TLR8 as an endosomal, rather than cell-surface, RNA sensor****. Together, these findings indicate that innate immune sensing of methanogens in our system depends on uptake and intracellular processing rather than spontaneous extracellular rupture of archaeal cells.*" As can be seen in the included figure above, without cell rupture RNA will not be accessible to TLR8 for binding. The cell rupture does not have to be extracellular. It is possible that differential cell ruptures happen in the endosome or during phagocytosis. I am not sure what they mean by "processed" in their response. Do they mean that archaeal RNA is processed and delivered to TLR8 without exiting the archaeal cell? If there is no chemical difference in RNA from the two species, as the authors are claiming, then the amount of RNA available for sensing by TLR8 has to differ to have a differential effect on host gene expression.

Response: We thank the reviewer for clarifying his points. We also now understand the reviewer's statement that TLR8 is "facing outside the cell" to mean that TLR8 senses extracellular material/danger after it has been taken up into the endosomal/phagolysosomal lumen. Our intention was not to suggest that TLR8 senses cytosolic or host-intracellular danger signals. Rather, when we referred to intracellular sensing, we meant that the sensing occurs within intracellular vesicular compartments of the host cell. In this context, the relevant ligand is microbial single-stranded RNA, which becomes accessible to TLR8 only after archaeal material has entered and is processed within the endosomal/phagolysosomal lumen. We have revised the wording to avoid this ambiguity.

By "processed", we simply meant that once archaea are taken up, they enter the phagosomal/endolysosomal compartments and there the engulfed archaeal cells have to be damaged, ruptured, or degraded, in order to allow archaeal RNA or RNA-derived products to become available to TLR8 in the vesicle lumen. We do not mean that TLR8 senses RNA that is still trapped inside an intact archaeal cell.

To address this feasibility issue directly, we added new experiments to examine whether internalized archaea traffic into acidified endolysosomal/phagolysosomal compartments, a compartmental route in which microbial degradation could facilitate RNA processing and exposure of RNA ligands for TLR8 sensing. LysoTracker Green DND-26 labels acidic

intracellular compartments and therefore reports the acidified vesicular environment in which engulfed microbial material can be degraded. Bafilomycin A1 inhibits vacuolar H+-ATPase activity and blocks endosomal/lysosomal acidification, allowing us to test whether this acidified compartmental step is required for archaeal-induced pro-inflammatory gene expression. LAMP1 is a marker of late endosomal/lysosomal and phagolysosomal membranes and was used to assess whether internalized archaeal cells are associated with late endosomal/lysosomal or phagolysosomal compartments.

Using these complementary approaches, we found that both archaeal species induced LysoTracker-positive acidified compartments, with a stronger signal after *M. stadtmanae* stimulation than after *M. smithii* stimulation **(Supplementary Fig. 9a, b)**. This pattern is consistent with the higher phagocytic uptake of *M. stadtmanae* observed above. Bafilomycin A1 abolished the LysoTracker signal and markedly decreased archaeal-induced TNF and IL-6 release, while not impairing archaeal uptake **(Supplementary Fig. 9a-d)**. In parallel, confocal imaging showed that internalized archaeal cells partially overlapped with and/or were enclosed by LAMP1-positive membrane structures, consistent with trafficking into LAMP1-positive late endosomal/lysosomal or phagolysosomal compartments **(Supplementary Fig. 9f)**.

Finally, to address the reviewer's point that the amount of RNA available for TLR8 sensing should differ if the RNAs themselves are not intrinsically different, we quantified archaeal RNA in extensively washed monocyte-associated fractions. Species-specific RT-qPCR detected archaeal *rpoB* RNA, with a higher signal after *M. stadtmanae* than after *M. smithii* stimulation **(Supplementary Fig. 9e)**. Together, these results support a model in which more efficient uptake of *M. stadtmanae* increases its entry into acidified endolysosomal/phagolysosomal compartments, where acidification-dependent microbial processing can promote TLR8-dependent cytokine induction.

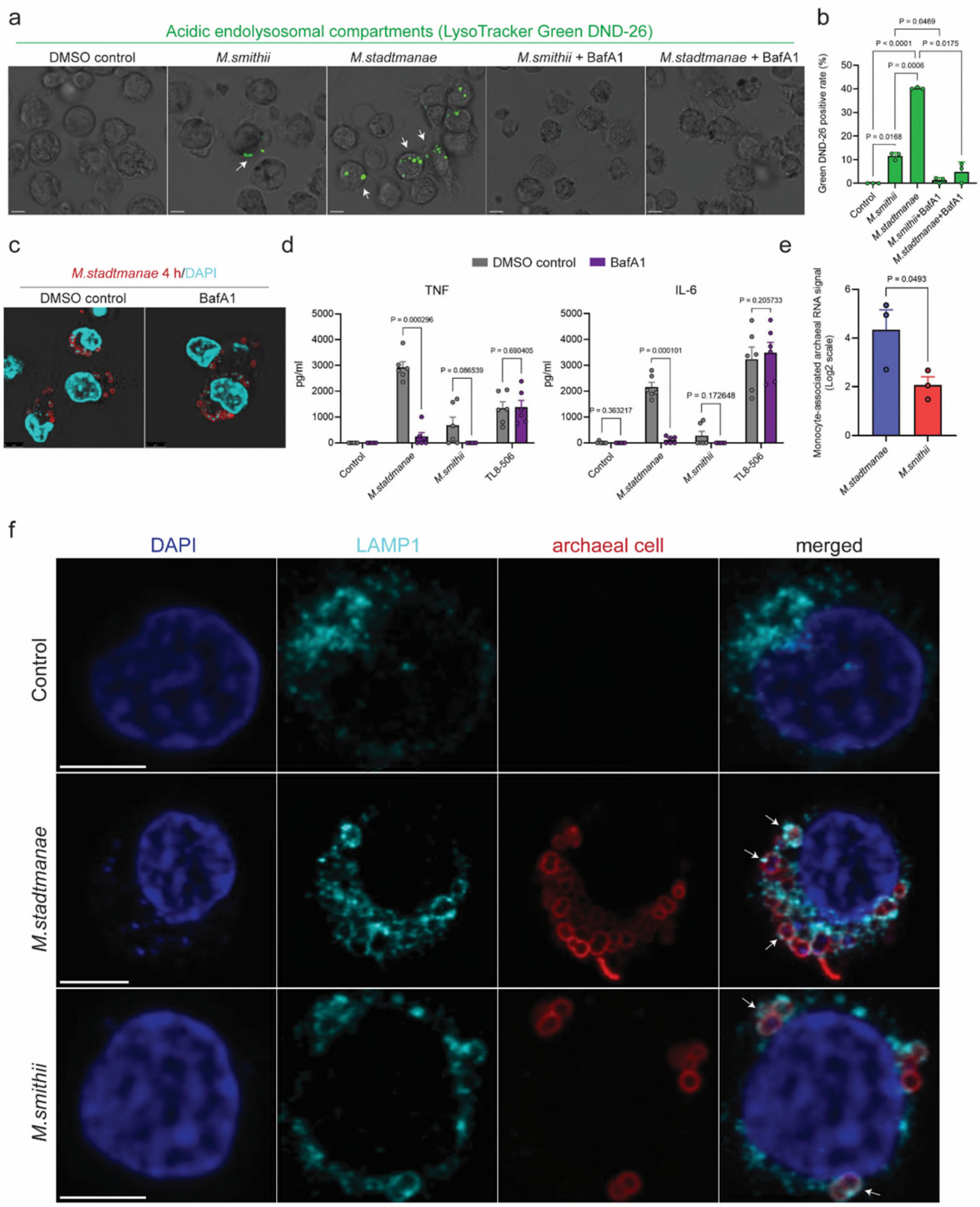

Supplementary Fig. 9. Endolysosomal pathway of internalized archaea in human monocytes

a *Representative images of LysoTracker Green DND-26 staining in PBMC-derived monocytes left unstimulated or stimulated with M. smithii or M. stadtmanae for 4 h, in the presence of vehicle control (DMSO) or bafilomycin A1 (BafA1). Green puncta indicate acidic endolysosomal compartments.* ***b*** *Quantification of LysoTracker Green DND-26-positive monocytes under the indicated conditions. Each dot represents one donor, corresponding to one independent donor (n = 3). Data are shown as mean ± SD. Statistical significance was assessed using one-way ANOVA followed by Tukey's multiple comparisons test.* ***c*** *Representative immunofluorescence images showing M. stadtmanae uptake in monocytes treated with DMSO or BafA1. Archaea are shown in red and nuclei were counterstained with DAPI.* ***d*** *ELISA quantification of TNF and IL-6 in culture supernatants from monocytes stimulated with M.*

stadtmanae, M. smithii, or TL8-506 in the presence of DMSO or BafA1. Each dot represents one donor, corresponding to one independent donor (n = 6). Data are shown as mean ± SEM. Statistical significance was assessed using paired two-tailed t-test for two-group comparisons. ***e*** *Species-specific RT-qPCR detection of monocyte-associated archaeal rpoB RNA after extensive washing. rpoB RNA levels were normalized to the host reference gene HPRT, and the normalized signal is shown on a log2 scale. Each dot represents one donor, corresponding to one independent donor (n = 3). Data are shown as mean ± SD. Statistical significance was assessed using paired two-tailed t-test for two-group comparisons.* ***f*** *Representative confocal images showing LAMP1-positive compartments and cell-associated archaea in unstimulated monocytes or monocytes stimulated with M. stadtmanae or M. smithii. Some internalized archaea were surrounded by LAMP1-positive structures, with partial colocalization observed in selected regions indicated by arrows. Scale bars, 5 µm. Exact P values are indicated in the figure.*

3. The claim that DOTAP can be used to specifically deliver RNA into endosomes is patently wrong. DOTAP is often used cytosolic delivery of proteins into the cytosol. In fact, it is one of the most widely used reagents for cytosolic delivery of bacterial flagellin to activate the NLRC4 inflammasome. Also, RNA sensing by cytosolic RNA sensors might be masking the effect of TLR8. If authors were not using DOTAP, they might even see a difference between the two RNA. The strength of pro-inflammatory gene expression will be weaker but that does not excuse using wrong methodologies. One way to efficiently engage endosomal TLRs is to use very high concentrations of their ligands. For example, 100 – 200 µg/mL poly(I:C) is used to activate TLR3. I cannot recommend acceptance of a manuscript that claims that DOTAP can be used for endosomal delivery of RNA cargo. This will only muddy the field further. Just like the authors are citing previously published papers, future authors will cite this paper to claim that DOTAP can be used to specifically deliver RNA into endosomes. Here are a few references to show that DOTAP delivers its cargo to the cytosol:

1. https://www.nature.com/articles/ni1346

2. https://www.nature.com/articles/s41596-020-0374-9

3. https://www.nature.com/articles/s41578-021-00358-0

Response: We agree that DOTAP should not be described as a reagent that specifically delivers RNA into endosomes. If our previous wording gave that impression, we apologize for the ambiguity. In the manuscript, we actually did not use the term "specifically" in this context, nor did we intend to state that DOTAP selectively targets RNA to endosomal compartments. Our use of DOTAP had a more limited purpose: to compare the intrinsic stimulatory activity of purified RNA from *M. stadtmanae* and *M. smithii* under equal-delivery conditions. We did not intend to use DOTAP to define the physiological route by which intact archaea deliver RNA to TLR8. Under these equal-delivery conditions, RNA from the two archaeal species induced

comparable responses, arguing against the idea that *M. stadtmanae* RNA is intrinsically more potent than *M. smithii* RNA.

At the same time, we would like to clarify that cytosolic delivery and an endocytic/endosomal trafficking step are not mutually exclusive. We agree with the reviewer that DOTAP can also promote cytosolic delivery of cargo. However, this does not necessarily mean that DOTAP-RNA bypasses endocytic or endosomal compartments. Lipid-based delivery systems, including DOTAP-containing lipoplexes, are commonly internalized through endocytic pathways and pass through endosomal compartments before cargo release or endosomal escape. This route is supported by studies on lipid-based nucleic acid delivery, including DOTAP-containing lipoplexes (for example also the third paper mentioned by this reviewer, Hou et al., *Nat. Rev. Mater.*, 2021; but likewise, Rejman et al., *Mol. Ther.*, 2005; Caracciolo et al., *J. Phys. Chem. B*, 2009; Sahay et al., *J. Control. Release*, 2010). Therefore, the ability of DOTAP to support cytosolic delivery does not exclude at all a transient endosomal step during which RNA may become accessible to TLR8.

This is also consistent with previous RNA-TLR7/8 stimulation studies, including the original report by Heil et al. (Science, 2004), where DOTAP-complexed ssRNA was used to induce TLR7/8-dependent immune activation. Similar DOTAP-complexed RNA approaches were later used in functional TLR7/8 assays, including, among many others, studies by Colak et al. (J Immunol., 2014), Herster et al. (Nat. Commun., 2020), and Alharbi/Sapkota et al. (Nat. Immunol., 2026). We cite these studies to support the use of DOTAP as an artificial RNA delivery approach in functional RNA-TLR7/8 assays, not to claim that DOTAP specifically delivers RNA into endosomes.

To assess whether DOTAP-complexed RNA stimulation engages acidified compartments under our assay conditions, we first examined LysoTracker Green DND-26 staining. DOTAP-complexed archaeal RNA increased the proportion of LysoTracker-positive monocytes, and this signal was abolished by bafilomycin A1 **(Supplementary Fig. 12a)**, confirming effective inhibition of acidified compartment formation or maintenance.

As an additional positive-control RNA ligand, we used Cy5-labeled ssRNA40, a GU-rich synthetic single-stranded RNA commonly used as a human TLR8-activating RNA ligand when delivered intracellularly. DOTAP-complexed Cy5-labeled ssRNA40 showed detectable colocalization with LysoTracker Green-positive acidic compartments, supporting the presence of DOTAP-complexed RNA within TLR8-relevant endolysosomal environments **(Supplementary Fig. 12b)**. We do not interpret these data as evidence for exclusive endosomal

delivery by DOTAP, but they show that DOTAP-complexed RNA can access acidified compartments under our experimental conditions.

Functionally, bafilomycin A1 reduced cytokine induction by both DOTAP-complexed archaeal RNA and DOTAP-complexed Cy5-labeled ssRNA40 **(Supplementary Fig. 12c, d)**, and TLR8 inhibitor CU-CPT9a suppressed the Cy5-labeled ssRNA40 response (**Supplementary Fig. 12d)** as well as DOTAP-complexed archaeal RNA responses (**Supplementary Fig. 11c)**. These data do not establish exclusive endosomal delivery by DOTAP. They indicate that, under our assay conditions, DOTAP-complexed RNA can be detected in acidified compartments and that the induced cytokine response is sensitive to bafilomycin A1 and TLR8 inhibition.

In addition, as supportive data from intact archaeal stimulation, deletion of cytosolic RNA receptors RIG-I, MAVS, or STING did not reduce the response to *M. stadtmanae* (see below and PhD thesis of our former PhD student Tim Vierbuchen, https://macau.uni-kiel.de/servlets/MCRFileNodeServlet/dissertation_derivate_00007807/PhD_Thesis_Vierbuchen.pdf).

Together, these data support the interpretation that, under our assay conditions, DOTAP-delivered RNA engages an acidified endolysosomal route that culminates in TLR8-dependent cytokine induction.

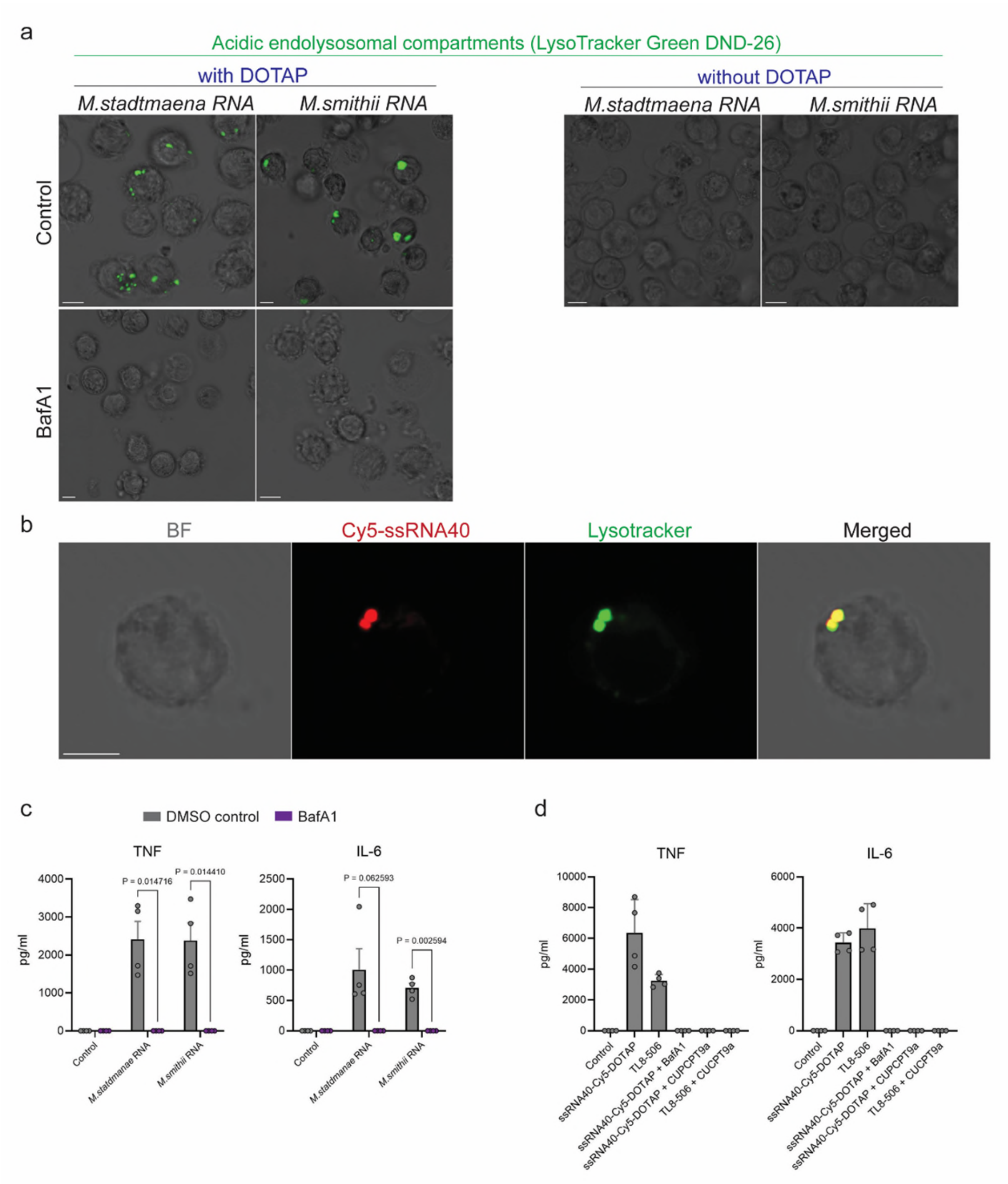

Supplementary Fig. 12. DOTAP-complexed RNA accesses acidified endolysosomal compartments and induces acidification- and TLR8-dependent cytokine responses.

a *LysoTracker Green staining of acidic compartments in monocytes treated with DOTAP-complexed archaeal RNA (1 µg ml^{-1}) in the presence or absence of bafilomycin A1. Representative images are shown.* ***b*** *Representative fluorescence images showing the intracellular localization of DOTAP-complexed Cy5-labelled ssRNA40 (1 µg ml^{-1}) and LysoTracker Green-positive acidic compartments.* ***c*** *Cytokine responses induced by DOTAP-complexed archaeal RNA (1 µg ml^{-1}) in the presence or absence of bafilomycin A1. Each dot represents one individual donor, with each donor representing an independent donor (n = 4). Data are shown as mean ± SEM. Statistical significance was assessed using paired two-tailed t-test for two-group comparisons.* ***d*** *Cytokine responses induced by DOTAP-complexed Cy5-*

labelled ssRNA40 (1 μg ml^{-1}) in the presence or absence of bafilomycin A1 or the TLR8 inhibitor CU-CPT9a. Each data point represents one biologically independent donor (n =4).

[editorial note: third party material redacted]

Reviewer #2 (Remarks to the Author):

The authors have responded to the critiques with adequate additional data and reasonable counterarguments. The amount of work that has gone into this study is to be commended.

The intrinsic limitations of this study remain: it straddles two interesting findings without going deeply into either. The first finding is that M. smithii has lower uptake in phagocytes than M. stadtmanae, resulting in weaker induction of some genes ("class I"). The second finding is that M. stadtmanae preferentially induces NFkB but not IRF target genes at the same dose as M. smithii. Neither finding is given a fully satisfying mechanistic explanation, and the results of the new experiments (especially different doses of archaea and TLR8 agonist alone) did not shed much light on the mechanistic details.

That being said, I believe the authors are aware of the limitations of the study and do reasonably consider them in the discussion. Ideally, the manuscript could be made more concise for improved readability.

Response: We thank the reviewer for this constructive and balanced comment, and for acknowledging the additional work included in the revision. We agree that our study does not explain every mechanistic detail behind the differential uptake of the two archaea or the different balance of NF-κB- and IRF/STAT-associated responses. Still, our study shows not only that human-associated archaea can drive different TLR8-dependent gene programs in human monocytes but also, in more general aspects, that RNA threshold-dependently drives functionally different gene programs through TLR8 engagement.

However, we would like to point out that *M. stadtmanae* shows higher NF-kB p65 dependent genes induction but with respect to IRF target genes (part of class II genes in our study), both *M. stadtmanae* and *M. smithii* are equally capable.

To strengthen the mechanistic link between archaeal uptake and the weaker *M. smithii*-induced Class I response, we added two targeted experiments. We further examined the effect of phagocytosis inhibition on archaeal-induced cytokine release. In the new ELISA experiment, cytochalasin D almost completely abolished *M. stadtmanae*-induced IL-1β and IL-6 release and eliminated the weaker *M. smithii*-induced response. These data support the conclusion that cytokine induction by both archaeal species is strongly dependent on phagocytosis, and that the species-specific difference is lost when uptake is inhibited.

We also revisited whether increasing *M. smithii* input can compensate for its weaker response using a more direct early transcriptional readout. The previous dose-escalation experiment was based on 24 h cytokine accumulation, which can be influenced by secretion kinetics, feedback, and saturation. We therefore stimulated cells for 4 h and quantified representative Class I transcripts by RT-qPCR. Under these conditions, a two-fold increase in *M. smithii* input partially increased the early induction of representative Class I genes, but the response still remained below that induced by *M. stadtmanae*. In contrast, increasing *M. smithii* input ten-fold, to 10^8 cells/mL, raised Class I gene induction to a level comparable to that induced by *M. stadtmanae* at 10^7 cells/mL. This supports the interpretation that the weaker Class I response to *M. smithii* is input-limited and is consistent with an uptake-dependent increase in intracellular archaeal material available for sensing.

We have also streamlined parts of the Results to improve readability. We have kept the model deliberately restrained and do not claim that these experiments fully define the molecular identity of the intracellular ligand or all downstream decoding mechanisms.

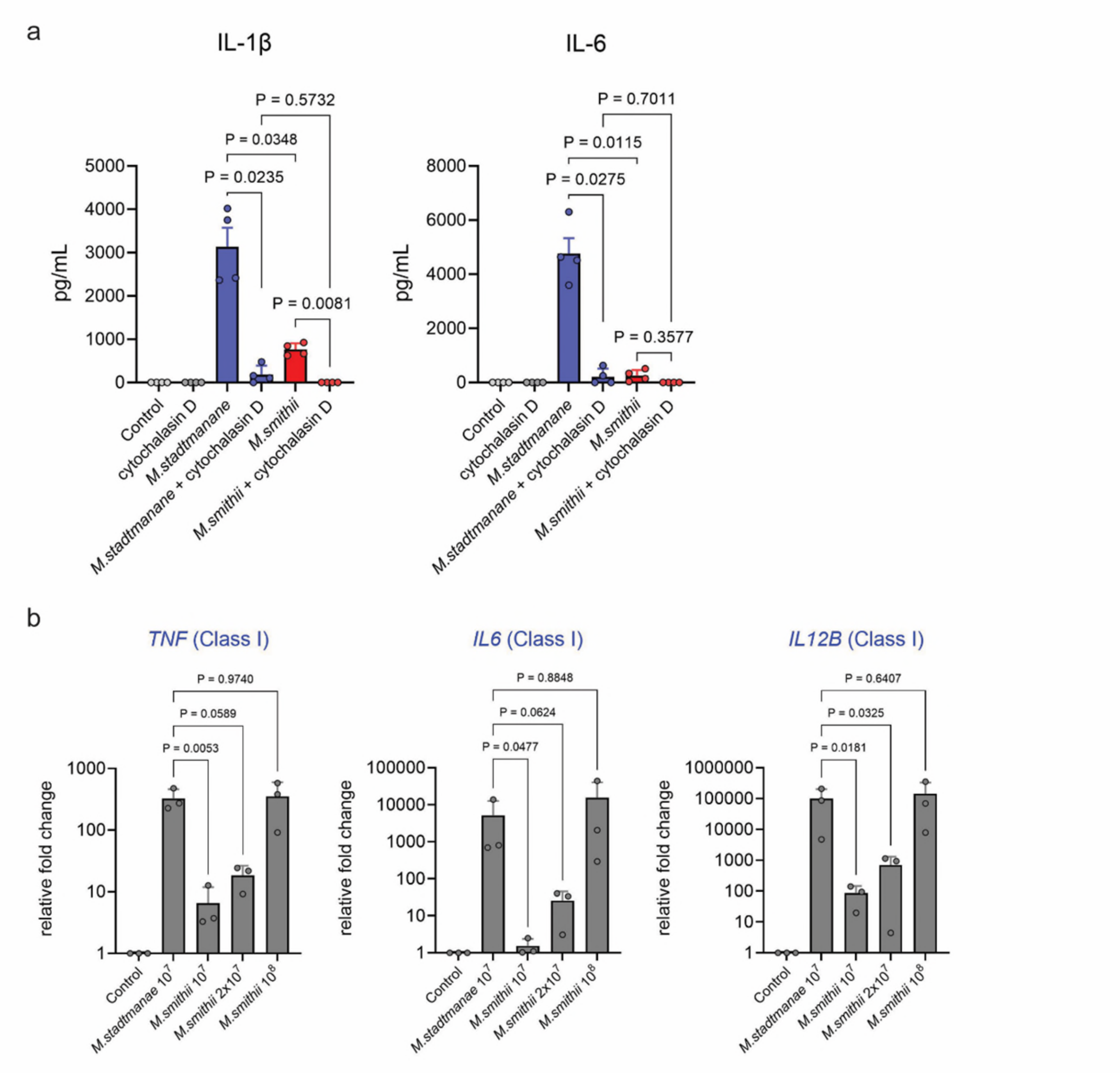

Supplementary Fig. 7. Phagocytosis-dependent cytokine release and dose-dependent class I gene induction in archaeal-stimulated human monocytes

a ELISA was used to quantify IL-1β and IL-6 in culture supernatants from monocytes stimulated with *M. stadtmanae* or *M. smithii* in the presence or absence of the phagocytosis inhibitor cytochalasin D. Each data point represents one donor and one independent biological donor (n = 4). Data are shown as mean ± SEM. Statistical significance was assessed using one-way ANOVA followed by Tukey's multiple comparisons test. Exact P values are shown in the figure. **b** RT-qPCR analysis of the expression of selected class I genes in PBMC-derived monocytes stimulated for 4 h with *M. stadtmanae* at 10^7 cells ml^-1 or with *M. smithii* at 10^7, 2 × 10^7, and 10^8 cells ml^-1. Gene expression levels are presented as fold induction relative to control, normalized to *HPRT*, with the y-axis shown on a $\log_{10}$ scale to reflect the magnitude of induction. Each dot represents one donor, corresponding to one independent donor (n = 3). Data are shown as mean ± SD. Statistical significance was assessed using one-way ANOVA followed by Dunnett's multiple comparisons test versus the control condition. Exact P values are indicated above the bars.

REVIEWERS' COMMENTS

Reviewer #1 (Remarks to the Author):

Authors have addressed all my concerns. I recommend acceptance. I also want to thank them for a constructive response to my critiques.
This is not a critique for this manuscript but a suggestion for the future: the data in the manuscript is also compatible with a model where a chemical difference (maybe an RNA modification) between archaeal RNA and mammalian RNA makes some form(s) of archaeal RNA a TLR8 ligand. If it turns out to be true, authors would have made another truly fundamental contribution to the field of innate immunity.

Response: We sincerely thank the reviewer for the positive assessment of our revised manuscript and for the constructive comments throughout the review process. We also appreciate this insightful suggestion. We agree that chemical features of archaeal RNA, including potential RNA modifications, may contribute to its recognition by TLR8 and represent an important direction for future investigations. We will explore this possibility in future studies.

Reviewer #2 (Remarks to the Author):

The authors have provided the needed data to support their claims. The additional dose response data in Supplemental Figure 7 is a strength, and the addition of Figure 8 clearly presents the main findings: M. smithii is taken up less efficiently and activates TLR8 less strongly than M. stadtmanae, and weak vs strong induction of TLR8 produces distinct gene expression programs driven by STATs and NFkB, respectively. These findings are supported by an extensive amount of experimental work. The study leaves two intriguing questions that deserve to be investigated in follow-up work: 1) Why is M. smithii phagocytosed less efficiently than M. stadtmanae? 2) Why does weak TLR8 activation favor STAT signaling over NFkB signaling?

My only feedback to improve the manuscript at this time are:
- Figure 8 would be better if the third layer was flipped, so that "high input" is on the left and "low input" is on the right, so that this layer is aligned vertically with the two species of archaea
- The manuscript is very long; it would improve readability if the authors can find places to make the writing more concise, especially in the discussion

Overall, I have no remaining concerns about the scientific rigor or the interpretation of the data. The central question for the editor to decide is whether the significance of this work is suitable for this journal.

Response: We thank the reviewer for the positive assessment and constructive suggestions. We also appreciate the two interesting questions raised for future investigation regarding differential archaeal uptake and input-dependent STAT versus NF-κB signaling, both of which we are already investigating.

In response to the reviewer's specific suggestions, we have revised Fig. 8 by rearranging Layers 1 and 2 so that the high- and low-input branches are vertically aligned with the corresponding archaeal species and with Layer 3. We have already further streamlined the manuscript to improve readability, particularly by shortening and simplifying the Discussion in the last revision.

The resubmitted manuscript entitled, "Differential methanogenic archaea-induced TLR8-dependent signaling is governed by NF-κB p65- and STAT1/2-controlled gene classes," by Xu et al addresses many of the questions raised by this reviewer. The central premise of the story is that there is something inherently different between RNA from *Methanosphaera stadtmanae* and *Methanobrevibacter smithii* which results in differential sensing by TLR8. Their model claims that this is not due to differential cell integrity, metabolic activity or any other reason.

This reviewer is not arguing that the authors should exclude all of these possibilities. My concern is that authors have not demonstrated that their model is even feasible.

1. Inhibition of phagocytosis abrogates sensing in response to one species but not the other. Author's explanation makes sense, but they need to show that residual phagocytosis is still happening with *M. smithii*.

   Also, if *M. smithii* is being taken up more efficiently, why does it have a weaker effect on pro-inflammatory gene expression?

2. They have not shown anything inherently different between RNA from the two archaeal species. Their RNA transfection experiments show no difference between the two species. This can be explained by the wrong usage of reagents (see below), but they have also not provided how TLR8 is able to access. They "clarify" at several places that TLR8 is intracellular, which it is, but it is sensing extracellular danger coming in through endocytosis. The lumen of the endosome contains extracellular material that have been endocytosed/phagocytosed. This is the reason why it is well accepted that all endosomal sensors sense danger coming from the outside. This is the reason why this reviewer used the sentence, "TLR8 is facing outside the cell." Unfortunately, the idea that endosomal TLRs sense intracellular danger is a common mistake I see with first year graduate students when I teach innate immune sensing. But teaching is not my job here, and the only reason why I am pointing this out is that authors

[editorial note: figure redacted]

have come back to this point over and over again in their attempt to prove that their model is correct.
I will now explain why my concerns have not been addressed by the authors on this point. I am copying exact words (bold is coming from the authors) from the point by point response so that the editor can see where my concern is coming from:

“*However, our model does not require extracellular rupture of archaeal cells in the culture medium.”* *In this model, intact archaea are internalized and processed in endosomal or endo-lysosomal compartments, where their RNA becomes available for TLR8 sensing. This is consistent with* ***the subcellular localization of TLR8 as an endosomal, rather than cell-surface, RNA sensor****. Together, these findings indicate that innate immune sensing of methanogens in our system depends on uptake and intracellular processing rather than spontaneous extracellular rupture of archaeal cells.*”

As can be seen in the included figure above, without cell rupture RNA will not be accessible to TLR8 for binding. The cell rupture does not have to be extracellular. It is possible that differential cell ruptures happen in the endosome or during phagocytosis.

I am not sure what they mean by “processed” in their response. Do they mean that archaeal RNA is processed and delivered to TLR8 without exiting the archaeal cell?

If there is no chemical difference in RNA from the two species, as the authors are claiming, then the amount of RNA available for sensing by TLR8 has to differ to have a differential effect on host gene expression.

3. The claim that DOTAP can be used to specifically deliver RNA into endosomes is patently wrong. DOTAP is often used cytosolic delivery of proteins into the cytosol. In fact, it is one of the most widely used reagents for cytosolic delivery of bacterial flagellin to activate the NLRC4 inflammasome.

   Also, RNA sensing by cytosolic RNA sensors might be masking the effect of TLR8. If authors were not using DOTAP, they might even see a difference between the two RNA. The strength of pro-inflammatory gene expression will be weaker but that does not excuse using wrong methodologies.

   One way to efficiently engage endosomal TLRs is to use very high

concentrations of their ligands. For example, 100 – 200 µg/mL poly(I:C) is used to activate TLR3.

I cannot recommend acceptance of a manuscript that claims that DOTAP can be used for endosomal delivery of RNA cargo. This will only muddy the field further. Just like the authors are citing previously published papers, future authors will cite this paper to claim that DOTAP can be used to specifically deliver RNA into endosomes. Here are a few references to show that DOTAP delivers its cargo to the cytosol:

1. https://www.nature.com/articles/ni1346
2. https://www.nature.com/articles/s41596-020-0374-9
3. https://www.nature.com/articles/s41578-021-00358-0
